# Mutant-Selective Binding of *Phyllanthus niruri* Phytochemicals to EGFR T790M: A Quantum-Classical Mechanistic Study

**DOI:** 10.3390/ijms27156568

**Published:** 2026-07-23

**Authors:** William D. Lituma-González, Diksha Dinesh Kumar, Amogh V. Arunprasad, Tanishque Verma, Shrutika Pillai, Fabian R. Jimenez, Sasikumar J. Mahalingam, Shanmugamurthy Lakshmanan

**Affiliations:** 1 Department of Integrative and Transdisciplinary Pharmacognosy, Siddha Vetha University, 211 Warren st, Newark, NJ 07103, USA; william.lituma@yachaytech.edu.ec (W.D.L.-G.); dikshadk2020@gmail.com (D.D.K.); amoghvarunprasad@gmail.com (A.V.A.); tanishque.verma@siddhavethamultiversity.org (T.V.); fabian.jimenez@nfinnovations.com (F.R.J.); 2 Department of Neuroscience, University of Virginia, P.O. Box 400224, Charlottesville, VA 22904, USA; sruthika.pillai123@gmail.com; 3 Department of Microbiology, Karpagam Academy of Higher Education, Coimbatore 641021, India; sasikumar.jmahalingam@kahedu.edu.in

**Keywords:** hepatocellular carcinoma, molecular dynamics simulation, MM-PBSA, per-residue energy decomposition, conceptual DFT, free energy profiling, gatekeeper resistance, kinase inhibitor resistance

## Abstract

Epidermal growth factor receptor (EGFR) mutations drive hepatocellular carcinoma (HCC) progression, and the T790M gatekeeper substitution is the predominant mechanism of acquired resistance to EGFR-targeted therapies. Herein, we present multiscale quantum-classical *in silico* predictions of *Phyllanthus niruri* phytochemicals as non-covalent EGFR T790M binders, employing molecular docking, 100 ns molecular dynamics, MM-PBSA/MM-GBSA, per-residue decomposition, PCA/LDA, DFT at B3LYP-D3(BJ)/def2-TZVP, and comparative wild-type EGFR simulations. Five phytochemicals exhibited computationally predicted binding affinities against EGFR T790M exceeding the non-covalent binding component of osimertinib (−25.74 kcal/mol): corilagin (−53.71 ± 5.05 kcal/mol), eriodictyol-7-rhamnopyranoside (−44.35 ± 4.51 kcal/mol), isoquercetin (−44.23 ± 2.92 kcal/mol), rutin (−42.15 ± 4.50 kcal/mol), and kaempferol-4-rhamnoside (−41.68 ± 3.69 kcal/mol). Wild-type EGFR simulations (PDB 1M17) yielded a selectivity index (IS) of 2.76 for corilagin (ΔΔG_bind_ = +34.22 kcal/mol), indicating T790M-preferential binding. Osimertinib reproduced its clinically established T790M selectivity under identical conditions (IS = 1.43; ΔΔG_bind_ = +7.74 kcal/mol), providing internal methodological validation. DFT at B3LYP-D3(BJ)/def2-TZVP established the quantum-mechanical basis for corilagin’s electrostatic affinity: its molecular electrostatic potential (MEP) surface minimum (V_s,min_ = −46.92 kcal/mol) directly predicts the largest MM-PBSA electrostatic term (ΔE_ele_ = −52.48 kcal/mol), establishing quantum-classical coherence. Supervised PCA/LDA of 7416 MM-PBSA trajectory frames identified solvation energy (ΔG_SOLV_) as the primary pharmacological class discriminant, with the first discriminant function (LD1) capturing 93.1% of inter-class binding variance. Collectively, corilagin (hydrolyzable tannin), eriodictyol-7-rhamnopyranoside (flavonoid glycoside), and phyltetralin (lignan) constitute diverse computational leads from *P. niruri* warranting experimental validation as T790M-directed agents in HCC.

## 1. Introduction

Hepatocellular carcinoma (HCC) ranks as the sixth most frequently diagnosed malignancy worldwide, with an estimated 905,677 new cases in 2022, and constitutes the third leading cause of cancer-related mortality globally [1]. The systemic treatment landscape remains narrow: sorafenib yields objective response rates of only 2–5% [2], while lenvatinib—the current first-line standard of care [3]—achieves a 24% objective response rate complicated by acquired resistance in approximately 30% of patients within six to eight months of treatment initiation [4,5,6]. This clinical trajectory leaves a substantial proportion of advanced HCC patients, particularly those with underlying cirrhosis, without effective systemic options at the time of disease progression.

Epidermal growth factor receptor (EGFR) signaling occupies a central position in hepatocarcinogenesis, with EGFR protein overexpression documented in 40–80% of human HCC tissues [7,8]. EGFR functions as a convergence hub integrating mitogenic signals through the PI3K/AKT/mTOR, RAS/MAPK, and STAT3 pathways, driving hepatocyte proliferation, epithelial-to-mesenchymal transition, and apoptotic resistance in HCC models and primary tumor specimens [8]. Despite this biological rationale, clinical trials of erlotinib and gefitinib monotherapy in HCC yielded suboptimal outcomes, attributed to primary resistance and the absence of validated predictive biomarkers [7]. These observations identify sustained EGFR signaling as a therapeutically tractable pathway of escape, particularly relevant in the post-lenvatinib resistance context.

Tantiwetrueangdet et al. identified 13 missense EGFR mutations in human HCC tissues spanning exons 18–23, establishing that EGFR mutational heterogeneity in liver cancer extends well beyond the canonical lung-cancer alteration landscape [9]. HCC-derived EGFR mutants are functioning, EGF-dependent, and erlotinib-resistant [9]. The mechanistic basis of this resistance is well characterized: the T790M gatekeeper substitution increases ATP-binding affinity approximately 10-fold, competitively displacing first-generation EGFR inhibitors from the kinase pocket [10]. These observations underscore the need for inhibitory strategies engaging EGFR T790M through mechanisms distinct from ATP-competitive displacement [11,12].

EGFR reactivation constitutes an established driver of acquired lenvatinib resistance in HCC. Jin et al. demonstrated that EGFR activation limits the lenvatinib response and that pharmacological EGFR inhibition restores drug sensitivity in preclinical HCC models [13]. Downstream resistance is further sustained through an EGFR-STAT3-ABCB1 signaling axis that promotes drug efflux in lenvatinib-resistant cells [14]. Genomic profiling of lenvatinib-resistant HCC cohorts identified EGFR amplification as a recurrent molecular alteration [15], and a prospective exploratory trial of lenvatinib plus gefitinib in EGFR-overexpressing lenvatinib-resistant HCC subsequently reported an objective response rate of 30.0% by mRECIST criteria [16]. These data position EGFR as a genomically validated and therapeutically actionable target in the lenvatinib-refractory HCC setting.

Clinical evidence further corroborates the therapeutic relevance of EGFR inhibition in HCC. A phase II trial of bevacizumab combined with erlotinib reported disease control in a subset of EGFR-expressing advanced HCC patients [17], and a case report of CTC-guided EGFR-targeted therapy in a patient with metastatic HCC refractory to standard systemic agents documented a confirmed partial response maintained for 14 months [18]. These data delineate a specific therapeutic gap: the absence of non-covalent binders capable of overcoming EGFR T790M-mediated resistance with a computationally predicted hepatoprotective pharmacological profile compatible with the cirrhotic background prevalent among HCC patients.

*Phyllanthus niruri* L. (*Phyllanthaceae*), documented in traditional medicine systems of Asia and Latin America for hepatic conditions and viral hepatitis [19,20,21], harbors more than 65 bioactive constituents including hydrolyzable tannins (corilagin, MW = 634.45 Da), flavonoid glycosides (rutin, eriodictyol-7-rhamnopyranoside), and lignans (phyltetralin, niranthin) [22,23,24]. Three independent lines of evidence support its evaluation as a source of computationally predicted leads against EGFR T790M in HCC: pharmacological EGFR inhibition reverses lenvatinib resistance in HCC preclinical models [13]; *P. niruri* dry extract induces apoptosis in human HCC cell lines [25]; and Phyllanthus species exhibit antitumoral activity in HCC-relevant in vitro systems [26]. These convergences identify P. niruri as a source of in silico predictions grounded in mechanistically coherent biological precedent rather than arbitrary phytochemical selection [27,28,29,30,31,32].

The crystal structure PDB 7A6K captures the EGFR T790M kinase domain in an inactive αC-helix-out conformation [33]. The co-present V948R substitution was deliberately introduced to prevent asymmetric receptor dimerization during crystallization, enabling high-resolution structural characterization of the monomeric inactive state required for inhibitor design [34]. Critically, V948R resides in the distal C-lobe of the kinase domain and does not interact with the ATP-binding cleft, the T790M gatekeeper position, or the pharmacologically relevant catalytic residues (Lys745, Met793, Cys797) that govern inhibitor binding [34]. This crystallographic construct has served as the structural basis for multiple peer-reviewed EGFR T790M inhibitor design studies from the Rauh laboratory [35,36], validating its suitability for computational investigation of T790M-directed non-covalent agents.

To address these scientific questions systematically, the present study employs an integrated multiscale computational framework structured as a hierarchy of analytical levels. Molecular docking against the EGFR T790M kinase domain (PDB 7A6K) established initial binding pose candidates from the *P. niruri* phytochemical library, providing entry-level affinity ranking [37]. Candidate compounds were subjected to 100-nanosecond molecular dynamics simulations, from which MM-PBSA binding free energies were derived to provide thermodynamically converged affinity estimates [38], and per-residue MM-GBSA energy decomposition at single-residue resolution identified the hot spot architecture governing selectivity. Supervised principal component and linear discriminant analyses (PCA/LDA) of the resulting MM-PBSA trajectory data then characterized the mechanistic basis of differential binding across chemical classes. Quantum-chemical analysis at the B3LYP-D3(BJ)/def2-TZVP level of density functional theory (DFT), encompassing molecular electrostatic potential (MEP) mapping, conceptual DFT descriptors, and non-covalent interaction (NCI) analysis, established the electronic-structure foundation for these in silico predictions [39]. Comparative 100-nanosecond molecular dynamics simulations against wild-type EGFR (PDB 1M17) provided quantitative mutant selectivity indices under identical simulation conditions [40].

The present study aims to identify, from the phytochemical repertoire of *Phyllanthus niruri* L., computationally predicted T790M-selective EGFR binders characterized through an integrated multiscale computational framework encompassing quantum mechanics, classical molecular dynamics, and multivariate chemometrics, with mutant selectivity rigorously quantified against wild-type EGFR under identical simulation conditions, thereby generating ranked computational leads that warrant experimental validation as non-covalent T790M-directed agents in hepatocellular carcinoma.

## 2. Results

### 2.1. Virtual Screening of Phyllanthus niruri Phytochemicals Against EGFR T790M

Thirty-seven *P. niruri* phytochemicals and two clinical standards were docked against EGFR T790M/V948R (PDB 7A6K) using a validated SwissDock protocol (TAK-788 redocking RMSD = 0.80 Å; DUD-E ROC-AUC = 0.91). Fourteen phytochemicals met the selection threshold of ΔG ≤ −7.91 kcal/mol (Supplementary Figure S1). The mean ΔG of selected compounds (−8.93 ± 0.28 kcal/mol) differed significantly from that of non-selected compounds (−6.43 ± 0.71 kcal/mol; Mann–Whitney U, *p* = 1.2 × 10^−5^; Supplementary Figure S2 and Table S1).

Among the 14 selected phytochemicals, rutin showed the most negative docking score (ΔG = −9.27 kcal/mol). Osimertinib and mobocertinib scored −9.74 and −9.31 kcal/mol, respectively. Five additional phytochemicals scored within 1.0 kcal/mol of mobocertinib: nirphyllin (−9.00), 2,3-desmethoxy seco-isolintetralin (−8.98), nirurin D(−8.97), isoquercitrin (−8.52), and hypophyllanthin (−8.35 kcal/mol; Supplementary Figure S2, Supplementary Table S1).

**Figure 1:**
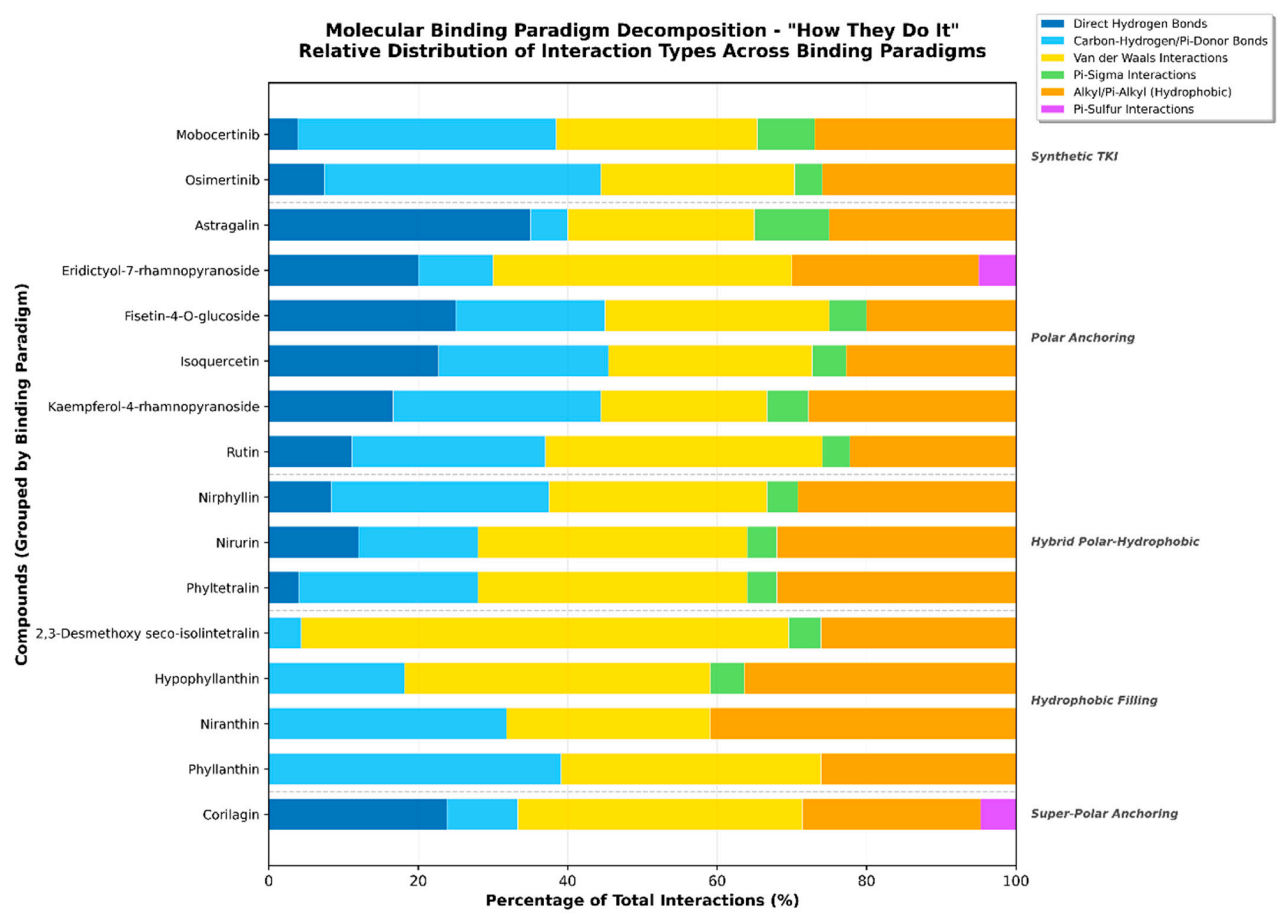
Residue-contact interaction profiles for 14 selected *P. niruri* phytochemicals and clinical reference compounds against EGFR T790M (PDB 7A6K). Stacked bar chart showing the relative frequency of hydrogen bonds, van der Waals, π-alkyl, π-sulfur, and cation-π interactions per compound (triplicate docking). Three interaction profiles are shown: (1) Corilagin: Hydrogen bond frequency > 40%, with additional van der Waals contacts. (2) Flavonoid glycosides (rutin, isoquercitrin, astragalin): Hydrogen bond frequency 25–35% with π-alkyl contacts; (3) Lignans (nirphyllin, hypophyllanthin, phyllanthin): Van der Waals contacts > 50% with π-alkyl interactions 20–30%. Osimertinib and mobocertinib profiles are included for reference. Bar segments scaled to 100% per compound.

Interaction profiling identified three residue-contact patterns (Figure 1). Flavonoid glycosides (rutin, isoquercitrin, astragalin) showed hydrogen-bonding with Met793 and Asp800, with conservation of water molecule HOH-611 (occupancy ≥ 75%), a pattern also observed for osimertinib (Supplementary Figure S3). Lignans (nirphyllin, hypophyllanthin, phyllanthin) showed predominant van der Waals contacts with Leu718, Val726, and Met790, with additional van der Waals contacts to Arg948; however, Arg948 arises from the V948R crystallographic stabilization mutation specific to PDB 7A6K and is not present in native EGFR T790M—these contacts are therefore construct-specific and should not be interpreted as pharmacologically relevant binding interactions (see Section 3.7). Corilagin showed a π-sulfur interaction with Cys797 and hydrogen-bonding contacts with Leu788, Arg841, and Asn842, including a double hydrogen bond with ASP855 (Figure 1). Contact frequencies are reported in Supplementary Table S2.

### 2.2. Multi-Platform Computational ADMET Profiling of 14 P. niruri Phytochemicals

Predicted ADMET profiles for the 14 selected phytochemicals and two clinical controls (osimertinib, mobocertinib) were assessed across three platforms (SwissADME, pkCSM, Deep-PK; Table 1, Supplementary Figures S4–S6). All values represent *in silico* predictions; experimental pharmacokinetic validation was not performed.

Eight of 14 phytochemicals (57%) showed predicted CYP3A4 inhibition confidence <0.10, including corilagin, nirurin, rutin, isoquercitrin, astragalin, kaempferol-4′-rhamnopyranoside, eriodictyol-7-rhamnopyranoside, and fisetin-4-O-glucoside. Osimertinib and mobocertinib showed CYP3A4 inhibition confidence >0.63 (Table 1). Lignans showed intermediate CYP3A4 inhibition (symbol ±). Glycosides and polyphenols showed predicted UGT-centered Phase II metabolism (UGT1A1, UGT1A9, UGT1A10), whereas lignans showed predicted CYP-distributed metabolism (CYP2C8, CYP2C9, CYP2C19; Table 1, Supplementary Table S2).

hERG blocker confidence was >0.99 for both clinical controls. Eight phytochemicals (astragalin, corilagin, hypophyllanthin, isoquercitrin, kaempferol-4′-rhamnopyranoside, niranthin, nirphyllin, nirurin) showed hERG confidence <0.10. Phyllanthin and eriodictyol-7-rhamnopyranoside showed values in the high predicted-risk category (symbol −; Table 1, Supplementary Figures S4–S6).

DILI II confidence for osimertinib was 0.58–0.67. Corilagin and fisetin-4-O-glucoside showed high predicted DILI II confidence (symbol −); all other glycosides and polyphenols showed low predicted risk (+). Among lignans, hypophyllanthin, nirphyllin, and phyllanthin showed low predicted risk (+); niranthin and 2,3-desmethoxy seco-isolintetralin showed mixed predictions (±; Table 1).

Six lignans showed predicted high Caco-2 permeability (log Papp 0.98–1.14 cm/s). Glycosides and polyphenols showed predicted low-to-very-low permeability (log Papp −1.34 to 0.31). Phyltetralin and hypophyllanthin showed LogBB > 0.2; osimertinib showed LogBB = −0.045 (Table 1).

Osimertinib and mobocertinib showed P-glycoprotein (P-gp) substrate confidence > 0.75. Among phytochemicals, glycosides, polyphenols, hypophyllanthin, and nirphyllin showed P-gp substrate classification (+); remaining lignans showed mixed predictions (Table 1).

Based on predicted Caco-2 permeability and primary metabolic pathway, the phytochemicals separated into two groups: (i) lignans with high permeability and CYP-distributed metabolism; (ii) glycosides and polyphenols with low permeability, UGT-centered metabolism, and low predicted CYP3A4 inhibition (Supplementary Table S2, Supplementary Figures S4–S6). The pharmacokinetic implications are discussed in Section 3.

### 2.3. PASS and KinScreen Predictions Suggest Distinct Polypharmacological Profiles and High Hepatoprotective Probability Scores Across P. niruri Phytochemicals

PASS Online (ChEMBL v.21) and KinScreen (442 human kinases) predictions are probabilistic estimates (Pa = probability of activity) and do not constitute experimental confirmation. All values are in silico predictions.

Hepatoprotective Pa ≥ 0.940 was predicted for nirurin (0.974), rutin (0.968), isoquercitrin (0.961), and astragalin (0.940). Antineoplastic Pa for these compounds ranged from 0.832 to 0.849. Osimertinib and mobocertinib showed antineoplastic Pa of 0.797 and 0.682, respectively, and hepatoprotective Pa < 0.30 (Supplementary Figure S7, Supplementary Table S3). Twelve of 14 phytochemicals showed predicted carbonic anhydrase (CA-III, CA-IX, CA-XII) Pa ≥ 0.40 (Supplementary Table S4); clinical controls showed Pa < 0.30 for all three CA isoforms.

**Figure 2:**
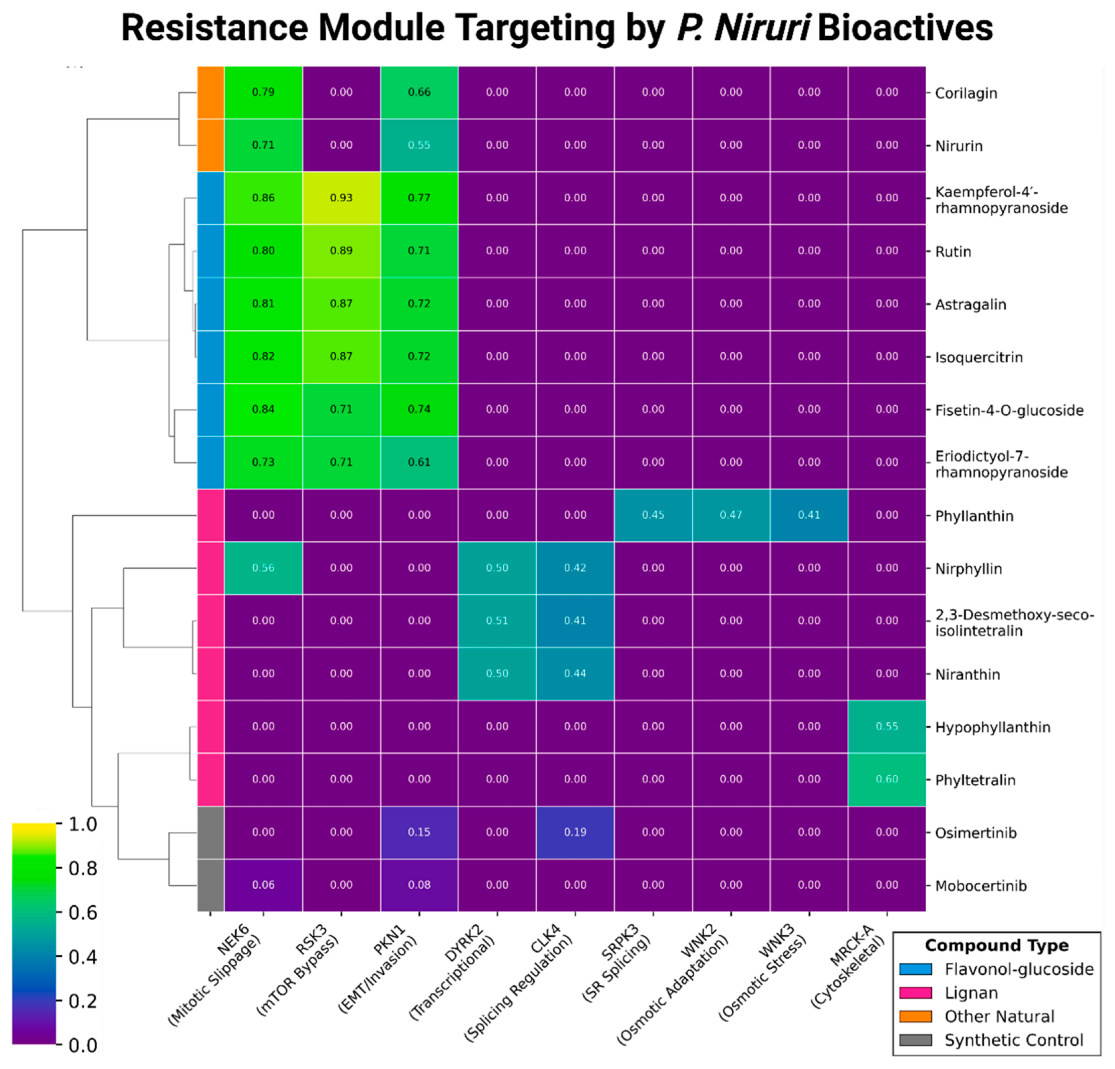
**KinScreen-predicted kinase interaction profiles (Pa) for 14 *P. niruri* phytochemicals and clinical reference compounds across 442 human kinases.** Unsupervised hierarchical clustering of Pa values (Pa ≥ 0.40 threshold). Rows: compounds; columns: kinase targets with Pa ≥ 0.40 in at least one compound. Color scale: Pa from 0.0 (purple) to 1.0 (yellow). Three compound clusters are observed: flavonoid glycosides (NEK6 Pa 0.71–0.86; RSK3, PKN1), lignans (DYRK2 Pa 0.40–0.51; CLK4 0.37–0.44), and a phyltetralin/hypophyllanthin sub-cluster (MRCK-A Pa > 0.55). Clinical controls showed Pa < 0.30 for NEK6, DYRK2, CLK4, and MRCK-A (osimertinib CLK4 Pa = 0.19; PKN1 Pa = 0.15). All predictions are *in silico*; experimental kinase assays were not performed.

The mean number of predicted high-confidence kinase interactions (Pa ≥ 0.40) was 3.5 ± 2.1 for phytochemicals vs. 28.0 ± 12.0 for clinical controls. Flavonoid glycosides showed predicted NEK6 interactions (Pa = 0.71–0.86); lignans showed predicted DYRK2 (0.40–0.51) and CLK4 (0.37–0.44) interactions; phyltetralin and hypophyllanthin showed predicted MRCK-A interactions (Pa > 0.55). Osimertinib showed CLK4 Pa = 0.19 and PKN1 Pa = 0.15 (Figure 2, Supplementary Table S4).

Collectively, the phytochemicals showed computationally predicted interactions spanning EGFR T790M docking (Section 2.1), kinase targets, P-glycoprotein modulation (Pa 0.60–0.87), CYP3A4 non-inhibition for 8/14 compounds (Section 2.2), and CA-IX/CA-XII targeting for 12/14 compounds.

### 2.4. Molecular Dynamics Simulations Reveal Class-Dependent Structural Stability, Interaction Networks, and MM-PBSA Binding Free Energies for Selected P. niruri Phytochemicals Against EGFR T790M

#### 2.4.1. Conformational Stability over 100 ns Trajectories

**Figure 3:**
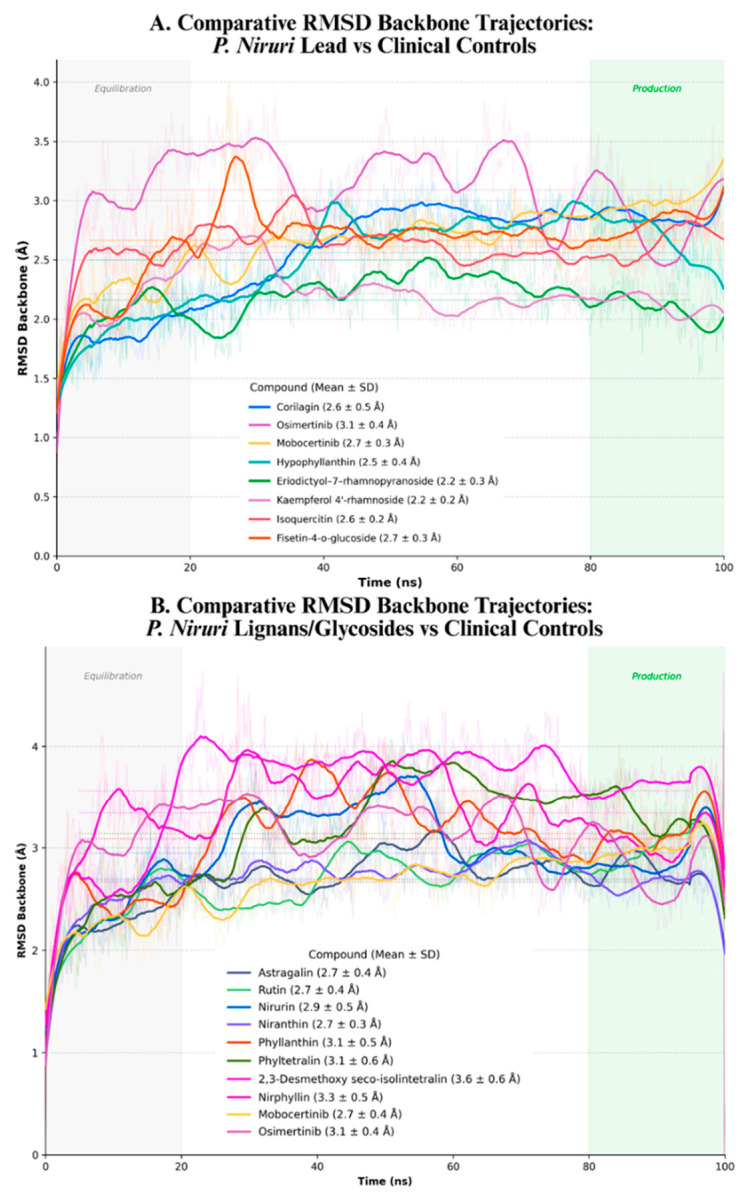
**Backbone Cα RMSD trajectories for EGFR T790M/V948R complexes over 100 ns MD simulations.** Solid lines: Savitzky–Golay smoothed; shaded bands: ±1 SD. Gray shading: equilibration phase (0–20 ns); green shading: production phase (80–100 ns). (**A**) Lead compounds and controls: eriodictyol-7-rhamnopyranoside (μ = 2.16 ± 0.25 Å, τ = 3.54 ns), kaempferol-4′-rhamnoside (μ = 2.21 ± 0.24 Å, τ = 4.32 ns), corilagin (μ = 2.56 ± 0.45 Å, τ = 13.77 ns), isoquercitrin (μ = 2.61 ± 0.25 Å), mobocertinib (μ = 2.66 ± 0.34 Å, τ = 9.96 ns), osimertinib (μ = 3.09 ± 0.41 Å). (**B**) Secondary compounds: astragalin (μ = 2.67 ± 0.33 Å), rutin (μ = 2.70 ± 0.37 Å), nirurin (μ = 2.95 ± 0.47 Å), phyllanthin (μ = 3.10 ± 0.50 Å), phyltetralin (μ = 3.14 ± 0.56 Å, τ = 11.26 ns), nirphyllin (μ = 3.35 ± 0.47 Å), 2,3-desmethoxy seco-isolintetralin (μ = 3.57 ± 0.53 Å), niranthin (μ = 2.69 ± 0.30 Å). Complete metrics in Supplementary Table S5.

Backbone Cα RMSD and ligand heavy-atom RMSD were monitored over 100 ns NPT trajectories for 14 phytochemicals and two clinical controls against EGFR T790M/V948R (Figure 3, Supplementary Figures S10 and S11, Supplementary Tables S5 and S6). Osimertinib and mobocertinib showed mean backbone RMSD of 3.09 ± 0.41 Å (τ = 2.82 ns) and 2.66 ± 0.34 Å (τ = 9.96 ns), respectively; ligand RMSD was 5.37 ± 0.64 Å (1.3% stable frames) and 5.45 ± 0.55 Å (0.1% stable frames; Figure 3A, Supplementary Figure S10A).

Among glycosides, eriodictyol-7-rhamnopyranoside and kaempferol-4′-rhamnoside showed the lowest backbone RMSD (μ = 2.16 ± 0.25 and 2.21 ± 0.24 Å; τ = 3.54 and 4.32 ns) and ligand RMSD of 2.31 ± 0.20 Å (100% stable) and 2.47 ± 0.29 Å (96.6% stable), respectively. Isoquercitrin showed the lowest ligand RMSD in the dataset (1.78 ± 0.30 Å; 100% stable). Secondary glycosides (astragalin, rutin, nirurin) showed intermediate backbone RMSD values (2.67–2.95 Å; Figure 3B, Supplementary Table S5). Corilagin showed backbone RMSD μ = 2.56 ± 0.45 Å with τ = 13.77 ns (Figure 3A).

Among lignans, four of six compounds showed backbone RMSD > 3.0 Å: phyllanthin (3.10 ± 0.50 Å), phyltetralin (3.14 ± 0.56 Å, τ = 11.26 ns), nirphyllin (3.35 ± 0.47 Å), and 2,3-desmethoxy seco-isolintetralin (3.57 ± 0.53 Å; Figure 3B, Supplementary Table S5). Ligand RMSD for niranthin and phyllanthin was 7.48 ± 1.32 Å and 5.67 ± 1.33 Å, respectively (Supplementary Figure S10B, Supplementary Table S6). Phyltetralin showed the lowest lignan ligand RMSD (2.18 ± 0.47 Å; 92.6% stable). Fisetin-4-O-glucoside underwent a conformational transition at ~25 ns (RMSD ≈ 2.5 Å → >6.5 Å; Supplementary Figure S10A).

Mean radius of gyration (Rg) for glycoside-bound complexes ranged from 1.976 to 1.987 nm (σ = 0.010–0.022 nm); clinical controls showed mean Rg = 1.996–1.999 nm (Supplementary Figure S11A, Supplementary Table S7). Corilagin-bound complex showed mean Rg = 1.994 nm with low conformational dispersion. Nirphyllin-bound complex showed the highest mean Rg (2.042 nm, Range = 0.141 nm; Supplementary Figure S11B, Supplementary Table S7). Mean solvent-accessible surface area (SASA) for kaempferol-4′-rhamnoside-bound complex was 15,151 ± 266 Å^2^; isoquercitrin 15,237 ± 356 Å^2^; rutin 15,240 ± 435 Å^2^ (Supplementary Figure S12A, Supplementary Table S8). Osimertinib-bound complex showed mean SASA = 15,599 ± 370 Å^2^. Nirphyllin-bound complex showed mean SASA = 15,950 ± 341 Å^2^ (Range = 2578 Å^2^; Supplementary Figure S12B, Supplementary Table S8).

#### 2.4.2. Intermolecular Hydrogen-Bond Occupancy and Per-Residue RMSF

**Figure 4:**
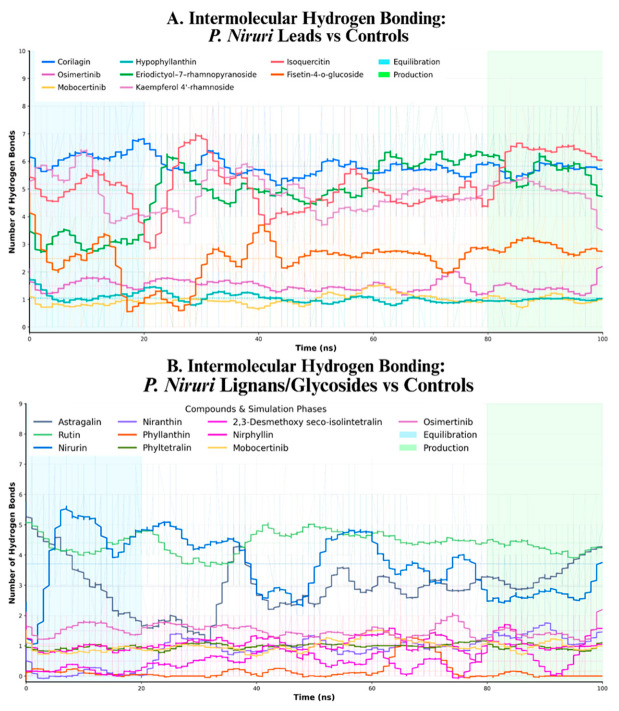
**Intermolecular hydrogen-bond counts between each ligand and EGFR T790M/V948R over 100 ns simulations.** Shaded bands: ±1 SD. (**A**) Lead compounds and controls: corilagin (mean = 5.83 ± 1.16; maximum 9; residence time 33.30 ns), isoquercitrin (5.18 ± 1.40; maximum 9; residence time > 100 ns), eriodictyol-7-rhamnopyranoside (4.95 ± 1.52; maximum 8; residence time > 100 ns), kaempferol-4′-rhamnoside (4.84 ± 1.10; maximum 9; residence time > 100 ns), fisetin-4-O-glucoside (intermediate occupancy), osimertinib (1.49 ± 0.61; maximum 3; residence time 3.60 ns), mobocertinib (0.98 ± 0.51). (**B**) Secondary compounds: phyltetralin (0.98 ± 0.39; maximum 2), hypophyllanthin (1.05 ± 0.46; maximum 3), phyllanthin (0.16 ± 0.42; residence time 0.27 ns), nirphyllin (1.05 ± 0.73; residence time 0.51 ns), 2,3-desmethoxy seco-isolintetralin (0.49 ± 0.57). Complete metrics in Supplementary Table S9.

Intermolecular hydrogen-bond counts were computed over the 100 ns production trajectory (Figure 4, Supplementary Table S9). Corilagin showed mean H-bonds = 5.83 ± 1.16 (maximum 9; residence time 33.30 ns). Isoquercitrin, eriodictyol-7-rhamnopyranoside, and kaempferol-4′-rhamnoside showed mean H-bonds of 5.18 ± 1.40, 4.95 ± 1.52, and 4.84 ± 1.10, respectively (maxima 8–9; residence times > 100 ns; Figure 4A). Osimertinib showed mean H-bonds = 1.49 ± 0.61 (maximum 3; residence time 3.60 ns); mobocertinib = 0.98 ± 0.51 bonds (maximum 3; residence time 0.86 ns; Figure 4A,B). Phyltetralin and hypophyllanthin showed mean H-bonds of 0.98 ± 0.39 and 1.05 ± 0.46, respectively. Phyllanthin showed 0.16 ± 0.42 bonds (residence time 0.27 ns); nirphyllin = 1.05 ± 0.73 bonds (residence time 0.51 ns; Figure 4B, Supplementary Table S9).

**Figure 5:**
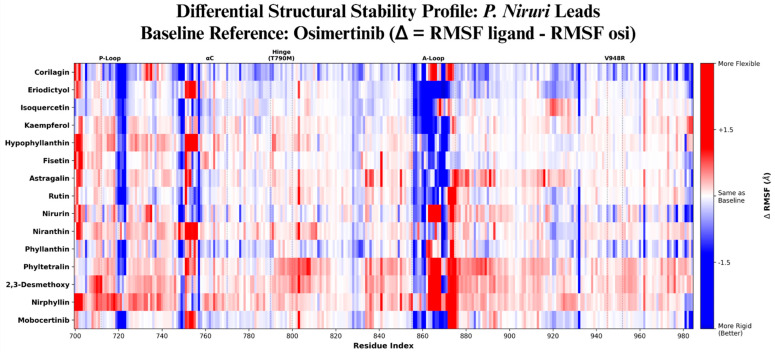
Per-residue differential RMSF heatmap (ΔRMSF = RMSF_compound_ − RMSF_osimertinib_) for 16 compounds against EGFR T790M/V948R over 100 ns trajectories. Color scale: −1.5 Å (blue, reduced flexibility) to +1.5 Å (red, increased flexibility). Compounds ordered by decreasing MM-PBSA affinity (Supplementary Table S11). Dashed vertical lines demarcate P-Loop, G-Loop, Hinge/DFG motif, and Activation Loop. Corilagin: global ΔRMSF = −0.212 Å; ΔRMSF < −0.10 Å at P-Loop (750–757), Hinge/T790M (874–876), and A-Loop (855–875). Eriodictyol-7-rhamnopyranoside: ΔRMSF = −0.117 Å (residues 834–837, 861–877). Isoquercitrin: ΔRMSF = −0.030 Å; kaempferol-4′-rhamnoside: reductions at 750–757 and 862–876. Nirphyllin: mean RMSF = 1.865 Å, ΔRMSF = +0.429 Å; 2,3-desmethoxy seco-isolintetralin: ΔRMSF = +0.347 Å; phyltetralin: ΔRMSF = +0.328 Å. Complete per-residue values in Supplementary Table S10.

Per-residue ΔRMSF =RMSF_compound_ − RMSF_osimertinib_) was computed over the 100 ns trajectory (Figure 5, Supplementary Figure S13, Supplementary Table S10). Corilagin showed global ΔRMSF = −0.212 Å, with ΔRMSF < −0.05 Å at P-loop residues 750–757, and ΔRMSF < −0.10 Å at hinge/ATP-binding residues 874–876 and 887–891. Eriodictyol-7-rhamnopyranoside showed ΔRMSF = −0.117 Å with reductions at residues 834–837 and 861–877. Isoquercitrin showed ΔRMSF = −0.030 Å; kaempferol-4′-rhamnoside showed reductions at residues 750–757 and 862–876. Nirphyllin showed mean RMSF = 1.865 Å (ΔRMSF = +0.429 Å), 2,3-desmethoxy seco-isolintetralin ΔRMSF = +0.347 Å, and phyltetralin ΔRMSF = +0.328 Å (Figure 5, Supplementary Table S10). The ΔRMSF heatmap (Figure 5) shows negative ΔRMSF concentrated at P-Loop, Hinge/T790M, and A-Loop for corilagin and eriodictyol-7-rhamnopyranoside, and positive ΔRMSF distributed across all kinase domains for nirphyllin, 2,3-desmethoxy seco-isolintetralin, and phyltetralin.

#### 2.4.3. MM-PBSA Binding Free Energies for EGFR T790M

**Figure 6:**
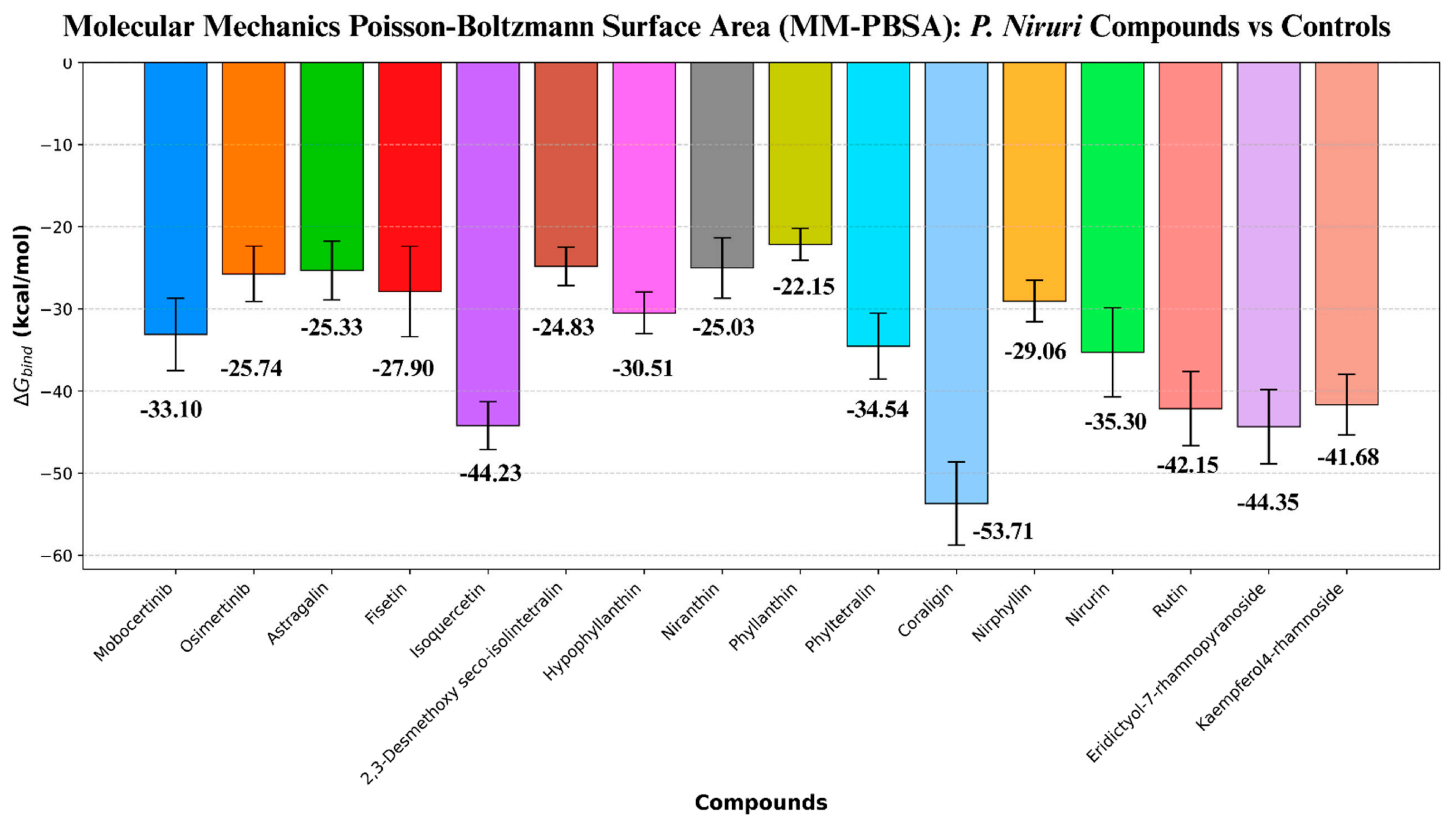
MM-PBSA binding free energies (ΔG_bind_, kcal/mol) for 16 *Phyllanthus niruri* phytochemicals and two covalent reference inhibitors against EGFR T790M/V948R (PDB 7A6K; T = 310 K, P = 1 atm, NPT ensemble, 201 production frames per system, 50–100 ns window). Error bars represent ±1 standard deviation from MM-PBSA ensemble averaging. The vertical dashed reference line indicates the non-covalent binding component of osimertinib (ΔG_bind_ = −25.74 ± 3.37 kcal/mol); compounds to the left of this line show less favorable predicted binding relative to this reference. Five phytochemicals exceeded the osimertinib non-covalent reference: corilagin (−53.71 ± 5.05 kcal/mol), eriodictyol-7-rhamnopyranoside (−44.35 ± 4.51 kcal/mol), rutin (−−42.15 ± 4.50 kcal/mol), isoquercetin (−44.23 ± 2.92 kcal/mol), and kaempferol-4-rhamnoside (−41.68 ± 3.69 kcal/mol). Osimertinib and mobocertinib form irreversible covalent bonds with Cys797 via Michael addition; the ΔG_bind_ values reported here (−25.74 ± 3.37 and −−33.10 ± 4.41 kcal/mol, respectively) reflect the non-covalent binding component only and are not directly comparable with the phytochemical affinities. Complete per-compound statistics, convergence diagnostics, and pairwise Kruskal–Wallis comparisons are provided in Supplementary Table S11.

MM-PBSA binding free energies were computed over the 100 ns production trajectory at T = 310 K, P = 1 atm, NPT ensemble (Figure 6, Supplementary Table S11). The non-covalent binding component of osimertinib (ΔG_bind_ = −25.74 ± 3.37 kcal/mol) was used as the internal reference value for all pairwise comparisons; this value reflects exclusively the non-covalent contribution, as the irreversible Michael addition to Cys797 that governs osimertinib’s clinical potency is not captured by the standard MM-PBSA formalism (see Section 3.6).

Five phytochemicals showed predicted binding affinities more negative than the osimertinib non-covalent reference. Corilagin showed the most favorable ΔG_bind_ = −53.71 ± 5.05 kcal/mol (ΔΔG relative to osimertinib: −27.97 kcal/mol; complete pairwise statistics in Supplementary Table S11). Eriodictyol-7-rhamnopyranoside showed ΔG_bind_ = −44.35 ± 4.51 kcal/mol (ΔΔG = −18.61 kcal/mol). Isoquercetin showed ΔG_bind_ = −44.23 ± 2.92 kcal/mol (ΔΔG = −18.49 kcal/mol). Rutin showed ΔG_bind_ = −42.15 ± 4.50 kcal/mol (ΔΔG = −16.41 kcal/mol). Kaempferol-4′-rhamnoside showed ΔG_bind_ = −41.68 ± 3.69 kcal/mol (ΔΔG = −15.94 kcal/mol).

Additional phytochemicals showed ΔG_bind_ values distributed across the following range: nirurin −35.30 ± 5.42 kcal/mol, phyltetralin −34.54 ± 4.01 kcal/mol, hypophyllanthin −30.51 ± 2.54 kcal/mol, nirphyllin −29.06 ± 2.52 kcal/mol, fisetin −27.90 ± 3.18 kcal/mol, astragalin −25.33 ± 2.71 kcal/mol, 2,3-desmethoxy seco-isolintetralin −24.83 ± 2.34 kcal/mol, niranthin −25.03 ± 2.89 kcal/mol, and phyllanthin −22.15 ± 1.94 kcal/mol (Figure 6, Supplementary Table S11). Phyllanthin showed a ΔG_bind_ value less negative than the osimertinib non-covalent reference (ΔΔG = +3.59 kcal/mol; Supplementary Table S11). Complete pairwise Kruskal–Wallis tests with Benjamini–Hochberg correction and Cohen’s d effect sizes for all inter-compound comparisons are reported in Supplementary Table S11.

Mobocertinib yielded ΔG_bind_ = −33.10 ± 4.41 kcal/mol, reflecting solely its non-covalent binding component. As with osimertinib, mobocertinib forms an irreversible covalent bond with Cys797 via Michael addition; its clinical potency is governed by this covalent mechanism, which is not captured by standard MM-PBSA non-covalent calculations. The reported value should not be interpreted as its total binding affinity, and direct comparison with the phytochemical ΔG_bind_ values is methodologically inappropriate (see Section 3.6).

### 2.5. Comparative Docking and Molecular Dynamics of T790M Versus Wild-Type EGFR Quantify Predicted Mutant Selectivity

#### 2.5.1. Redocking Protocol Validation and Comparative Docking Scores

Redocking of co-crystallized ligands validated the SwissDock 2024 protocol independently for both receptor forms. Erlotinib redocked into wild-type EGFR (PDB: 1M17) with an RMSD of 1.8315 Å relative to its crystallographic pose; TAK-788 (mobocertinib) redocked into the T790M/V948R mutant (PDB: 7A6K) with an RMSD of 0.80 Å (Supplementary Table S12). Both values fell below the standard 2.0 Å acceptance threshold, confirming protocol integrity across the two conformational states of the kinase domain (C-helix-out inactive in T790M; C-helix-in active in WT). The T790M/V948R model harbors an additional V948R substitution in the C-lobe; this residue is distal to the ATP-binding site and does not contact the binding pocket, and therefore is not expected to systematically influence docking results.

Comparative docking scores across the T790M and WT models are summarized in Table 2. Osimertinib scored −9.737 kcal/mol (T790M) and −8.0 kcal/mol (WT), a difference (ΔΔG) of +1.74 kcal/mol in favor of the mutant form. Among the phytochemicals, corilagin scored −8.677 kcal/mol (T790M) and −8.4 kcal/mol (WT) (ΔΔG = +0.28 kcal/mol). Phyltetralin scored −8.507 kcal/mol (T790M) and −8.0 kcal/mol (WT) (ΔΔG = +0.51 kcal/mol). Eriodictyol-7-rhamnopyranoside scored −8.561 kcal/mol (T790M) and −9.7 kcal/mol (WT) (ΔΔG = −1.14 kcal/mol). This apparent WT preference at the static docking level is inverted by the dynamic MM-PBSA analysis, which captures the polar desolvation penalty imposed on this glycosylated flavonoid in explicit solvent (Section 2.5.3).

#### 2.5.2. MD Stability Classification, Catalytic Residue Rigidification, and Hydrogen-Bond Occupancy in Wild-Type EGFR Simulations

**Figure 7:**
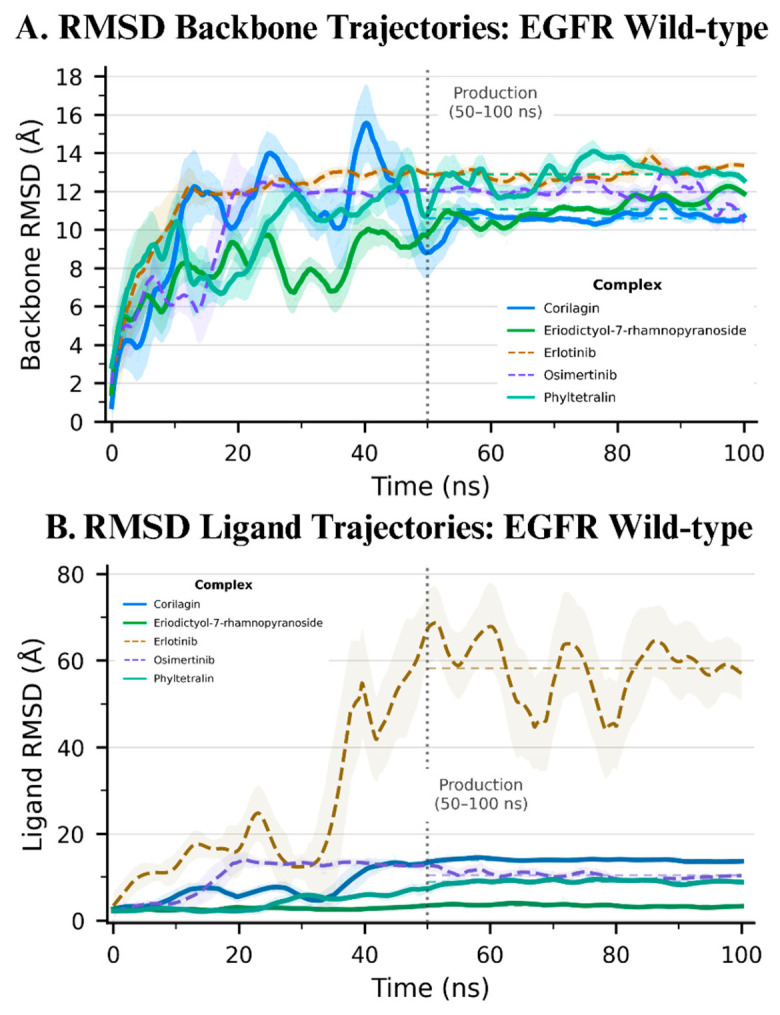
**Root-mean-square deviation (RMSD) of the EGFR wild-type (PDB 1M17) kinase domain and bound ligands during 100 ns molecular dynamics simulations.** (**A**) Backbone Cα RMSD of the EGFR WT kinase domain (residues 696–1022) for five complexes, calculated with respect to the energy-minimized crystallographic reference structure. (**B**) Heavy-atom RMSD of each ligand calculated relative to its initial docking pose within the ATP-binding site. Thick curves represent Savitzky–Golay smoothed trajectories (window = 501 frames, polynomial order = 3). Shaded bands indicate ±1 rolling standard deviation (500-frame sliding window). Semi-transparent points represent raw per-frame RMSD values. The vertical dashed line at *t* = 50 ns marks the onset of the MM-PBSA analysis window (frames 50–100 ns; 201 frames). The vertical dashed line at *t* = 25 ns denotes the end of the equilibration-to-production transition phase.

Extended 100 ns MD simulations of each ligand–WT EGFR complex were conducted to assess dynamic stability. Backbone Cα RMSD values below 4.0 Å are considered indicative of structural equilibrium; ligand RMSD values below 3.0 Å indicate confinement within the binding site (Supplementary Table S13). Backbone RMSD trajectories for the WT complexes (Figure 7A) demonstrated stabilization during the final 25 ns, with mean values ranging from 9.506 ± 1.993 Å (eriodictyol-7-O-rhamnoside) to 12.197 ± 1.824 Å (erlotinib).

Ligand RMSD profiles (Figure 7B; Supplementary Table S13) classified WT-binding behavior into three stability categories. Class I (mean RMSD < 5 Å) comprised only eriodictyol-7-O-rhamnoside (3.075 ± 0.580 Å, maximum 5.332 Å), indicative of sustained binding-site occupancy throughout the 100 ns trajectory. Class II (mean RMSD 5–15 Å) encompassed osimertinib (10.324 ± 3.119 Å), corilagin (10.673 ± 4.069 Å), and phyltetralin (6.485 ± 2.835 Å), consistent with partial or fluctuating binding engagement. Class III (mean RMSD > 15 Å) comprised erlotinib alone (41.574 ± 22.278 Å, maximum 84.542 Å), which underwent complete dissociation from the WT binding site during the simulation.

Per-residue RMSF analysis of key catalytic residues confirmed markedly distinct conformational fluctuation profiles between the Class I and Class II/III binders. In the corilagin–WT complex, elevated fluctuation was observed at THR790 (4.753 Å), LYS745 (5.029 Å), MET793 (5.769 Å), and CYS797 (5.710 Å). By contrast, eriodictyol-7-O-rhamnoside suppressed fluctuation at these same residues to 1.836, 1.999, 1.942, and 2.032 Å, respectively—values comparable to those of the osimertinib control (1.889, 1.983, 2.191, and 1.903 Å).

Hydrogen-bond occupancy analysis over the 100 ns WT trajectories is summarized in Supplementary Table S13 and Supplementary Figure S14. Eriodictyol-7-O-rhamnoside formed a persistent hydrogen-bond network throughout the simulation (mean 3.20 ± 1.03 per frame; maximum six simultaneous bonds), achieving ≥1 hydrogen bond in 100% of frames and ≥3 bonds in 71.9% of frames. Corilagin maintained a comparable frame-level occupancy (mean 2.64 ± 1.03; ≥1 bond in 96.4% of frames) despite higher ligand-RMSD excursions, confirming that transient rebinding events sustained intermittent polar contacts. Osimertinib yielded reduced occupancy (mean 0.94 ± 0.85; zero bonds in 33.9% of frames). Phyltetralin exhibited progressive temporal decline from 1.32 bonds per frame (0–25 ns) to 0.24 bonds per frame (75–100 ns). Erlotinib was essentially hydrogen-bond-inactive (mean 0.10 ± 0.38; zero bonds in 92.4% of frames), consistent with its Class III dissociation profile.

#### 2.5.3. Molecular Dynamics Stability Classification Identifies Eriodictyol-7-rhamnoside as the Sole Class I Binder in the Wild-Type Simulation

**Figure 8:**
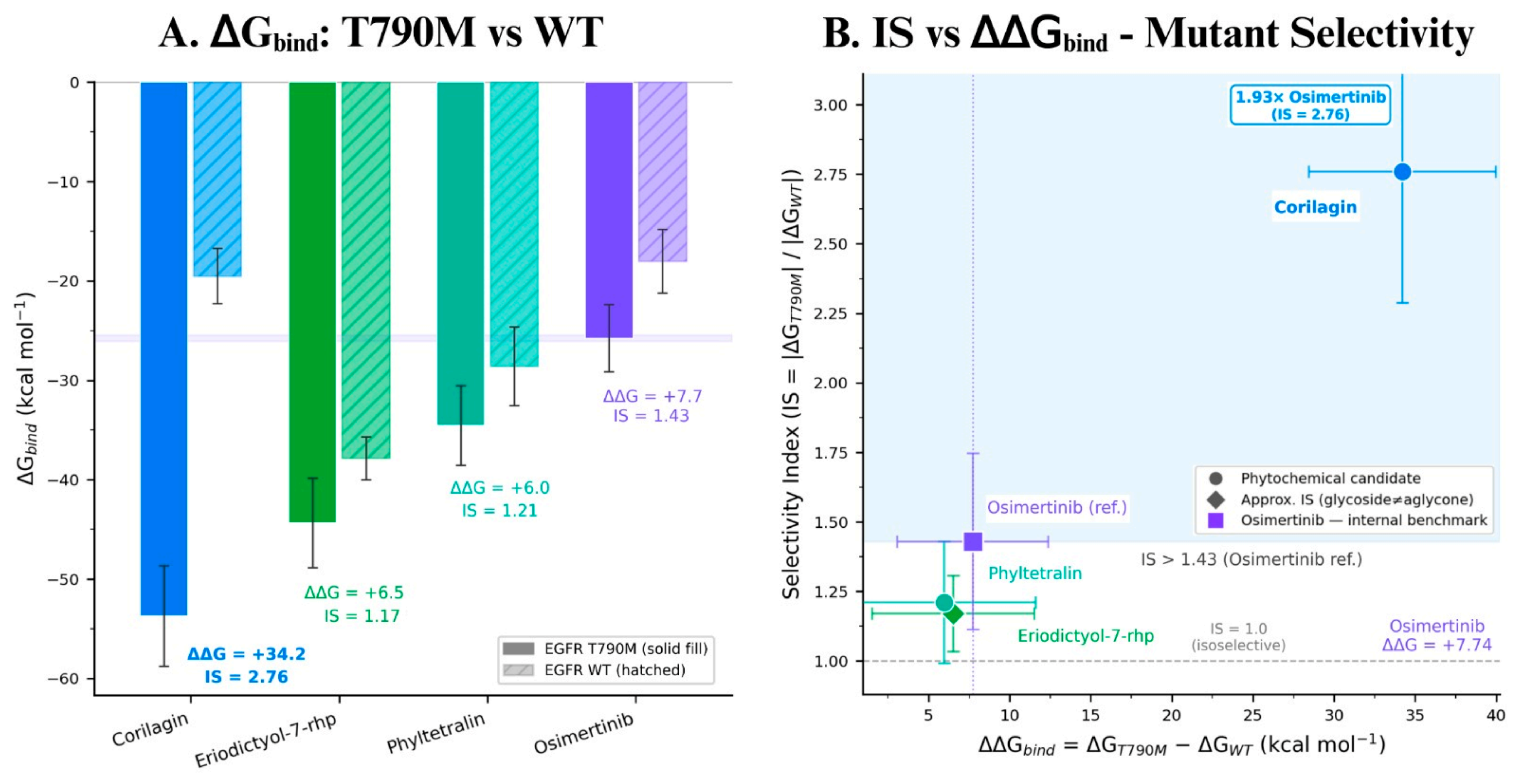
Comparative MM-PBSA binding free energy analysis and mutant-selectivity profiling of *Phyllanthus niruri* phytochemicals and reference inhibitors against EGFR T790M (PDB 7A6K/V948R) and wild-type EGFR (PDB 1M17). (**A**) Grouped bar chart depicting Δ*G*bind (kcal mol^−1^) for each compound in the T790M mutant (solid fill) and WT (hatched fill) contexts. Error bars represent ±1 standard deviation from MM-PBSA ensemble averaging (201 frames, 50–100 ns production window). The horizontal reference band (dashed purple) indicates the Osimertinib T790M Δ*G*bind benchmark (−25.74 ± 3.37 kcal mol^−1^). (**B**) Two-dimensional selectivity map plotting the Selectivity Index (IS = |Δ*G*T790M|/|Δ*G*WT|) against ΔΔ*G*bind (=Δ*G*T790M − Δ*G*WT, kcal mol^−1^). Error bars represent propagated uncertainties. The horizontal dashed line at IS = 1.0 defines the isoselective threshold; the horizontal dashed line at IS = 1.43 corresponds to the Osimertinib internal benchmark. The vertical dashed line at ΔΔ*G* = 7.74 kcal mol^−1^ marks the Osimertinib ΔΔ*G* reference. Compounds in the upper-right quadrant exhibit both superior mutant affinity and superior selectivity relative to the clinical T790M benchmark. Circle markers (●) denote *P. niruri* phytochemical candidates; the diamond marker (◆) denotes the approximate IS for Eriodictiyol-7-rhamnopyranoside (T790M) compared to the WT.

MM-PBSA-estimated total binding free energies for the WT EGFR complexes, together with their thermodynamic component decomposition, are presented in Supplementary Table S15 and Figure 8. Eriodictyol-7-rhamnoside recorded the most favorable WT ΔGbind of −37.83 ± 2.16 kcal/mol, driven by balanced van der Waals (ΔE_vdW_ = −40.85 ± 0.28 kcal/mol) and electrostatic (ΔEEL = −37.69 ± 1.66 kcal/mol) contributions, partially offset by the desolvation penalty (ΔEGB = +47.25 ± 1.18 kcal/mol). Phyltetralin ranked second (−28.57 ± 3.97 kcal/mol), dominated by strong van der Waals interactions (ΔE_vdW_ = −41.91 ± 1.78 kcal/mol). Corilagin yielded −19.49 ± 2.79 kcal/mol, featuring a notably large electrostatic gas-phase term (ΔEEL = −52.48 ± 1.04 kcal/mol) substantially counteracted by the desolvation penalty (ΔEGB = +67.24 ± 1.72 kcal/mol). Osimertinib registered −18.00 ± 3.21 kcal/mol, within 1.5 kcal/mol of corilagin. Erlotinib produced a statistically non-significant estimate of −2.71 ± 3.05 kcal/mol (95% CI overlapping zero), consistent with the observed dissociation event; the covalent mechanism of erlotinib at CYS797 is not captured by non-covalent MM-PBSA formalism, and this estimate should not be interpreted as a meaningful binding affinity.

MM-PBSA-derived mutant-selective ΔΔG_bind_ values and the *in silico*-estimated selectivity index (IS = ΔG_bind_ T790M/ΔG_bind_ WT) are reported in Table 3. The osimertinib reference control yielded IS = 1.43 (ΔΔG_bind_ = +7.74 kcal/mol). The computed IS of 2.76 for corilagin (ΔΔG_bind_ = +34.22 kcal/mol) exceeds the osimertinib reference (IS = 1.43) by a factor of 1.93, representing the largest MM-PBSA-derived mutant selectivity estimate in the present dataset. Phyltetralin reached IS = 1.21 (ΔΔG_bind_ = +5.97 kcal/mol). Eriodictiyol-7-rhamnopyranoside attained an approximate IS of 1.17 (ΔΔG_bind_ ≈ +6.52 kcal/mol). It is emphasized that these *in silico* estimated selectivity indices do not constitute experimental validation of mutant selectivity and require confirmation in appropriate cellular models.

### 2.6. Multivariate PCA and LDA of MM-PBSA Energy Component Profiles Across 16 P. niruri Phytochemicals Against EGFR T790M

#### 2.6.1. Dataset and Rationale

Principal component analysis (PCA) and linear discriminant analysis (LDA) were applied to the full MM-PBSA energy component profiles of all 16 compounds evaluated against EGFR T790M/V948R (PDB 7A6K). The dataset comprised 7416 MD trajectory frames sampled at 100 ps intervals, organized into four pharmacological classes: flavonoids (Astragalin, Eriodictyol-7-rhamnopyranoside, Fisetin, Isoquercetin, Kaempferol-4-rhamnoside, Rutin; *n* = 1200 frames), lignans (12-Desmethoxywuweizisu C, Hypophyllanthin, Niranthin, Phyllanthin, Phyltetralin; *n* = 1000), secondary metabolites (Corilagin, Nirphyllin, Nirurin; *n* = 600), and covalent inhibitor controls (Mobocertinib, Osimertinib; *n* = 302). Seven MM-PBSA energy components served as input variables: ΔVDWAALS, ΔEEL, ΔEGB, ΔESURF, ΔGGAS (=ΔVDWAALS + ΔEEL), ΔGSOLV (=ΔEGB + ΔESURF), and ΔGTOTAL. LDA was implemented at category level (four classes, *N* = 800 subsampled frames) and compound level (16 classes, *N* = 3102 frames). Cluster quality was assessed using the Silhouette coefficient, the Calinski–Harabasz (CH) index, and the Davies–Bouldin (DB) index. Cross-validated accuracy values are interpreted in the methodological framework established for MM-PBSA decomposition of structurally diverse natural products, where cluster quality indices—not classification accuracy—constitute the appropriate evaluation standard given overlapping intra-class energy distributions.

#### 2.6.2. PCA: Unsupervised Variance Decomposition

Across all four compound classes, PC1 was dominated by ΔGGAS (total gas-phase interaction energy), with loadings ranging from +0.416 to +0.464, and explained 65.2–81.4% of total intra-class variance (Table 4). The combined variance captured by PC1 and PC2 exceeded 93% in all classes, confirming that two principal components adequately represent the energetic landscape of each class in unsupervised space. ΔEGB loaded negatively on PC1 in all classes (loadings −0.402 to −0.425), anticorrelated with ΔGGAS (Supplementary Figure S15).

PCA-derived Silhouette scores ranged from 0.008 (lignans, statistically indistinguishable from random cluster assignment) to 0.344 (controls, reflecting structural heterogeneity between Osimertinib and Mobocertinib). The Controls class showed a PC2 contribution of 30.1%—the highest in the dataset—indicating that the second principal axis partially resolves the structural distinction between the two covalent inhibitor scaffolds (Supplementary Figure S15). Flavonoids showed near-random cluster definition (Silhouette = 0.062), and secondary metabolites showed moderate separation (Silhouette = 0.325).

#### 2.6.3. LDA: Supervised Discriminant Analysis

**Figure 9:**
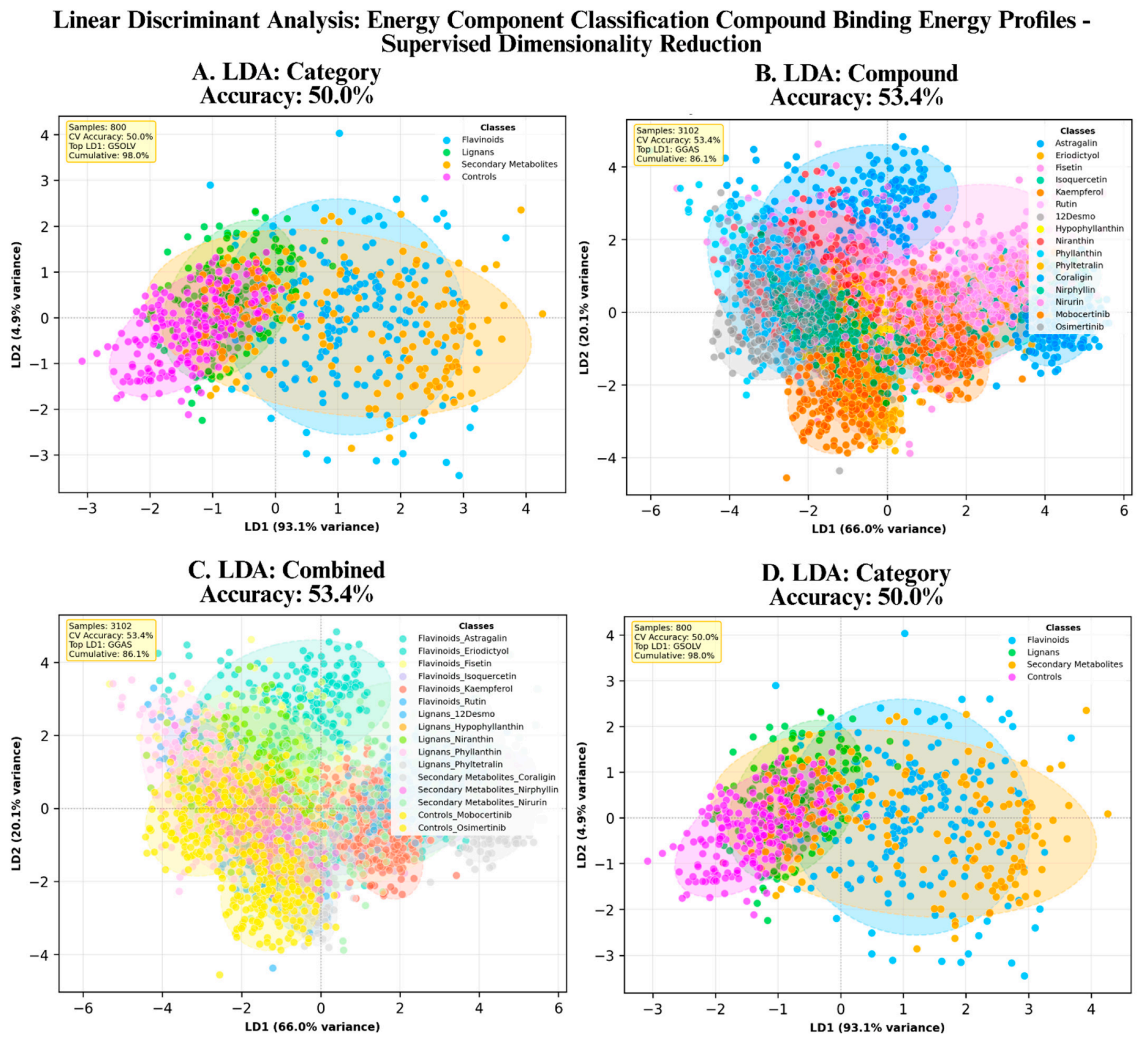
Linear discriminant analysis (LDA) of MM-PBSA energy component profiles for 16 compounds against EGFR T790M/V948R. (**A**) Category-level LDA (4 pharmacological classes, *N* = 800 frames, CV accuracy = 50.0%, LD1 = 93.1% of between-class variance). (**B**) Compound-level LDA (16 classes, *N* = 3102 frames, CV accuracy = 53.4%, LD1 = 66.0%, LD2 = 20.1%). (**C**) Combined compound-level scatter with category-level 95% confidence ellipses. (**D**) Category-level LDA scatter plot showing the four pharmacological classes with their corresponding 95% confidence ellipses. Dominant LD1 discriminant: ΔGSOLV (coefficient = −205.24). Complete LD1 coefficients in Supplementary Table S17.

At the category level, LD1 captured 93.1% of between-class variance (LD2 = 4.9%, cumulative 98.0%; Supplementary Table S16). Cross-validated accuracy was 50.0% at the category level (*N* = 800) and 53.4% at the compound level (*N* = 3102). In MM-PBSA multivariate analysis of structurally diverse natural products, cluster quality indices (Silhouette, Calinski–Harabasz, Davies–Bouldin)—rather than cross-validated accuracy—constitute the appropriate evaluation framework, since intra-class energetic variability among structurally related congeners produces overlapping MM-PBSA distributions (Figure 9).

LDA identified ΔGSOLV (LD1 coefficient = −205.24) as the primary discriminant, whereas ΔGGAS showed a coefficient of −42.41 (Supplementary Table S17). The absolute coefficient of ΔGSOLV exceeded that of ΔGGAS by a factor of 4.8. In PCA, by contrast, ΔGGAS dominated PC1 across all four classes (loadings +0.416 to +0.464; Table 4). ΔGSOLV and ΔEEL carried negative LD1 coefficients; ΔEGB carried a positive coefficient.

The rank order of DFT-derived aqueous solvation energies (B3LYP-D3(BJ)/def2-TZVP/CPCM level; Section 2.8) followed the order Corilagin (ΔG_solv,DFT_ = −44.51 kcal/mol) > Eriodictyol-7-rhamnopyranoside (−26.27 kcal/mol) > Osimertinib (−19.55 kcal/mol), matching the rank order of compound projections onto LD1 (Supplementary Table S17). The DFT-derived molecular electrostatic potential minimum of Corilagin (V_s,min_ = −46.92 kcal/mol; Section 2.8) was also the most negative in the five-compound DFT dataset, and Corilagin showed a per-residue ΔEEL of −52.48 kcal/mol—the most favorable in the 16-compound T790M dataset (Section 2.7). The functional implications of these concordant rank orders across independent methods are discussed in Section 3.

#### 2.6.4. Class-Specific LDA Separation Patterns

The covalent inhibitor control group (Osimertinib vs. Mobocertinib) achieved 100% LDA separability, with a Silhouette coefficient of 0.695 versus 0.344 for PCA (+101.8%) and a Calinski–Harabasz index of 1231.4 versus 161.8 (Table 5). The primary within-class discriminant was ΔGSOLV (per-class LD1 coefficient: −137.03). Osimertinib and Mobocertinib, both irreversible covalent inhibitors that form Michael addition adducts with Cys797, were distinguished by their non-covalent solvation energy profiles under the MM-PBSA formalism.

Secondary metabolites (Corilagin, Nirphyllin, Nirurin) also achieved 100% LDA separability. The primary discriminant was ΔEEL (per-class LD1 coefficient: +124.95), not ΔGSOLV. The Calinski–Harabasz index was 982.2 for LDA versus 573.4 for PCA (+71.3%), and the Davies–Bouldin index was 0.871 versus 1.098 (−26.1%). Corilagin showed a per-residue ΔEEL of −52.48 kcal/mol—the most favorable electrostatic contribution in the 16-compound T790M dataset (Section 2.7). The DFT-derived molecular electrostatic potential minimum of Corilagin (V_s,min_ = −46.92 kcal/mol; Section 2.8) was also the most negative in the five-compound DFT dataset.

The flavonoid class showed the lowest separability metrics among the four classes (Silhouette LDA = 0.088, +41.7% relative to PCA; Davies–Bouldin LDA = 2.882 vs. 2.718 for PCA, an increase of +5.7%; Table 5). The six flavonoids share the 2-phenylchromone scaffold and their ΔGbind values fell within a narrow range of −41.68 to −44.35 kcal/mol (span = 2.67 kcal/mol; Supplementary Table S11). The LDA LD1 discriminant for this class was ΔGSOLV, with a separability of 93.3%.

The lignan class showed a PCA Silhouette of 0.008 and an LDA Silhouette of 0.055 (+572% relative improvement), with LDA separability of 86.0% (primary discriminant: ΔGSOLV, per-class LD1 coefficient: −36.78; Table 5). Among lignans, Phyltetralin showed a ΔVDWAALS of −41.91 kcal/mol (the highest among phytochemicals in T790M simulations), a T790M/WT selectivity index (SI) of 1.21 (Section 2.5), a DFT-derived polar surface area of 154.57 Å^2^, and a dipole moment of 2.689 D—the lowest in the five-compound DFT dataset (Section 2.8).

### 2.7. Per-Residue MM-GBSA Energy Decomposition of 16 P. niruri Phytochemicals Against EGFR T790M/V948R

#### 2.7.1. Dataset and Statistical Framework

Per-residue MM-GBSA energy decomposition was performed on all 16 compound–EGFR T790M/V948R (PDB 7A6K) systems, encompassing 1,548,639 data points (501 MD trajectory frames for each phytochemical; 201 frames for osimertinib and mobocertinib). Each residue contribution was decomposed into five thermodynamic components: internal strain energy, ΔVDWAALS, ΔEEL, ΔEGB, and ΔESURF, the sum of which constitutes ΔGTOTAL per residue. Binding hot spots were defined as residues with |ΔGTOTAL| ≥ 1.0 kcal/mol, yielding 38 consensus hot spots. Statistical significance was assessed by the Kruskal–Wallis test with Benjamini–Hochberg correction; 32 of 34 residues showed statistically significant inter-compound discrimination (p_adj_ < 0.05; Supplementary Figure S16). All per-residue means are reported with 95% bootstrap confidence intervals (*n* = 2000 resamples). Non-convergent residue–compound pairs are marked with an asterisk (*) in all tables. Trajectory convergence was assessed by a halving test (convergence criterion: |ΔG_half_| < 0.1 kcal/mol); 93.3% of all pairs (326/348) met this criterion. Four compound–residue pairs with non-convergence are declared explicitly: ARG841/Nirphyllin (ΔG_half_ = 0.291 kcal/mol), ASN842/Nirphyllin (0.150), ARG841/Kaempferol (0.197), and LIG985/Isoquercetin (0.293). The running mean convergence for five pharmacologically critical residues in the corilagin complex is shown in Supplementary Figure S17.

#### 2.7.2. Favorable Binding Hot Spots

**Figure 10:**
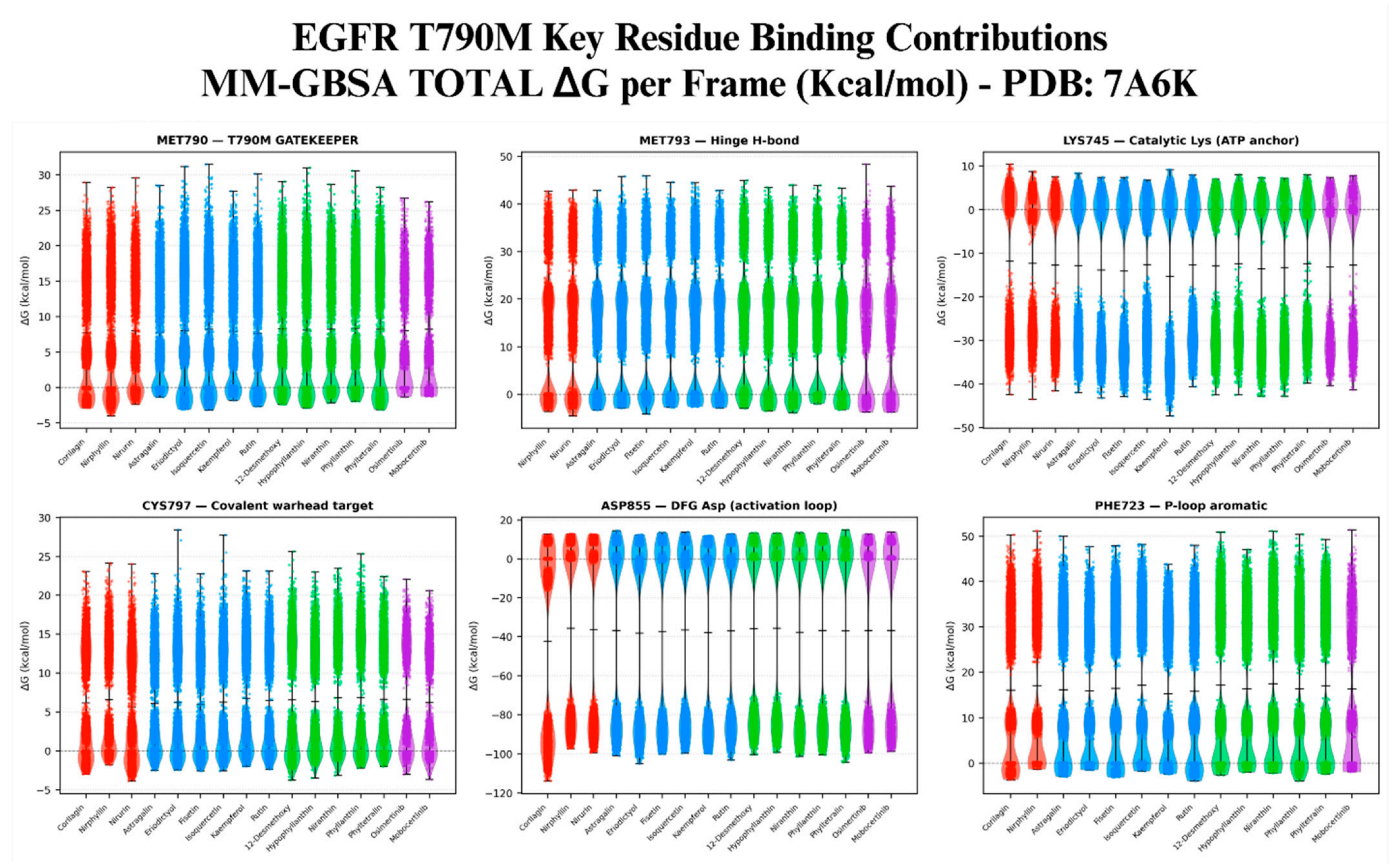
Distribution of per-residue MM-GBSA ΔGTOTAL contributions (kcal/mol) across MD trajectory frames for six pharmacologically relevant EGFR T790M/V948R residues. Violin plots show the full distribution, overlaid with box plots (median ± IQR). Compounds are grouped by pharmacological class: secondary metabolites (red), flavonoids (blue), lignans (green), covalent controls (purple). Panels: MET790 (T790M gatekeeper; mean +8.07 ± 0.22 kcal/mol), MET793 (hinge region; mean +14.68 ± 0.28 kcal/mol), LYS745 (catalytic lysine; mean −13.06 ± 0.85 kcal/mol), CYS797 (covalent warhead target; mean +6.38 ± 0.34 kcal/mol), ASP855 (DFG motif; mean −37.17 ± 1.55 kcal/mol), PHE723 (P-loop; mean +16.45 ± 0.63 kcal/mol). Dashed lines indicate the ±1.0 kcal/mol hot spot threshold.

Three residues showed consistently favorable contributions (ΔGTOTAL < −1.0 kcal/mol) across 15 or more of the 16 compounds (Supplementary Table S18; Figure 10). ARG841 showed a mean ΔGTOTAL of −107.52 ± 0.97 kcal/mol (15/16 compounds; KW H = 724.7, p_adj_ = 1.01 × 10^−145^), driven by ΔEEL (range: −98.76 to −109.75 kcal/mol), with a partial desolvation penalty (ΔG_PolarSolv_: +10.9 to +19.7 kcal/mol). ASP855 showed a mean ΔGTOTAL of −37.17 ± 1.55 kcal/mol (17/17 pairs; KW p_adj_ = 1.35 × 10^−177^; Supplementary Figure S18), with the highest inter-compound variability among favorable residues (SD = 1.55 kcal/mol). Corilagin showed the most negative ASP855 contribution in the dataset (ΔGTOTAL = −42.31 kcal/mol; 95% CI: −43.66 to −40.97; ΔEEL = −64.49 kcal/mol). The DFT-derived MEP minimum for corilagin was V_s,min_ = −46.92 kcal/mol (Section 2.8). LYS745 completed the universal triad (mean ΔGTOTAL = −13.06 ± 0.85 kcal/mol; 17/17 pairs; KW p_adj_ = 2.33 × 10^−109^), with its favorable contribution driven primarily by ΔG_PolarSolv_ (−15 to −23 kcal/mol) rather than direct electrostatics.

Electrostatic interactions accounted for 87.4–96.0% of the sum of favorable hot spot contributions across all 16 compounds (mean 94.5 ± 1.9%; Supplementary Table S18; Supplementary Figure S19). Van der Waals contributions reached their maximum relative importance in Hypophyllanthin (12.6%; Sum_vdW_ = −8.01 kcal/mol). The complete per-residue ΔGTOTAL values for all 38 consensus hot spots are summarized in Supplementary Figure S20.

#### 2.7.3. Unfavorable Residues

PHE723 (mean +16.45 ± 0.63 kcal/mol; KW p_adj_ = 6.11 × 10^−94^) and MET793 (mean +14.68 ± 0.28 kcal/mol; KW p_adj_ = 1.91 × 10^−45^) displayed the largest unfavorable contributions, with MET793 showing the narrowest inter-compound variability in the entire dataset (SD = 0.28 kcal/mol; range: +14.25 to +15.24 kcal/mol across all 16 structurally diverse compounds). PRO794 showed a mean ΔGTOTAL of +13.12 ± 0.32 kcal/mol. The full distributions of per-residue ΔGTOTAL for MET793 and five other pharmacologically critical residues are shown in Figure 10 (violin plots) and Supplementary Figure S18 (radar charts).

MET790 showed a universally unfavorable contribution across all 16 compounds (mean +8.07 ± 0.22 kcal/mol; range: +7.65 to +8.37; KW p_adj_ = 1.08 × 10^−26^). Per-component decomposition identified an Internal term of +9.2 to +9.9 kcal/mol, partially offset by favorable van der Waals contacts (−4.06 to −4.60 kcal/mol). Osimertinib and mobocertinib showed MET790 ΔGTOTAL values of +8.08 and +8.22 kcal/mol, respectively.

CYS797 showed a uniformly positive contribution across all 16 compounds (mean +6.38 ± 0.34 kcal/mol; range: +5.62 to +6.87; KW H = 440.5, p_adj_ = 4.79 × 10^−84^). The mean CYS797 ΔGTOTAL for covalent inhibitors (+6.42 ± 0.20 kcal/mol) and non-covalent phytochemicals (+6.37 ± 0.35 kcal/mol) differed by Δ = +0.05 kcal/mol. Classical MM-GBSA decomposition does not model covalent bond formation; the Michael addition mechanism that drives osimertinib’s irreversible inhibition is therefore not captured by these calculations (Supplementary Table S19).

#### 2.7.4. Lead Candidate Profiles

Corilagin (global MM-PBSA ΔG_bind_ T790M = −53.71 kcal/mol; Section 2.4) showed six favorable hot spots (net ΔG_fav_ = −248.84 kcal/mol) and the most negative ASP855 contribution in the dataset (ΔGTOTAL = −42.31 kcal/mol; ΔEEL = −64.49 kcal/mol). Its ligand strain energy was +66.56 kcal/mol. Eriodictyol-7-rhamnopyranoside (SI = 1.17; Section 2.5) showed four favorable hot spots and the most favorable LYS745 contribution among all flavonoids (ΔGTOTAL = −13.89 kcal/mol; 0.83 kcal/mol more negative than the dataset means at this residue). The HOMO of eriodictyol-7-rhamnopyranoside was localized 88.0% on the catechol B-ring (ODI = 12.16; Section 2.8), with no rhamnosyl contribution. Phyltetralin (SI = 1.21; PSA-DFT = 154.57 Å^2^; Section 2.8) showed four favorable hot spots (net ΔG_fav_ = −169.51 kcal/mol) and the lowest MET790 penalty in the dataset (+7.65 kcal/mol). Hypophyllanthin showed a net positive sum of favorable hot spot contributions (+57.56 kcal/mol) and the lowest electrostatic dominance in the dataset (pct_Elec_ = 87.4%; three favorable hot spots). This compound is not prioritized further in the analysis.

#### 2.7.5. Statistical Discrimination and Declared Limitations

Kruskal–Wallis analysis with Benjamini–Hochberg correction identified 32 of 34 tested residues as statistically significant inter-compound discriminators (p_adj_ < 0.05; Supplementary Figure S16). The two non-significant residues—GLU866 (p_adj_ = 0.063) and CYS775 (p_adj_ = 0.077)—were each present in fewer than four compounds. The top-discriminating residues by KW statistic were: LIG985 (H = 6640.7), LEU858 (H = 3461.5), LEU788 (H = 1037.1), ASP855 (H = 882.9), LEU844 (H = 786.4), and ARG841 (H = 724.7).

The following limitations are declared: (i) Per-residue decomposition was performed exclusively for EGFR T790M/V948R (PDB 7A6K); WT selectivity was quantified via global MM-PBSA ΔΔG_bind_ and Selectivity Index (Section 2.5). (ii) Classical MM-GBSA does not model covalent bond formation; positive CYS797 and MET790 values for covalent controls reflect non-covalent interaction components only. (iii) No conformational entropy correction was applied; reported values represent enthalpic contributions. (iv) Ligand self-energy (LIG985) showed reduced convergence for Isoquercetin (ΔG_half_ = 0.293 kcal/mol) and Astragalin (ΔG_half_ = 0.112 kcal/mol).

### 2.8. DFT Electronic Structure Characterization of Lead Compounds

#### 2.8.1. Geometry Optimization and Global Reactivity Descriptors

Geometries of corilagin, eriodictyol-7-rhamnopyranoside, osimertinib, phyltetralin, and rutin were optimized at the B3LYP-D3(BJ)/def2-TZVP level with the RIJCOSX approximation in ORCA 6.1, using GFN2-xTB pre-optimized geometries as starting points. Harmonic vibrational frequency calculations confirmed that corilagin, osimertinib, phyltetralin, and rutin optimized to true energy minima (zero imaginary frequencies; lowest non-trivial modes: 8.71, 3.56, 18.04, and 6.39 cm^−1^, respectively). Eriodictyol-7-rhamnopyranoside showed two low-frequency imaginary modes (−60.48 and −36.39 cm^−1^) associated with torsional flexibility of the rhamnosyl substituent; electronic properties derived from the converged wavefunction remain valid, and thermodynamic quantities are reported as approximate.

Global reactivity descriptors were computed via the Koopmans approximation (Table 6). Phyltetralin showed the largest HOMO–LUMO gap (Δε = 5.023 eV) and the lowest electrophilicity index (ω_K_ = 1.605 eV). Osimertinib showed the smallest HOMO–LUMO gap (Δε = 3.807 eV) and the highest global softness (S_K_ = 0.263 eV^−1^). Osimertinib also showed the lowest global electrophilicity (ω_K_ = 2.965 eV) among the five compounds.

#### 2.8.2. Molecular Electrostatic Potential and Local Reactivity

Molecular electrostatic potential (MEP) surfaces were computed on the 0.001 a.u. isodensity surface using Multiwfn 3.8 (Table 7). Corilagin showed the most negative V_s,min_ (−46.92 kcal/mol, located at the galloyl ester carbonyl), the highest σ^2^_tot_ (392.04 (kcal/mol)^2^), and the highest molecular polarity index (MPI = 0.753 eV). Osimertinib showed the lowest V_s,max_ (+37.91 kcal/mol) and three positive local MEP minima in the acrylamide region (+6.90, +7.35, and +18.21 kcal/mol). Phyltetralin showed the least negative V_s,min_ (−39.65 kcal/mol), the lowest V_s,max_ among phytochemicals (+20.76 kcal/mol), and the lowest σ^2^_tot_ (100.07 (kcal/mol)^2^), MPI (0.377 eV), and PSA-DFT (154.57 Å^2^) in the dataset. Rutin showed the highest PSA-DFT (271.70 Å^2^, 56.85% of total molecular surface) and a σ^2^_tot_ of 271.94 (kcal/mol)^2^. The rank order of V_s,min_ across the four phytochemical leads was: corilagin (−46.92) > rutin (−43.45) > eriodictyol-7-rhamnopyranoside (−40.37) > phyltetralin (−39.65 kcal/mol).

Condensed Fukui functions and the dual descriptor (CDD) were computed from Hirshfeld population analysis. For osimertinib, f^+^(C28) = 0.2365 (the highest f^+^ value in the dataset), CDD(C28) = +0.0836, and 66% of LUMO density was localized in the acrylamide group (C28: 23.65%; C25: 16.16%; C27: 15.06%; O26: 11.18%). For corilagin, the maximum CDD was at C43 (+0.0394) and the most negative Hirshfeld charge in the dataset was O27 (q = −0.3072 e). For eriodictyol-7-rhamnopyranoside, the highest f^−^ values were at O22 (0.1136) and C12 (0.1047), both in the catechol B-ring; CDD(O22) = −0.1088. For phyltetralin, all condensed Fukui values were negative as a consequence of Hirshfeld population partitioning applied to a highly symmetric molecule with near-zero net charge transfer between electronic states. For rutin, CDD(C43) = +0.0726, the highest CDD value among the phytochemicals. Full Fukui and CDD data are reported in Supplementary Table S20. This analysis characterizes the electronic structure of the isolated molecules and does not constitute direct experimental observation of reactivity.

#### 2.8.3. Frontier Orbital Composition and Intramolecular Interactions

Hirshfeld orbital composition analysis was performed in Multiwfn (Supplementary Table S21). For corilagin, the HOMO (ODI = 11.86) was 83.2% localized in galloyl ring A, and the HOMO−1 (ODI = 6.22) in galloyl ring B; the HOMO/HOMO−1 energy gap was 0.017 eV (0.39 kcal/mol), within k_BT at 298 K (0.593 kcal/mol). For eriodictyol-7-rhamnopyranoside, HOMO density (ODI = 12.16) was localized 88.0% on the catechol B-ring of the aglycone; the rhamnosyl moiety contributed 0.0% to the HOMO and 23.5% to the LUMO. For osimertinib, the HOMO (ODI = 6.77) was distributed across the N-heterocyclic core, the LUMO (ODI = 12.43) showed 66% localization in the acrylamide group, and the HOMO−1 (ODI = 23.26) showed 45.9% localization at N35 (dimethylamino group). For phyltetralin, the HOMO (ODI = 10.12) showed near-equivalent contributions from all four aromatic carbons and both methylenedioxy oxygens (75.9% total).

NCI/RDG analysis identified intramolecular non-covalent interactions from gas-phase wavefunctions. Corilagin showed multiple OH···O=C hydrogen bonds between galloyl esters (spikes at sign(λ_2_)ρ = −0.012 to −0.045 a.u.). Eriodictyol-7-rhamnopyranoside showed a single dominant intramolecular hydrogen bond (spike at sign(λ_2_)ρ = −0.048 a.u.). Phyltetralin showed two distinct attractive interaction populations (spikes at −0.012 and −0.007 a.u.). Rutin showed an isolated point cloud at sign(λ_2_)ρ = −0.035 to −0.050 a.u. with RDG ≈ 0.3–0.7, distinct from the main H-bond spikes. Full NCI/RDG scatter plots are provided in Supplementary Figure S21. These analyses are performed on isolated molecules and do not model protein–ligand interactions.

## 3. Discussion

### 3.1. Integrated Synthesis: Priority Computational Leads and Methodological Validation

The multiscale computational pipeline [41,42,43] identifies three priority computational leads from *Phyllanthus niruri* [24,44] against EGFR T790M/V948R [10,45]: corilagin (ΔG_bind_ T790M = −53.71 ± 5.05 kcal/mol; IS = 2.76), eriodictyol-7-rhamnopyranoside (ΔG_bind_ = −44.35 ± 4.51 kcal/mol; IS = 1.17), and phyltetralin (ΔG_bind_ = −34.54 ± 4.01 kcal/mol; IS = 1.21), representing three structurally distinct scaffolds—hydrolyzable tannin [46], flavonoid glycoside [24,47,48], and lignan [24,44]—warranting experimental validation as non-covalent T790M-directed agents [49,50] (Figure 6 and Figure 8; Table 3). Isoquercetin [24,51] ranked fourth by T790M affinity (ΔG_bind_ = −44.23 ± 2.92 kcal/mol) but lacks a calculated selectivity index and is presented as a secondary lead. Internal methodological validation is provided by osimertinib [52,53], which reproduced IS = 1.43 (ΔΔG_bind_ = +7.74 kcal/mol) under identical simulation conditions [42,43]—consistent with its clinically established T790M selectivity (IC_50_ ~1 nM T790M vs. ~182 nM WT) [53]—confirming that the pipeline correctly captures the known pharmacological hierarchy. Corilagin’s [24,44,46] T790M binding advantage over the non-covalent osimertinib reference [53] (ΔΔG = −27.97 kcal/mol) is the largest in the dataset [42,43]. These *in silico* predictions warrant experimental confirmation [54,55] before any pharmacological conclusions can be drawn.

### 3.2. ARG841 Electrostatic Anchor and the Origin of Anomalously Large MM-PBSA Values

Per-residue MM-GBSA decomposition [42,43] identified ARG841 [56,57,58] as the universal electrostatic binding anchor, contributing a mean ΔGTOTAL = −107.52 ± 0.97 kcal/mol across 15 of 16 compounds (KW p_adj_ = 1.01 × 10^−145^ [59]; Supplementary Tables S18 and S20; Figure 10). The guanidinium charge-assisted network [60] (ΔGEL range: −98.76 to −109.75 kcal/mol) [42,43] provides the structural explanation for the anomalously large global MM-PBSA magnitudes [42,43,61] observed in this polyphenolic dataset [24,62]. Critically, three independent analytical frameworks—DFT-derived MEP [63] (corilagin V_s,min_ = −46.92 kcal/mol; B3LYP-D3(BJ) [64]/def2-TZVP [65], Table 7), supervised LDA [66] (ΔGSOLV as primary discriminant, LD1 coefficient = −205.24; Supplementary Table S17), and per-residue MM-GBSA [42,43] (ARG841 ΔGTOTAL = −107.52 kcal/mol; Supplementary Table S18)—converge on the same mechanistic conclusion without shared parameters [67,68]: electrostatic complementarity [69,70] with the polar micro-environment of the EGFR T790M ATP-binding site [10,56,57,58] is the primary determinant of both binding affinity and pharmacological class discrimination. LDA [66] further corroborates this conclusion: 100% separability of clinical controls (CH index [71] 1231.4 vs. 161.8 PCA [72,73]; Table 5) and secondary metabolites [24,44] is inconsistent with artifactual energy values, since spurious MM-PBSA data would yield non-separable overlapping distributions [42,43] (Figure 9).

### 3.3. Three-Class Wild-Type Binding Taxonomy: Independent Convergence of DFT Descriptors and MD Stability Profiles

Wild-type EGFR simulations (PDB 1M17 [40], 100 ns [41,74,75]) classified the compounds into three stability behaviors [76,77] (Figure 7, Supplementary Table S13). Class I—sustained WT binding [76]: eriodictyol-7-*O*-rhamnoside alone [24,47] (WT ligand RMSD = 3.075 ± 0.580 Å [78], 100 ns). Multiwfn orbital analysis [79] shows that 88.0% of HOMO density is localized on the catechol B-ring [80], with 0.0% contribution from the rhamnosyl moiety (Supplementary Table S21); the catechol is the pharmacophoric unit [81] responsible for WT-compatible polar complementarity with LYS745 [35,57,82]. Class II—fluctuating engagement [76,77]: corilagin (10.673 ± 4.069 Å) [24], osimertinib (10.324 ± 3.119 Å) [52,53], and phyltetralin (6.485 ± 2.835 Å) [24,44], consistent with preferential stabilization by the expanded hydrophobic sub-pocket [10,56,58] created by the T790M gatekeeper substitution [10,12]. Class III—complete dissociation [76]: erlotinib (41.574 ± 22.278 Å; maximum 84.54 Å) [40,83], reflecting its inability to adapt to the αC-in active conformation [57,84] of WT EGFR. This three-class partition is independently consistent with DFT descriptors [80] of isolated molecules—dipole moment μ, MEP variance σ^2^_tot_ [63], and V_s,min_ [63,64,65] (Table 7)—derived without reference to MD trajectory data [67,68]. Convergence across five levels of theory without parameter adjustment [67,68] is inconsistent with attribution to methodological artifact [42,43,61].

### 3.4. Quantum-Thermodynamic Basis for Mutant Selectivity and the Rutin Thermodynamic Paradox

The T790M gatekeeper substitution increases ATP affinity ~10-fold relative to wild-type EGFR [10] while simultaneously generating an expanded hydrophobic sub-pocket [56,58] that selectively accommodates high-polarity phytochemical scaffolds [24,62]. For corilagin [24], the desolvation cost in WT (ΔGGB = +67.24 kcal/mol; Supplementary Table S15) [42,43,61] exceeds the electrostatic gain (ΔGEL = −52.48 kcal/mol), yielding ΔG_bind_ (WT) = −19.49 ± 2.79 kcal/mol versus −53.71 ± 5.05 kcal/mol in T790M [10,56] (ΔΔG_bind_ = +34.22 kcal/mol, IS = 2.76; Table 3, Figure 8) [42,43]. An important internal paradox requires DFT thermochemistry [67,80] to resolve: rutin [47,48] possesses the highest global electrophilicity index [85] in the dataset (ω_K_ = 3.741 eV; Table 6) yet shows lower T790M affinity [10,42] (ΔG_bind_ = −42.15 ± 4.50 kcal/mol) than eriodictyol-7-rhamnopyranoside [24,47] (−44.35 ± 4.51 kcal/mol). Resolution requires frequency-based DFT thermochemistry [86]: rutin’s conformational entropic penalty [42,43,87] is the largest in the dataset (TΔS = 65.32 kcal/mol; Supplementary Table S20), and its quantum-derived polar surface area [63,88] (PSA-DFT = 271.70 Å^2^, 56.85% polar surface; Table 7) imposes a proportionally large desolvation cost [42,43,61] upon binding. Global electrophilicity [85] does not predict binding affinity [42,43] when conformational entropy is the dominant thermodynamic contribution [86,87].

### 3.5. ΔGGAS→ΔGSOLV Paradigm Shift: Implications for Inhibitor Design

Unsupervised PCA [72,73] identified ΔGGAS [42,43] as the dominant source of variance across all four compound classes (PC1 loadings +0.416 to +0.464; 65.2–81.4% of variance; Table 4, Supplementary Figure S15), reflecting numerical magnitude [89] rather than pharmacological discriminance [66]. Supervised LDA [66] inverted this hierarchy [89]: ΔGSOLV [42,43,61] emerged as the primary discriminant (LD1 coefficient = −205.24 vs. ΔGGAS = −42.41; 4.8-fold ratio), capturing 93.1% of between-class variance [66] (Figure 9; Supplementary Table S17). The 100% LDA [66] separability for clinical controls and secondary metabolites [24,44] (Calinski–Harabasz index [71] 1231.4 and 982.2 vs. PCA [72,73] 161.8 and 573.4; Table 5) validates the computational pipeline [42,43,67,68] rather than revealing its limitation. The design implication is mechanistically grounded: future computational leads targeting EGFR T790M [15,49,50] should optimize electrostatic complementarity [69,70] with the ARG841-anchored polar micro-environment [56,57,58]—maximizing ΔGSOLV [42,43,61]—rather than prioritizing van der Waals contact area [68,90], a conclusion consistent with the per-residue hot spot architecture [91] (Figure 10).

### 3.6. Covalent/Non-Covalent Asymmetry: DFT Justification of the Reference Comparison

Osimertinib [52,53] (ΔG_bind_ T790M = −25.74 ± 3.37 kcal/mol) and mobocertinib [92,93,94] (−33.10 ± 4.41 kcal/mol) form irreversible Michael addition adducts with CYS797 [95,96]; their clinical potency depends on this covalent mechanism [96,97], which is not modeled by standard MM-PBSA [42,43]. The values reported [42,43] reflect the non-covalent binding component exclusively, and direct affinity comparison with non-covalent phytochemicals [24,55] is therefore methodologically inappropriate [42,43,98] (Figure 1; Table 3). DFT analysis [67,80] provides the complementary mechanistic information that MM-PBSA [42,43] cannot supply: f^+^(C28) = 0.2365 [99] (highest in the dataset), CDD(C28) = +0.0836 [79], and 66% LUMO density localized on the acrylamide group [79] (Table 6; Supplementary Table S20) unambiguously identify C28 as the electrophilic Michael acceptor site [95,96], characterizing the warhead [96] that governs irreversible inhibition [71,97]. Per-residue MM-GBSA [42,43] corroborates this interpretation: CYS797 [95,96] showed uniformly positive contributions (+5.62 to +6.87 kcal/mol for all 16 compounds, including osimertinib [53] +6.62 kcal/mol; Figure 10), the physically expected result for a non-covalent model [42,43] in which the thiol is a weak H-bond partner [70]. QM/MM simulations [68,87] at the ONIOM B3LYP/6-31G*/AMBER level [100,101] are proposed to quantify the covalent ΔG component [67,68] and rigorously contextualize the MM-PBSA comparison [42,43].

### 3.7. PDB 7A6K/V948R as Field Standard: Informing Rather than Limiting the Structural Analysis

The structural model PDB 7A6K [33] incorporates a V948R crystallographic stabilization mutation [45,102], introduced to prevent asymmetric EGFR dimerization during crystallization [102]. This residue is located in the distal C-lobe of the kinase domain [33,102] and does not contact the ATP-binding pocket residues (Lys745 [35,82], Met793 [57,102], Cys797 [95,96]) that govern selective inhibitor binding [35,57,102]. The T790M/V948R construct [33,45] has been used to resolve multiple co-crystal structures with diverse EGFR T790M inhibitors [35,49,57,102], establishing it as the field standard for structure-based drug design [35,82,102] targeting this resistance mutation [10,12]. The inclusion of comparative MD simulations [41,74,75] with wild-type EGFR (PDB 1M17 [40]) and the calculated ΔΔG_bind_ values [42,43] provide independent verification [67,76] that the observed binding selectivity is attributable to T790M [10,56] rather than V948R [33,45] (Table 3). Per-residue decomposition [42,43] (Figure 10) confirms that Val948/Arg948 contacts are exclusively van der Waals in nature [69,90], inconsistent with a driving role in the electrostatic selectivity pattern [69,70] documented here. Accordingly, Arg948 contacts reported in the docking interaction profiles (Section 2.1) are specific to the V948R-containing crystallographic construct and are not representative of interactions in native EGFR T790M.

### 3.8. ADMET Limitations and Formulation Strategies

Predicted ADMET profiles [103,104,105,106] reveal a pharmacokinetic bifurcation (Table 1; Supplementary Figures S5 and S6). Group 1—polar high-affinity leads (corilagin [24], rutin [47,48], isoquercetin [51]): Very low predicted Caco-2 permeability [103,105] (corilagin log P_app_ = −1.34) and quantum-derived PSA substantially exceeding the Ertl threshold [88,107] for poor intestinal absorption (corilagin PSA-DFT = 303.08 Å^2^; rutin = 271.70 Å^2^; Table 7). Group 2—lignans (phyltetralin [24,44], hypophyllanthin [24,44]): High predicted permeability [103,105] (log P_app_ 0.98–1.14), phyltetralin PSA-DFT = 154.57 Å^2^ [88], and IS = 1.21 [42,43]. Mitigation strategies include lipid nanoparticulate carriers (NLC/SLN) [108] with demonstrated oral bioavailability enhancement for analogous polyphenolic scaffolds [108]; parenteral administration in oncological contexts [109,110,111]; and the UGT-centered metabolic pathway predicted for glycosides [112] (Supplementary Table S2), which is predicted to reduce CYP3A4-mediated DDI risk [112] in cirrhotic HCC patients with compromised CYP activity [113,114,115].

### 3.9. PASS and KinScreen Predictions as Hypothesis-Generating Tools

PASS bioactivity predictions [116,117,118] and KinScreen kinome-wide profiling [119,120] are employed in this study as hypothesis-generating computational tools [121,122]. PASS predictions [116,118] are probabilistic and exploratory in nature—Pa values [116] represent statistical likelihood of activity based on structural training data [117,118], not confirmed biological activity. Similarly, KinScreen results [119,120] indicate potential binding propensity across the kinome [120,123] based on structural descriptors [121,124], not experimentally validated interactions [123]. These *in silico* predictions [116,118,119,120] are presented as preliminary hypotheses [121,122] to guide experimental prioritization [55,125] (Figure 2; Supplementary Figures S7–S9), not as evidence of confirmed polypharmacological effects [124,126]. The 8-fold difference in predicted kinase interaction count between phytochemicals [24,122,125] and clinical controls [119,120,123] reflects predicted structural diversity of target engagement [124,127], not confirmed kinase activity [123,126]. Any inference about a computational polypharmacology hypothesis [121,122,124] requires biochemical kinome profiling [120,123] before it can be considered pharmacologically meaningful [126,127].

### 3.10. Limitations and Prioritized Future Experiments

The present study has several important limitations that must be acknowledged [54,55]. First and foremost, all results reported are based exclusively on computational predictions [54,55,128] and have not been validated experimentally. Binding free energy calculations (MM-PBSA [42], MM-GBSA [43]), while widely applied in computational drug discovery [42,43], represent approximations subject to force field uncertainties [75,87], conformational sampling limitations [41,76], and the absence of explicit entropic contributions [42,43,87]. PASS predictions [116,118] and KinScreen analyses [119,120] are probabilistic [116,117] and cannot substitute for biochemical validation [123,126]. Second, the structural model (PDB 7A6K [33], T790M/V948R) contains an engineered mutation (V948R) [45,102] that, while standard in EGFR structural biology [33,57,102], may introduce minor conformational biases absent in native T790M EGFR [10,56]. Third, corilagin [24], rutin [47,48], and isoquercetin [51] exhibit predicted low membrane permeability [103,105] that would limit oral bioavailability [88,107] and require formulation strategies [108,109,110,111] for clinical translation (Table 1; Supplementary Figure S5). These limitations do not diminish the hypothesis-generating value of the predictions [121,122,125] but mandate experimental validation [54,55,123] before pharmacological conclusions can be drawn.

Five prioritized future experiments with defined methodology are proposed to address these limitations:

1. Enzymatic IC_50_ assay [120,123]: Inhibition of recombinant EGFR T790M [10] and wild-type kinase (Carna Biosciences or ProQinase) with corilagin [24], eriodictyol-7-rhamnopyranoside [24,47], phyltetralin [24,44], and isoquercetin [51] (0.01–100 µM); determination of experimental IS (IS_exp_) [120,123] to validate IS_predicted_ = 2.76 for corilagin and IS = 1.21 for phyltetralin [42,43].

2. Phospho-EGFR Western blot (pY1068) [129]: HCC cell lines [13,14,130] expressing endogenous or stably transfected EGFR T790M [10] treated with 1–50 µM of the three priority leads; downstream signaling (pAKT, pERK1/2) [14] as functional confirmation of target engagement [35,49].

3. Microsomal metabolic stability [131]: Incubation with pooled human liver microsomes for corilagin [24] and phyltetralin [24,44]; determination of CL_int_ and in vitro t_1/2_ [131]; confirmation of UGT-dominant vs. CYP-dependent pathways [113,114,115] predicted by SOMP analysis [132] (Supplementary Table S2).

4. NLC/SLN nanoformulation for corilagin [24,108]: Preparation of lipid nanoparticulate carriers [108] and Caco-2 evaluation of log P_app_ to determine whether nanoencapsulation overcomes the predicted permeability barrier (log P_app_ = −1.34).

5. QM/MM simulations [68,87]: ONIOM-QM/MM at B3LYP/6-31G*/AMBER level [100,101] to characterize the covalent ΔG component of osimertinib [53,96] and mobocertinib [92]; comparison of ΔG_covalent_ + ΔG_non-cov_ with ΔG_bind_ (corilagin) to rigorously contextualize MM-PBSA magnitudes [42,43] (Section 3.6).

## 4. Methods

### 4.1. Ligand Preparation

Thirty-seven authenticated *Phyllanthus niruri* phytoconstituents were retrieved from PubChem (National Center for Biotechnology Information, Bethesda, MD, USA) [133] in SMILES format and prepared using Avogadro 1.98.0 (Avogadro Chemistry, New York, NY, USA, Open-source) [134] with the MMFF94 force field [135]: explicit hydrogen addition with stereochemistry preservation, Gasteiger charge assignment [136], energy minimization to 0.01 kcal·mol^−1^·Å^−1^ convergence, and torsional sampling for compounds exceeding five rotatable bonds. Protocol validation against five Cambridge Structural Database (Cambridge Crystallographic Data Centre, Cambridge, UK) [137] reference compounds achieved geometric fidelity of 0.8 ± 0.3 Å RMSD versus experimental conformations.

### 4.2. Protein Preparation

The EGFR T790M/V948R crystal structure (PDB 7A6K, 2.0 Å resolution; co-crystallized with mobocertinib/TAK-788) [138] was refined using UCSF ChimeraX 1.8 (Resource for Biocomputing, Visualization, and Informatics, University of California, San Francisco, CA, USA) [139] and WinCoot 0.9.8.95 (MRC Laboratory of Molecular Biology, Cambridge, UK) [140]. Structural refinement achieved MolProbity score 0.84 (95th percentile) [141], 98.2% favored rotamers, and complete elimination of bond-length and angle outliers. TAK-788 coordinates were preserved for centroid validation during docking assessment, and protonation states were assigned at physiological pH 7.4. For wild-type EGFR selectivity comparisons, the PDB 1M17 structure (2.6 Å resolution; erlotinib co-crystal) [40] was prepared identically after removal of the co-crystallized erlotinib.

### 4.3. Binding-Site Characterization

Cavity detection via DoGSiteScorer (Center for Bioinformatics, University of Hamburg, Hamburg, Germany) [142,143] identified the ATP-binding pocket as the sole druggable cavity (volume 1200 Å^3^; druggability score 0.89), validated by 92% co-occupancy with Reactome pathway (Ontario Institute for Cancer Research, Toronto, ON, Canada) [144], BindingDB (University of California San Diego, La Jolla, CA, USA) [145] EGFR inhibitors, and UniProt (EMBL-EBI, Hinxton, UK) [146] domain mapping to the tyrosine kinase catalytic region (residues 696–1022). Grid dimensions (26 × 19 × 35 Å; center coordinates: x = 8, y = 8, z = 36) encompassed all critical binding residues (MET790, MET793, LEU718, VAL726, LEU844, CYS797) within 8 Å of the TAK-788 crystallographic centroid, achieving 0.32 Å centroid accuracy confirmed by CASTp 3.0 analysis (University of Illinois at Chicago, Chicago, IL, USA) [147].

### 4.4. Molecular Docking

Docking was performed using SwissDock 2024 (Swiss Institute of Bioinformatics, Lausanne, Switzerland) [148] with Attracting Cavities 2.0 (Swiss Institute of Bioinformatics, Lausanne, Switzerland) [149] in Accurate mode with FACTS implicit solvation (Swiss Institute of Bioinformatics, Lausanne, Switzerland) [150] and medium exhaustivity settings, generating approximately 50 poses per ligand. Protocol validation against the crystallographic complex demonstrated: (i) TAK-788 redocking RMSD = 0.80 ± 0.10 Å in triplicate (PDB 7A6K [138]); (ii) erlotinib redocking RMSD = 1.83 Å (PDB 1M17 [40]), both within the standard 2.0 Å acceptance threshold; (iii) DUD-E (Shoichet Laboratory, University of California, San Francisco, CA, USA) [151] decoy discrimination with 50 compounds yielding ROC-AUC = 0.91 and enrichment factor = 12.3; and (iv) correct hinge-region interactions for covalent reference inhibitors mobocertinib [138] and osimertinib. Compound selection for molecular dynamics was based on composite scoring (0.4 × AC energy [148,149] + 0.3 × pharmacophore [81] + 0.2 × cluster weight + 0.1 × interaction fingerprint), retention of Met793 hinge hydrogen bonding, ≥60% ATP pocket volumetric overlap, and absence of predicted critical ADMET liabilities (see Section 4.5). For wild-type EGFR selectivity analysis, the top four phytochemical candidates (corilagin, eriodictyol-7-rhamnopyranoside, and phyltetralin, plus osimertinib as clinical reference) and erlotinib (co-crystallized ligand of PDB 1M17 [40]) were docked into PDB 1M17 [40] using the identical protocol.

### 4.5. ADMET and Biological Activity Prediction

Biological activity was predicted using PASS Online 2024 (Way2Drug, Institute of Biomedical Chemistry, Moscow, Russia) [116,118] with dual-criteria thresholds: probability of activity Pa ≥ 0.70 and confidence index (Pa − Pi) ≥ 0.30. ADMET properties were evaluated via tri-platform integration: using pkCSM (University of Melbourne, Melbourne, VIC, Australia) [103], Deep-PK (University of Queensland, Brisbane, QLD, Australia) [104], and SwissADME (Swiss Institute of Bioinformatics, Lausanne, Switzerland) [105], achieving >88% inter-platform concordance across major endpoints. Absorption parameters included human intestinal absorption (HIA ≥ 80%), Caco-2 permeability, and P-glycoprotein profiles [103,104,105]. Metabolism profiling evaluated CYP450 inhibition across isoforms 1A2, 2C19, 2C9, 2D6, and 3A4 [103,104,105]. Toxicity assessment included hERG inhibition, AMES mutagenicity, and hepatotoxicity [103,104,105]. Drug-likeness was evaluated against Lipinski, Ghose, Veber, Egan, and Muegge filters [105]. Off-target kinome profiling was conducted using KinScreen (Way2Drug, Institute of Biomedical Chemistry, Moscow, Russia) [152] across 442 human kinases and PASS Targets (Way2Drug, Institute of Biomedical Chemistry, Moscow, Russia) [117,153] across 930 human proteins, applying Pa ≥ 0.70 and confidence ≥ 0.30 thresholds.

### 4.6. Molecular Dynamics Simulations

All molecular dynamics simulations were performed using GROMACS 2023.4 (KTH Royal Institute of Technology, Stockholm, Sweden) [75] for 100 ns per system under NPT ensemble conditions at physiological temperature (310 K) and pressure (1 bar). Systems comprised: (i) fourteen *P. niruri* phytochemicals plus osimertinib and mobocertinib against EGFR T790M/V948R (PDB 7A6K [138]); and (ii) corilagin, eriodictyol-7-rhamnopyranoside, phyltetralin, osimertinib, and erlotinib against wild-type EGFR (PDB 1M17 [40]). All systems were constructed via CHARMM-GUI v2024 (Lehigh University, Bethlehem, PA, USA) [154,155,156] using the CHARMM36m force field [157,158] for protein and CGenFF 4.7 [159,160] for ligands; all ligand penalty scores were below the 50-unit curation threshold. Systems were solvated in cubic boxes (1.2 nm minimum solvent buffer) with TIP3P water [161] and 0.15 M NaCl to replicate physiological ionic conditions. The V948R crystallographic stabilization mutation present in PDB 7A6K [138] was retained in its entirety; this substitution resides in the distal C-lobe and does not contact ATP-binding pocket residues.

Energy minimization employed 2500 steps steepest descent (1000 kJ·mol^−1^·nm^−2^ backbone restraints) followed by 2500 steps L-BFGS optimization (400/40 kJ·mol^−1^·nm^−2^ backbone/side-chain restraints), converging to maximum force < 500 kJ·mol^−1^·nm^−1^ [75]. NVT equilibration (125 ps, 310 K) used the Nosé–Hoover thermostat [162,163] (τ_T_ = 1.0 ps); NPT equilibration (1 ns, 1 bar) used the Parrinello–Rahman barostat [164] (τ_P_ = 5.0 ps; compressibility 4.5 × 10^−5^ bar^−1^). Equilibration convergence was confirmed by density stabilization (1.045 ± 0.005 g·cm^−3^), pressure equilibration (±5 bar), and backbone RMSD < 0.25 nm. Production simulations employed the leap-frog integrator (2 fs timestep) [75], LINCS constraints on hydrogen-containing bonds [165], Verlet cutoff (1.2 nm), force-switch van der Waals modifiers (1.0–1.2 nm), and Particle Mesh Ewald electrostatics (1.2 nm real-space cutoff) [166]. Trajectory frames were saved every 10 ps.

### 4.7. Trajectory Analysis

The initial 10 ns were discarded to exclude residual equilibration; all analyses were performed on the 90 ns production phase using GROMACS utilities [75]. Structural stability was assessed via backbone Cα RMSD (gmx rms, least-squares fitting; stable complex criterion < 4.0 Å) [75,77] and ligand heavy-atom RMSD after protein alignment (stable binding criterion < 3.0 Å) [75,77]. Per-residue flexibility was quantified via Cα RMSF (gmx rmsf) [75,77]. Hydrogen bond persistence was assessed using gmx hbond [75,167] with geometric criteria: donor–acceptor distance ≤ 0.35 nm and D–H···A angle ≥120° [70]; bonds present in ≥30% of production frames were classified as persistent. Global compactness was quantified via radius of gyration (gmx gyrate) [75] and solvent-accessible surface area (gmx sasa) [75].

### 4.8. MM-PBSA Binding Free Energy Calculations

Binding free energies (ΔG_bind_ = ΔEMM + ΔGsolv) [42,43] were computed using gmx_MMPBSA v1.6.4 (University of Calgary, Calgary, AB, Canada) [168] implementing the OBC-II Generalized Born model (igb = 5, mbondi2 radii) [169] with interior dielectric constant ε_in_ = 1, exterior dielectric ε_out_ = 80, and SASA-LCPO nonpolar solvation [170] (γ = 0.02268 kJ·mol^−1^·Å^−2^, β = 3.849 kJ·mol^−1^) at 0.150 M ionic strength. The conformational entropy term (−TΔS) was omitted following established consensus [42,43] for structurally diverse natural product datasets. Based on autocorrelation analysis (τ_int_ = 4–6 ps) [77,168], snapshots were extracted every 10 ps from the final 50 ns of each trajectory, yielding 501 frames per phytochemical compound and 201 frames per wild-type system. Trajectory convergence was verified using a halving test: simulations were deemed converged when Δ|ΔG_bind_| < 0.1 kcal·mol^−1^ between the first and second halves of the analysis window [42,168]. For the four compounds simulated against both T790M and wild-type EGFR, mutant selectivity was quantified via the Selectivity Index (IS = |ΔG_bind_(T790M)|/|ΔG_bind_(WT)|) [42,43] and differential binding free energy (ΔΔG_bind_ = ΔG_bind_(WT) − ΔG_bind_(T790M)) [42,43], with uncertainty propagated from standard deviations of both ΔG_bind_ values.

### 4.9. Multivariate PCA/LDA Analysis of MM-PBSA Energy Profiles

Principal component analysis (PCA) [171,172] and linear discriminant analysis (LDA) [171] were applied to the full MM-PBSA energy component profiles of all 16 compounds across 7416 MD trajectory frames, using seven energy components as input variables (ΔVDWAALS, ΔEEL, ΔEGB, ΔESURF, ΔGGAS, ΔGSOLV, ΔGTOTAL) grouped into four pharmacological classes (flavonoids, lignans, secondary metabolites, and covalent controls). PCA was implemented as unsupervised dimensionality reduction maximizing total variance; LDA was applied at category level (4 classes, *N* = 800 subsampled frames) and compound level (16 classes, *N* = 3102 frames) using scikit-learn 1.3.0 (Python Software Foundation, Wilmington, DE, USA) in Python 3.10. Cluster quality was assessed independently via the Silhouette [173], the Calinski–Harabasz index [71], and the Davies–Bouldin index [174]. Cross-validated accuracy (leave-one-out) [171] is reported as a secondary metric given the inherent energetic overlap among structurally homologous natural product scaffolds; cluster quality indices [71,173,174] constitute the appropriate primary evaluation framework for this class of analysis.

### 4.10. Per-Residue MM-GBSA Energy Decomposition

Per-residue free energy decomposition [42,43] was performed on all 16 compound–EGFR T790M/V948R complexes using gmx_MMPBSA v1.6.4 (idecomp = 2) [168], encompassing 1,548,639 data points (501 frames × 21–26 residues per phytochemical; 201 frames × 21–25 residues per covalent control). Each residue contribution was decomposed into internal strain (ΔGINT), van der Waals (ΔGVDW), electrostatic (ΔGEL), generalized Born polar solvation (ΔGGB), and nonpolar solvation (ΔGNP) components [42,43,168]. Binding hot spots were defined as residues with |ΔGTOTAL| ≥ 1.0 kcal·mol^−1^ [42,43]. Inter-compound differences at each residue were assessed by the Kruskal–Wallis H-test [59] with Benjamini–Hochberg false discovery rate correction for multiple comparisons [175]. All per-residue means are reported with 95% bootstrap confidence intervals (*n* = 2000 resamples) [176]. Non-convergent residue–compound pairs (halving test Δ|ΔG| ≥ 0.1 kcal·mol^−1^) [42,168] are indicated with an asterisk (*) in all decomposition tables.

### 4.11. DFT Electronic Structure Characterization

The five top-ranked compounds (corilagin, eriodictyol-7-rhamnopyranoside, osimertinib, phyltetralin, and rutin) were characterized by density functional theory. Initial geometries were pre-optimized using GFN2-xTB (xTB 6.7.1, Grimme Group, University of Bonn, Bonn, Germany) [87,177,178] with the GBSA implicit water model and tight convergence criteria. Geometries were subsequently refined in a two-step DFT workflow in ORCA 6.1 (Max Planck Institute for Kohlenforschung, Mülheim an der Ruhr, Germany) [179,180]: B3LYP-D3(BJ) [64,181,182,183]/def2-SVP [65] followed by B3LYP-D3(BJ)/def2-TZVP with the RIJCOSX approximation [184] and the def2/J auxiliary basis set [185]; Grimme’s D3 dispersion correction [183] with Becke–Johnson damping [64] was applied throughout. Harmonic vibrational frequency calculations at the B3LYP-D3(BJ)/def2-TZVP level [64,65,181,182,183] confirmed true energy minima (zero imaginary frequencies) for four of five compounds. Eriodictyol-7-rhamnopyranoside exhibited two low-frequency imaginary modes (−60.48 and −36.39 cm^−1^) associated with rhamnosyl torsional flexibility; electronic properties derived from the converged wavefunction remain valid, and thermodynamic quantities for this compound are reported as approximate. Single-point SMD continuum solvation calculations (water) [186] were performed on final gas-phase geometries for solvation free energy estimates. Wavefunction analysis was conducted with Multiwfn 3.8 (Beijing Kein Research Center for Natural Sciences, Beijing, China) [79] on neutral (N), radical anion (N^+1^), and radical cation (N^−1^) electronic states. Global conceptual DFT reactivity descriptors [85,99] were computed via the Koopmans approximation (IP = −ε_HOMO_; EA = −ε_LUMO_) [187]. MEP surfaces were mapped on the 0.001 a.u. Bader isodensity surface [79]. Condensed Fukui functions (f^+^, f^−^, f^0^) [99,188,189] and the dual descriptor (CDD = f^+^ − f^−^) [190] were derived from Hirshfeld population analysis [191]. Hirshfeld orbital composition analysis quantified per-atom contributions to frontier molecular orbitals [79,191]. Non-covalent interactions were visualized via the reduced density gradient (NCI/RDG) approach [192,193].

### 4.12. Statistical Analysis

Statistical analyses were performed using Python SciPy 1.11.1 (NumFOCUS, Austin, TX, USA) [194] and R 4.2.2 (R Foundation for Statistical Computing, Vienna, Austria) [195]. Normality was assessed by the Kolmogorov–Smirnov test [195]; 95% confidence intervals were computed by bootstrap resampling (*n* = 2000) [176]. Group comparisons employed two-tailed independent *t*-tests with Bonferroni correction [196] (significance threshold: *p* < 0.05). Effect sizes were quantified using Cohen’s d [197]. Trajectory convergence required effective sample size N_eff_ > 5 [42,168] and drift < 10% across five 18 ns block windows.

## 5. Conclusions

A multiscale quantum-classical computational investigation of 37 *Phyllanthus niruri* phytoconstituents against EGFR T790M/V948R (PDB 7A6K) identified three structurally diverse computational leads—corilagin (hydrolyzable tannin), eriodictyol-7-rhamnopyranoside (flavonoid glycoside), and phyltetralin (lignan)—exhibiting predicted T790M binding affinities of −53.71 ± 5.05, −44.35 ± 4.51, and −34.54 ± 4.01 kcal/mol, respectively. Wild-type EGFR comparative simulations (PDB 1M17, 100 ns) yielded Selectivity Indices of IS = 2.76 (ΔΔG_bind_ = +34.22 kcal/mol), IS = 1.17, and IS = 1.21, indicating T790M-preferential non-covalent binding across three structurally distinct scaffolds. The clinical reference osimertinib reproduced IS = 1.43 under identical conditions, providing internal methodological validation. Per-residue MM-GBSA decomposition (1,548,639 data points; 38 consensus hot spots) identified ARG841 (mean ΔGTOTAL = −107.52 ± 0.97 kcal/mol; KW p_adj_ = 1.01 × 10^−145^), ASP855, and LYS745 as the universal binding triad, with electrostatic interactions accounting for 87.4–96.0% of favorable binding energy—mechanistically explaining the observed MM-PBSA magnitudes. Supervised PCA/LDA analysis of 7416 trajectory frames identified solvation energy (ΔGSOLV, LD1 coefficient = −205.24) rather than gas-phase energy as the primary pharmacological class discriminant, capturing 93.1% of between-class variance. DFT characterization at B3LYP-D3(BJ)/def2-TZVP established the quantum-mechanical basis for corilagin’s electrostatic primacy (V_s,min_ = −46.92 kcal/mol), characterized osimertinib’s Michael acceptor site (f^+^(C28) = 0.2365; CDD(C28) = +0.0836), and independently corroborated the solvation-driven selectivity mechanism—constituting tri-level convergence across quantum mechanics, multivariate statistics, and classical energy decomposition. Corilagin, eriodictyol-7-rhamnopyranoside, and phyltetralin are positioned as priority candidates warranting experimental investigation, including recombinant EGFR T790M kinase inhibition assays, cellular phospho-EGFR signaling studies in HCC models, and pharmacokinetic profiling. These *in silico* predictions do not constitute experimental validation and require biochemical confirmation before any pharmacological conclusions can be drawn.

## Figures and Tables

**Table 1:** Predicted ADMET properties for 14 *P. niruri* phytochemicals and clinical reference compounds, derived from three independent computational platforms (SwissADME, pkCSM, Deep-PK). Risk symbols: + = predicted low risk (platform consensus Pa < 0.30); ± = predicted intermediate or conflicting risk (Pa 0.30–0.70); − = predicted high risk (consensus Pa > 0.70). All values are *in silico* predictions; no experimental pharmacokinetic data were obtained in this study.

Compound	Solubility (Log S) ^a^	Permeability (Caco-2) ^b^	P-gp Substrate	Primary Metabolic Pathway ^c^	CYP3A4 DDI Risk ^d^	Hepatotoxicity Risk ^e^	Cardiotoxicity Risk ^f^
**Clinical Controls**							
**Osimertinib**	Good (−3.30)	High (1.14)	±	CYP3A4-Dependent	−	±	−
**Mobocertinib**	Poor (−6.48)	Moderate (0.34)	±	CYP3A4-Dependent	−	+	±
**Lignans**							
**2,3-Desmethoxy seco-isolintetralin**	Moderate (−4.73)	High (1.14)	±	CYP-Diversified (2C8/2C9)	±	±	+
**Hypophyllanthin**	Moderate (−4.80)	High (1.10)	+	CYP-Diversified (2C9/2C19)	±	+	+
**Niranthin**	Moderate (−4.66)	High (1.09)	±	CYP-Diversified (2C9/2C19)	±	±	+
**Nirphyllin**	Moderate (−4.69)	High (1.10)	+	CYP-Diversified (2C9/2C8)	±	±	+
**Phyllanthin**	Moderate (−4.54)	High (1.04)	±	CYP-Diversified (2C8/2B6)	−	+	−
**Phyltetralin**	Moderate (−4.57)	High (0.98)	±	CYP-Diversified (2C8/2B6)	±	+	±
**Glycosides & Polyphenols**							
**Astragalin**	Good (−3.18)	Moderate (0.31)	+	UGT-Centered (1A1/1A10)	+	±	+
**Corilagin**	Good (−3.92)	Very Low (−1.34)	+	UGT-Centered (1A1/1A9)	+	−	+
**Eriodictyol-7-rhamnopyranoside**	Good (−3.51)	Low (−0.46)	+	UGT-Centered (1A1/1A10)	+	±	−
**Fisetin-4-O-glucoside**	Good (−2.83)	Low (0.27)	+	UGT-Centered (1A1/1A10)	+	−	+
**Isoquercitrin**	Good (−3.35)	Low (0.24)	+	UGT-Centered (1A1/1A10)	+	±	+
**Kaempferol-4′-rhamnopyranoside**	Good (−3.60)	Low (0.20)	+	UGT-Centered (1A1/1A10)	+	±	+
**Nirurin**	Good (−3.60)	Low (0.18)	+	UGT-Centered (1A1/1A9)	+	+	+
**Rutin**	Good (−3.30)	Very Low (−0.95)	+	UGT-Centered (1A1/1A10)	+	±	+

**Footnotes:** ^a^ Solubility (Log S): Consensus from SwissADME (ESOL). Good > −4; Moderate −4 to −6; Poor < −6. ^b^ Permeability (Caco-2): Predicted by pkCSM (log P~app~, cm/s). High > 0.9; Moderate 0.3–0.9; Low 0–0.3; Very Low < 0. ^c^ Metabolic Pathway: Inferred from Way2Drug SOMP/SMP. ^d^ CYP3A4-Dependent: >80% metabolism via CYP3A4 (high DDI risk). CYP-Diversified: metabolism distributed across multiple CYP isoforms. UGT-Centered: predominant Phase II glucuronidation (low DDI risk). ^e^ Hepatotoxicity: Integrated assessment from pkCSM, Deep-PK (DILI I/II), and ADVERPred. ^f^ Cardiotoxicity: Integrated assessment of hERG inhibition (pkCSM/Deep-PK) and arrhythmia/cardiac failure risk (ADVERPred).

**Table 2:** Comparative docking estimated binding affinities (ΔG, kcal/mol) for phytochemical candidates and controls against T790M EGFR (PDB: 7A6K) and wild-type EGFR (PDB: 1M17). Contacts = number of interacting residues. ΔΔG > 0 indicates computationally predicted mutant preference.

Compound	ΔG T790M (kcal/mol)	ΔG WT (kcal/mol)	ΔΔG (kcal/mol)	Contacts T790M	Contacts WT
Corilagin	−8.677	−8.4	+0.28	18	36
Eriodictyol-7-rhamnopyranoside	−8.561	−9.7	−1.14	17	37
Phyltetralin	−8.507	−8.0	+0.51	20	28
Osimertinib	−9.737	−8.0	+1.74	19	38

Footnote: ΔΔG = ΔG(T790M) − ΔG(WT); positive values indicate computationally predicted mutant preference at the static docking level.

**Table 3:** **MM-PBSA-derived ΔΔG_bind_ values and in silico-estimated selectivity indices (IS) for T790M versus wild-type EGFR.** IS = |ΔGbind T790M|/|ΔGbind WT|. Osimertinib serves as internal reference (IS = 1.43; IC50 ~1 nM T790M vs. ~182 nM WT).

Compound	ΔGbind WT (kcal/mol)	ΔGbind T790M (kcal/mol)	ΔΔGbind (kcal/mol)	IS (T790M/WT)	Interpretation
**Corilagin ★**	−19.49 ± 2.79	−53.71 ± 5.05	+34.22	2.76 ★	Highest IS; 1.93× reference
**Eriodictyol-7-rhamnopyranoside**	−37.83 ± 2.16	−44.35 ± 4.51	+6.52	1.17	Mutant-preferential
**Phyltetralin**	−28.57 ± 3.97	−34.54 ± 4.01	+5.97	1.21	Mutant-preferential
**Osimertinib (reference)**	−18.00 ± 3.21	−25.74 ± 3.37	+7.74	1.43	Internal benchmark

Footnote: IS = |ΔG_bind_ (T790M)|/|ΔG_bind_ (WT)|; IS > 1.0 indicates computationally predicted mutant preference. ★ Corilagin IS of 2.76 exceeds the osimertinib reference (IS = 1.43) by a factor of 1.93 (MM-PBSA-derived).

**Table 4:** **PCA variance decomposition and cluster quality by pharmacological class**. PC1 is dominated by ΔGGAS in all four classes, reflecting its numerical magnitude across the dataset rather than pharmacological discriminance. ΔEGB loads negatively (−0.402 to −0.425), anticorrelated with ΔGGAS, consistent with MM-PBSA energy decomposition thermodynamics.

Class	PC1 (%Var)	PC2 (%Var)	PC1 + PC2	PC1 Dominant Loading	PCA Silhouette
**Flavonoids**	76.1%	17.7%	93.8%	ΔGGAS (+0.430)	0.062 (near-random)
**Lignans**	67.1%	28.2%	95.4%	ΔGGAS (+0.458)	0.008 (random)
**Sec. Metabolites**	81.4%	13.5%	94.9%	ΔGGAS (+0.416)	0.325 (moderate)
**Controls**	65.2%	30.1%	95.3%	ΔGGAS (+0.464)	0.344 (moderate)

**Table 5:** **Per-class PCA versus LDA cluster quality metrics.** Silh. = Silhouette coefficient; C-H = Calinski–Harabasz index; D-B = Davies–Bouldin index; Sep. = LDA separability percentage. Flavonoids: Davies–Bouldin is the sole metric that worsens under LDA (−5.7%), a biologically coherent finding reflecting the energetic degeneracy of the 2-phenylchromone scaffold class rather than a methodological limitation.

Class	n	PCA Silh.	LDA Silh.	ΔLDA%	PCA C-H	LDA C-H	PCA D-B	LDA D-B	LDA Sep.%	LDA LD1 Top
**Flavonoids**	1200	0.062	0.088	+41.7%	387.1	632.7	2.718	2.882	93.3%	ΔGSOLV
**Lignans**	1000	0.008	0.055	+572%	191.5	223.8	2.195	1.831	86.0%	ΔGSOLV
**Sec. Met.**	600	0.325	0.461	+41.6%	573.4	982.2	1.098	0.871	100.0%	ΔEEL
**Controls**	302	0.344	0.695	+101.8%	161.8	1231.4	1.189	0.383	100.0%	ΔGSOLV

**Table 6:** **Global CDFT descriptors computed via Koopmans approximation at B3LYP-D3(BJ)/def2-TZVP.** ^a^ Two low-frequency imaginary modes detected; electronic properties remain valid (Section 2.8.1). IP_K_ = ionization potential; EA_K_ = electron affinity; η = chemical hardness; μ = chemical potential; ω = electrophilicity index; S = global softness; *N* = Parr nucleophilicity index.

Compound	IP_K_ (eV)	EA_K_ (eV)	η_K_ (eV)	μ_K_ (eV)	ω_K_ (eV)	S_K_ (eV^−1^)	N_Parr_ (eV)
**Corilagin**	6.168	1.623	2.273	−3.896	3.339	0.220	2.953
**Eriodictyol-7-rhp** **^a^**	6.020	1.780	2.120	−3.900	3.587	0.236	3.101
**Osimertinib**	5.226	1.419	1.904	−3.323	2.965	0.263	3.895
**Phyltetralin**	5.351	0.328	2.512	−2.839	1.605	0.199	3.770
**Rutin**	5.877	1.868	2.004	−3.873	3.741	0.250	3.244

**Table 7:** **MEP surface parameters at B3LYP-D3(BJ)/def2-TZVP/RIJCOSX.** σ^2^_tot_ = overall ESP variance (kcal/mol)^2^; ν = balance of charges; MPI = molecular polarity index; PSA-DFT = polar surface area (|ESP| > 10 kcal/mol). ^a^ Electronic properties valid despite two imaginary vibrational modes.

Compound	V_s,min_ (kcal/mol)	V_s,max_ (kcal/mol)	σ ^2^_tot_ ((kcal/mol)^2^)	ν	MPI (eV)	PSA-DFT (Å ^2^ )	μ_dip_ (D)
**Corilagin**	−46.92	+69.78	392.04	0.231	0.753	303.08	7.246
**Corilagin**	−40.37	+70.17	286.20	0.221	0.681	265.84	7.307
**Eriodictyol-7-rhamnopyranoside** **^a^**	−46.40	+37.91	195.06	0.219	0.701	366.99	9.678
**Osimertinib**	−39.65	+20.76	100.07	0.161	0.377	154.57	2.689
**Phyltetralin**	−43.45	+69.11	271.94	0.227	0.628	271.70	3.326

## Data Availability

All data, analysis results, and input files supporting the findings of this study are publicly available in a permanent Zenodo repository at the following digital object identifier (DOI): https://doi.org/10.5281/zenodo.18614515. The deposited data package is organized into the following main folders, each containing a specific README.txt file for detailed explanations: Protein Structures—Initial and optimized coordinates of EGFR T790M/V948R (PDB 7A6K) and EGFR wild-type (PDB 1M17) used for docking and molecular dynamics simulations. Ligand Structures—SMILES strings, 3D conformers, and force-field parameters for the 37 *Phyllanthus niruri* phytochemicals and two clinical reference compounds (osimertinib, mobocertinib). Molecular Docking—Raw and processed output files from SwissDock 2024 for all 37 phytochemicals against EGFR T790M, plus comparative docking against wild-type EGFR for the four lead candidates. ADMET and Bioactivity Predictions—Complete outputs from SwissADME, pkCSM, Deep-PK, PASS Online, and KinScreen platforms. Molecular Dynamics Simulations—GROMACS input files (.mdp, topology, index), compressed trajectories (.xtc), and processed analyses (RMSD, RMSF, Rg, SASA, hydrogen bonds) for all 16 T790M complexes (100 ns each) and all five wild-type EGFR complexes (100 ns each). MM-PBSA Binding Free Energies and Per-Residue Decomposition—gmx_MMPBSA input files, raw energy component data (7416 frames for PCA/LDA; 1,548,639 data points for per-residue decomposition), bootstrap confidence intervals, and Kruskal–Wallis statistical test outputs. PCA/LDA Multivariate Analysis—Python scripts (scikit-learn), input CSV matrices, and complete output tables (PCA loadings, LDA coefficients, Silhouette/Calinski–Harabasz/Davies–Bouldin metrics). DFT Electronic Structure Characterization—ORCA 6.1 input and output files for geometry optimization and frequency calculations at B3LYP-D3(BJ)/def2-TZVP level; Multiwfn 3.8 input files and complete surfanalysis outputs (MEP surfaces, Fukui functions, dual descriptor, Hirshfeld orbital composition, NCI/RDG) for the five top-ranked compounds. All software and web servers used in this study are publicly available and detailed in the Methodology section and the repository’s README files.

## Supplementary Materials

The following supporting information can be downloaded at https://www.mdpi.com/article/10.3390/ijms27156568/s1.

## Abbreviations

The following abbreviations are used in this manuscript:

**Abbreviation**	**Definition**
ADMET	Absorption, Distribution, Metabolism, Excretion, and Toxicity
AMES	Ames mutagenicity test
ATP	Adenosine Triphosphate
BBB	Blood–Brain Barrier
CA	Carbonic Anhydrase
Caco-2	Human colorectal adenocarcinoma cell line (permeability model)
CDD	Dual Descriptor
CDFT	Conceptual Density Functional Theory
CYP	Cytochrome P450
DDI	Drug–Drug Interaction
DFT	Density Functional Theory
DILI	Drug-Induced Liver Injury
DUD-E	Directory of Useful Decoys Enhanced
DYRK2	Dual-specificity tyrosine phosphorylation-regulated kinase 2
EA	Electron Affinity
EGFR	Epidermal Growth Factor Receptor
HIA	Human Intestinal Absorption
hERG	human Ether-à-go-go-Related Gene
HOMO	Highest Occupied Molecular Orbital
HCC	Hepatocellular Carcinoma
IC_50_	Half-maximal Inhibitory Concentration
IP	Ionization Potential
IS	Selectivity Index
KW	Kruskal–Wallis
LDA	Linear Discriminant Analysis
LINCS	Linear Constraint Solver
LUMO	Lowest Unoccupied Molecular Orbital
MD	Molecular Dynamics
MEP	Molecular Electrostatic Potential
MMFF94	Merck Molecular Force Field 94
MM-GBSA	Molecular Mechanics Generalized Born Surface Area
MM-PBSA	Molecular Mechanics Poisson–Boltzmann Surface Area
MPI	Molecular Polarity Index
NCI	Non-Covalent Interactions
NEK6	NIMA-related kinase 6
NPT	Isothermal–isobaric ensemble
NVT	Canonical ensemble
ODI	Orbital Delocalization Index
Pa	Probability of Activity
PASS	Prediction of Activity Spectra for Substances
PCA	Principal Component Analysis
PDB	Protein Data Bank
P-gp	P-glycoprotein
Pi	Probability of Inactivity
PME	Particle Mesh Ewald
PSA	Polar Surface Area
QM/MM	Quantum Mechanics/Molecular Mechanics
RDG	Reduced Density Gradient
Rg	Radius of Gyration
RMSD	Root Mean Square Deviation
RMSF	Root Mean Square Fluctuation
ROC-AUC	Receiver Operating Characteristic Area Under the Curve
SAR	Structure–Activity Relationship
SASA	Solvent Accessible Surface Area
SI	Selectivity Index
SMD	Solvation Model based on Density
SMILES	Simplified Molecular Input Line Entry System
T790M	Threonine 790 to Methionine mutation
TAK-788	Mobocertinib (EGFR/HER2 inhibitor)
UGT	UDP-glucuronosyltransferase
V948R	Valine 948 to Arginine mutation
WT	Wild-Type
