# Supplementary material for: Mutant-Selective Binding of Phyllanthus niruri Phytochemicals to EGFR T790M: A Quantum-Classical Mechanistic Study
Source: Int J Mol Sci. 2026 Jul 23;27(15):6568. doi: 10.3390/ijms27156568 (PMC13465364; doi:10.3390/ijms27156568)
Supplement: Supplementary file 1 [file ijms-27-06568-s001.zip › ijms-4182232-supplementary.pdf]

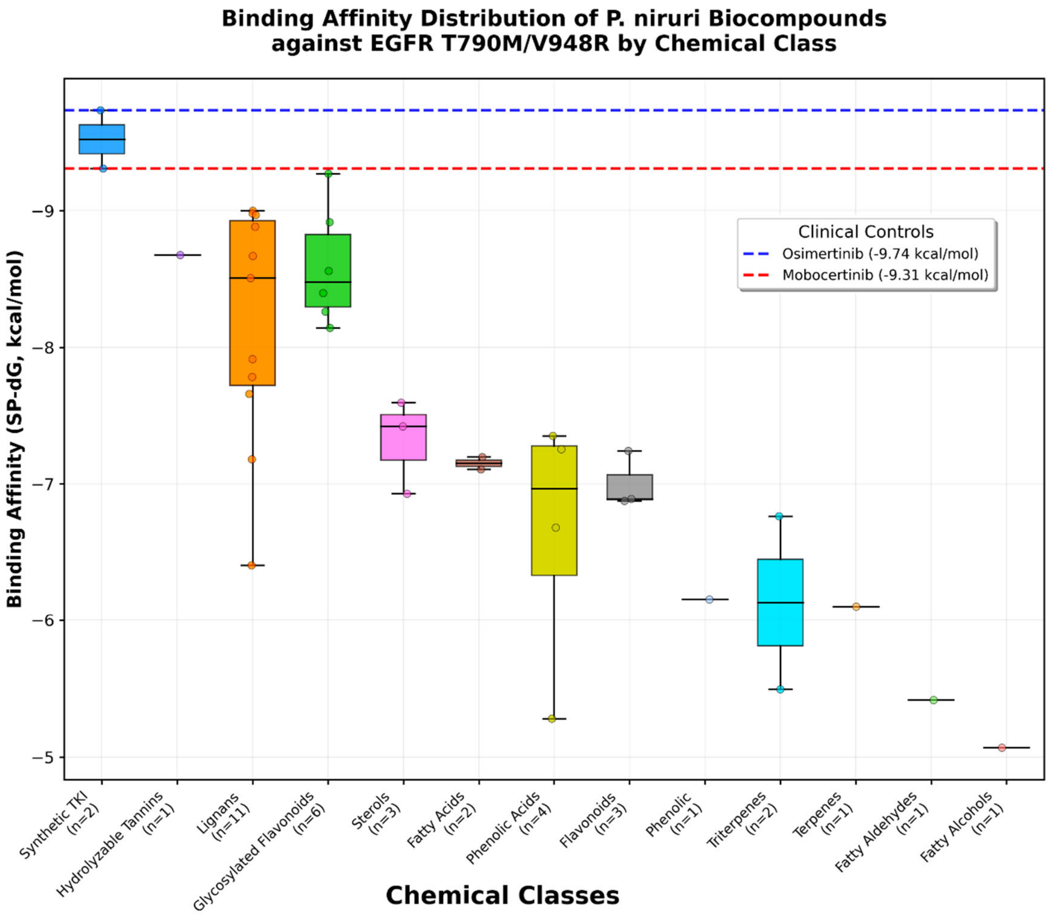

Supplementary Figure S1. Binding Affinity Distribution of *P. niruri* Biocompounds Stratified by Chemical Class Reveals High-Potential Scaffolds. Box-and-whisker plot displaying the distribution of binding affinity (SwissDock SP-ΔG, kcal/mol) for 37 *P. niruri* phytochemicals stratified by chemical class. Horizontal reference lines indicate clinical control affinities: osimertinib (−9.74 kcal/mol, blue dashed line) and mobocertinib (−9.31 kcal/mol, red dashed line). Lignans (n=11) and glycosylated flavonoids (n=6) emerge as high-potential classes, with median binding affinities and interquartile ranges competing with or exceeding clinical standards. In marked contrast, phenolic acids and terpenes display substantially weaker affinities. This class-stratified analysis justified selection of 14 top-ranked candidates (drawn from high-performance classes) for downstream molecular dynamics validation. Box shows interquartile range; central line indicates median; individual points represent per-compound values. Error bars span ±1 SD from triplicate docking submissions.

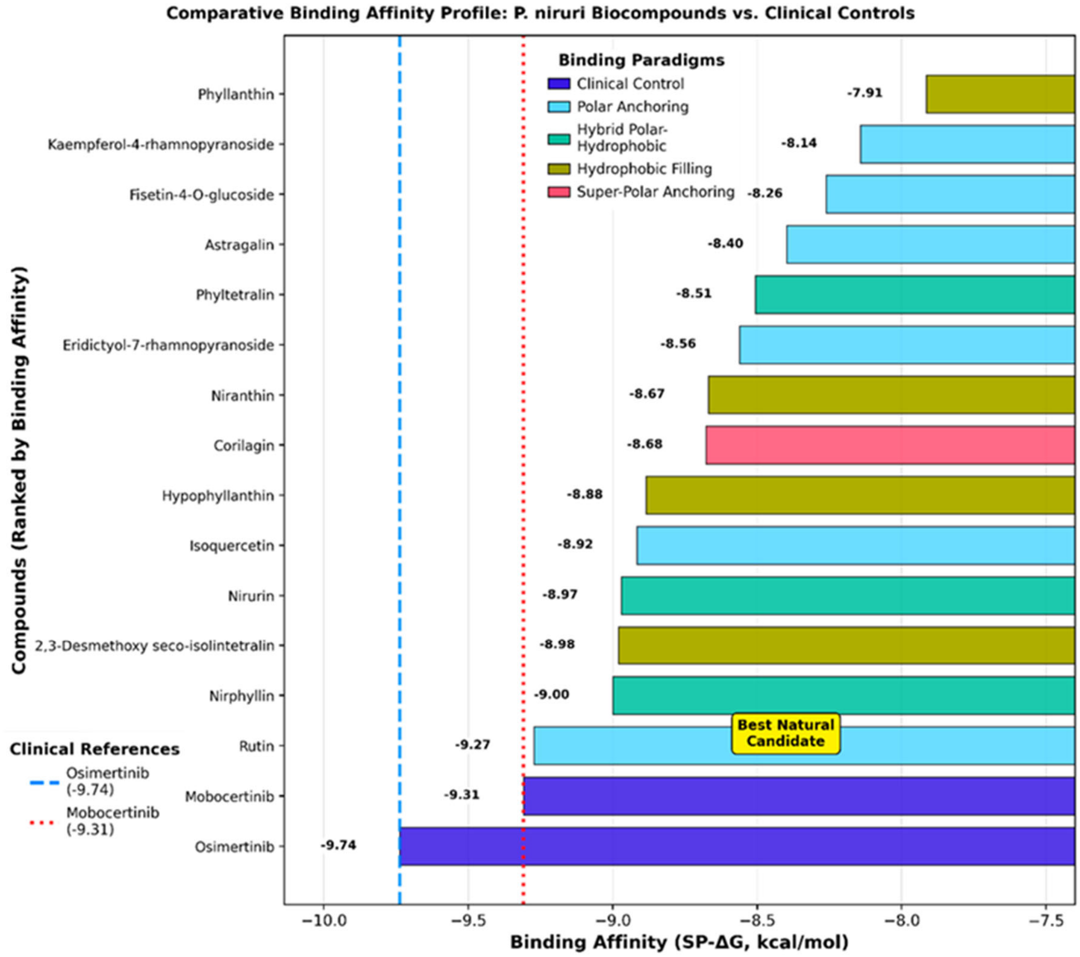

Supplementary Figure S2. Docking Score Distribution for 37 *P. niruri* Phytochemicals and Clinical Reference Compounds Against EGFR T790M (PDB 7A6K). Horizontal bar chart displaying SwissDock ΔG values for the 14 selected phytochemicals and clinical controls osimertinib (−9.74 kcal/mol) and mobocertinib (−9.31 kcal/mol). Horizontal dashed lines indicate the reference scores of osimertinib (blue) and mobocertinib (red). Color-coding indicates compound class: flavonoid glycosides (light blue), hydrolysable tannins (magenta), lignans (olive), and mixed polyphenols (teal). The dashed vertical line at ΔG = −7.91 kcal/mol indicates the selection threshold (95th percentile of ChEMBL EGFR actives; Supplementary Figure S1). All values from triplicate docking runs; error bars represent ±1 SD.

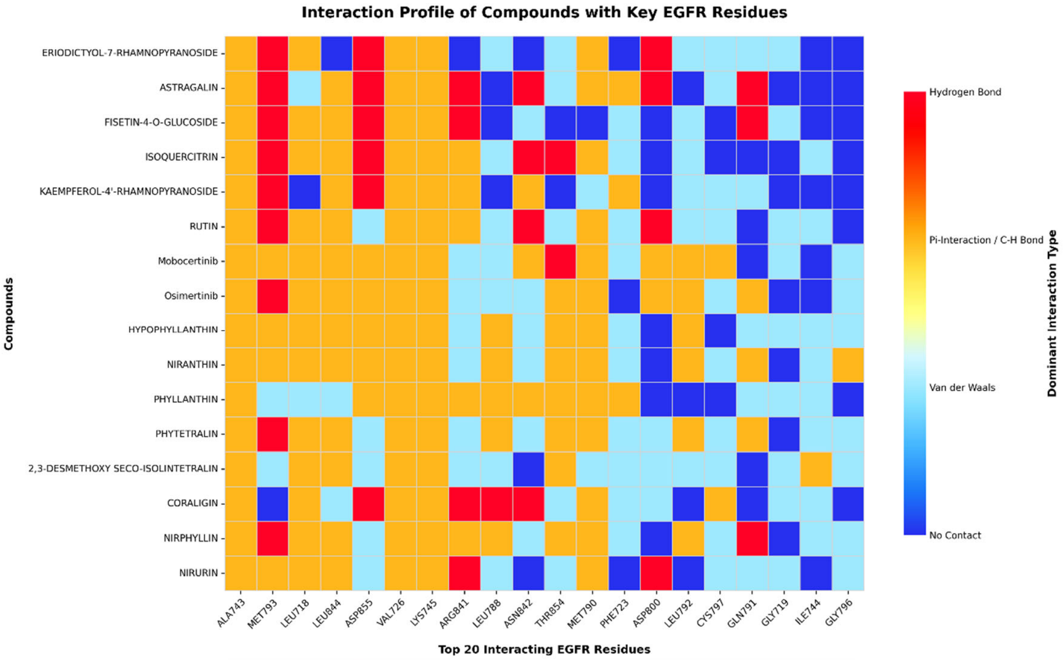

Supplementary Figure S3. Interaction Profile Heatmap Reveals Residue-Specific Engagement Patterns Distinguishing Binding Paradigms. Hierarchical interaction heatmap displaying the dominant interaction type (hydrogen bond, π-interaction/C-H bond, van der Waals, no contact) between 14 top-ranked *P. niruri* phytochemicals and 2 clinical controls with the 20 residues selected as those showing ≥50% contact frequency across all compounds contacted EGFR T790M/V948R binding-site residues. Color intensity indicates interaction prevalence: red (hydrogen bond), orange (π-interaction), light blue (van der Waals), dark blue (no contact). Row-wise hierarchical clustering segregates compounds into mechanistically distinct interaction patterns corresponding to binding paradigm classification. Polar-anchoring compounds (rutin, isoquercitrin, astragalin, eriodictyol-7-rhamnopyranoside) show intensive red/orange engagement at hinge-region residues (Met793, Asp800, Asn842), with minimal hydrophobic contact. Hydrophobic-filling lignans (nirphyllin, hypophyllanthin, phyllanthin) display dominant yellow/light-blue coloration at gatekeeper-pocket residues (Leu718, Val726, Met790, Val948), with sparse polar interactions. Clinical controls exhibit hybrid profiles with diffuse engagement across all residues and reduced spatial clustering, indicating less specialized recognition. This residue-contact stratification quantitatively validates the mechanistically distinct paradigms inferred from binding mode analysis and supports structure-activity relationship optimization pathways for each compound class.

Supplementary Table S1. Binding energies and key interactions for selected *P. niruri* compounds.

Rank	Compound (Class)	SP-dG (kcal mol⁻¹)	Key H-bond Anchors*	Predominant Hydrophobic Contacts†
-	Osimertinib (control)	–9.74	Met793 (×2)	Leu718, Val726, Met790
-	Mobocertinib (control)	–9.31	Thr854	Leu718, Val726, Met790
1	Rutin (flavonol-glycoside)	–9.27	Met793, Asp800, Asn842	Leu718, Val726, Met790
2	Nirphyllin (lignan)	–9.00	Gln791, Met793	Leu718, Val726, Met790
3	2,3-Desmethoxy-seco-isolintetralin (lignan)	–8.98	–	Leu718, Val726, Met790
4	Nirurin (lignan)	–8.97	Asp800 (×2), Arg841	Val726, Met790, Leu844
5	Isoquercitrin (flavonol-glycoside)	–8.92	Met793, Asn842, Thr854	Leu718, Val726, Met790
6	Hypophyllanthin (lignan)	–8.88	–	Leu718, Val726, Met790
7	Eriodictyol-7-rhamnoside (flavanone-glycoside)	–8.56	Met793, Asp800 (×2)	Leu718, Val726, Met790
8	Phytetralin (lignan)	–8.51	Met793	Leu718, Val726, Met790
9	2,3-Desmethoxy-phytetralin (lignan)	–8.50	–	Leu718, Val726, Met790
10	Astragalin (flavonol-glycoside)	–8.40	Gln791, Met793, Asp800	Val726, Met790, Leu844
11	Fisetin-4-O-glucoside (flavonol-glycoside)	–8.26	Gln791, Met793, Asp837	Leu718, Val726, Met790
12	Kaempferol-4′-rhamnoside (flavonol-glycoside)	–8.14	Met793, Asp837	Val726, Met790, Leu844
13	Niranthin (lignan)	–8.07	–	Leu718, Val726, Met790
14	Phyllanthin (lignan)	–7.91	–	Leu718, Val726, Met790

* Hydrogen bonds defined by D–A ≤ 0.32 nm, D–H···A ≥ 155°.

† Hydrophobic contacts include van der Waals, alkyl and π-alkyl interactions inside the enlarged T790M pocket.

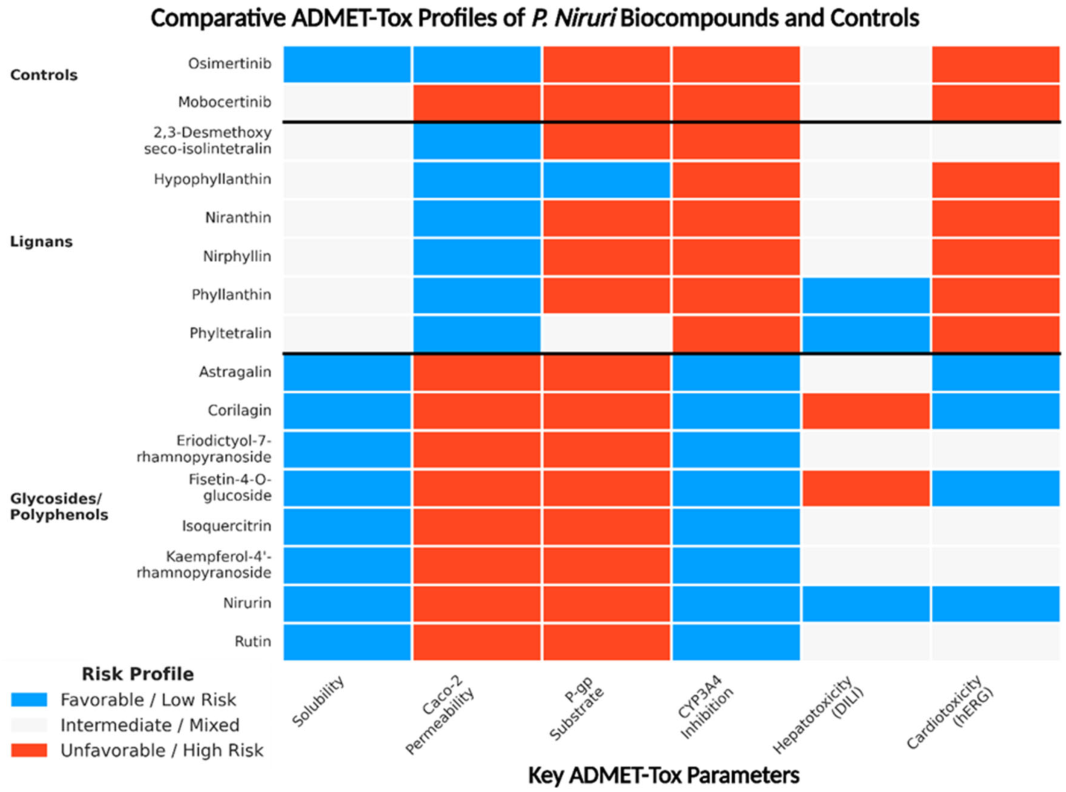

Supplementary Figure S4. Comparative ADMET Risk Assessment for 14 *P. niruri* Phytochemicals and Clinical Reference Compounds. Heatmap displaying predicted risk categories based on consensus predictions from SwissADME, pkCSM, and Deep-PK. Rows: individual compounds grouped by structural class (lignans, glycosides, polyphenols, clinical controls). Columns: predicted endpoints (Caco-2 permeability, CYP3A4 inhibition, hERG inhibition, hepatotoxicity). Color code: blue = predicted low risk (Pa < 0.30); gray = predicted intermediate risk or conflicting predictions between platforms (Pa 0.30–0.70); red = predicted high risk (Pa > 0.70). All values are in silico predictions; no experimental pharmacokinetic data were obtained in this study.

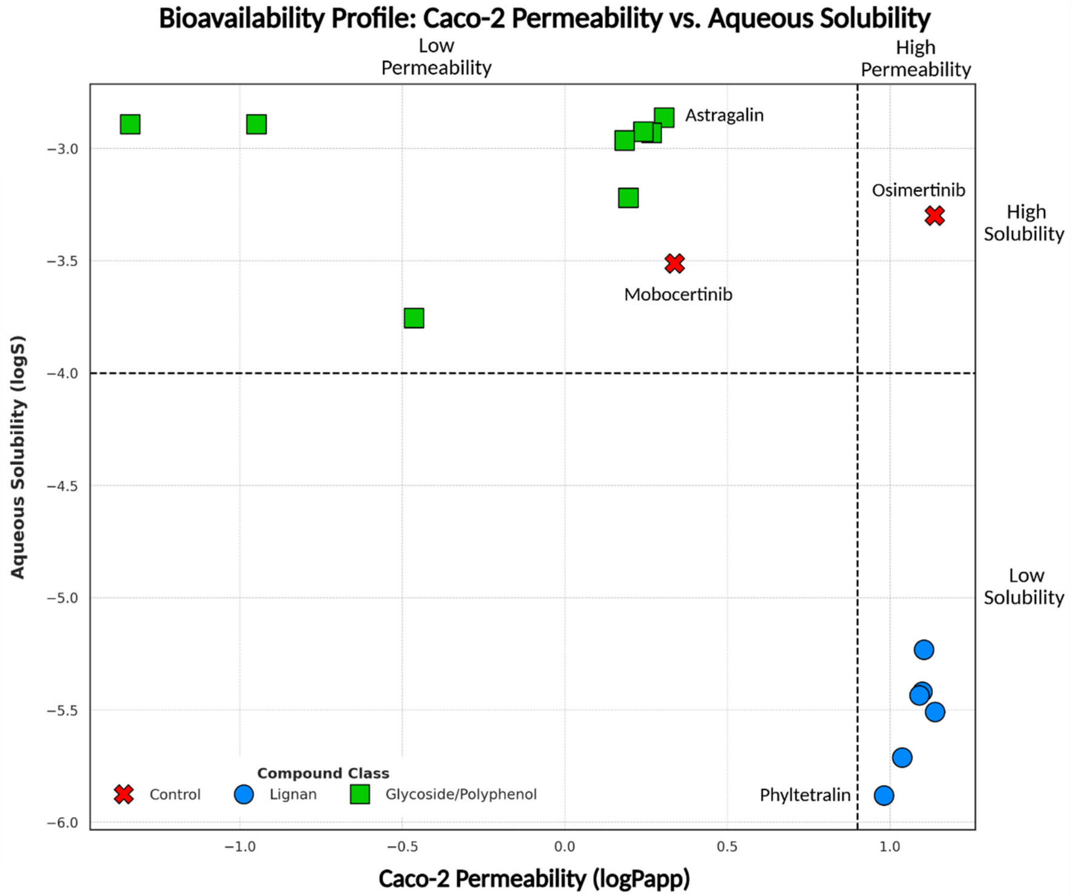

Supplementary Figure S5. Pharmacokinetic bifurcation: Caco-2 permeability vs. aqueous solubility reveals distinct absorption challenges for lignans and glycosides. Scatter plot visualization of predicted oral bioavailability potential for 14 *P. niruri* biocompounds and 2 clinical controls. Data points represent pkCSM-predicted Caco-2 permeability (logPapp, x-axis) versus aqueous solubility (logS, y-axis). Dashed lines indicate standardized thresholds for high permeability (>0.9 logPapp) and high solubility (>-4.0 logS). (1) "Solubility-Limited Zone" (Bottom-Right): Lignans (e.g., hypophyllanthin, phyllanthin) cluster with high permeability comparable to osimertinib but low aqueous solubility, suggesting bioavailability is dissolution-rate limited and amenable to lipid-based formulation. (2) "Permeability-Limited Zone" (Top-Left): Glycosides (e.g., astragalin, fisetin-4-O-glucoside) exhibit high solubility but poor intestinal permeability, indicating uptake barriers requiring permeation enhancers or alternative delivery routes. (3) "Ideal Zone" (Top-Right): Occupied exclusively by the control osimertinib, reflecting optimized drug-likeness. (4) "Dual Challenge" (Bottom-Left): Mobocertinib and select large polyphenols (corilagin, rutin) face both dissolution and permeation hurdles. This plot confirms a fundamental pharmacokinetic divergence: lignans are permeability leaders requiring solubility optimization, while glycosides are safety leaders requiring permeability enhancement.

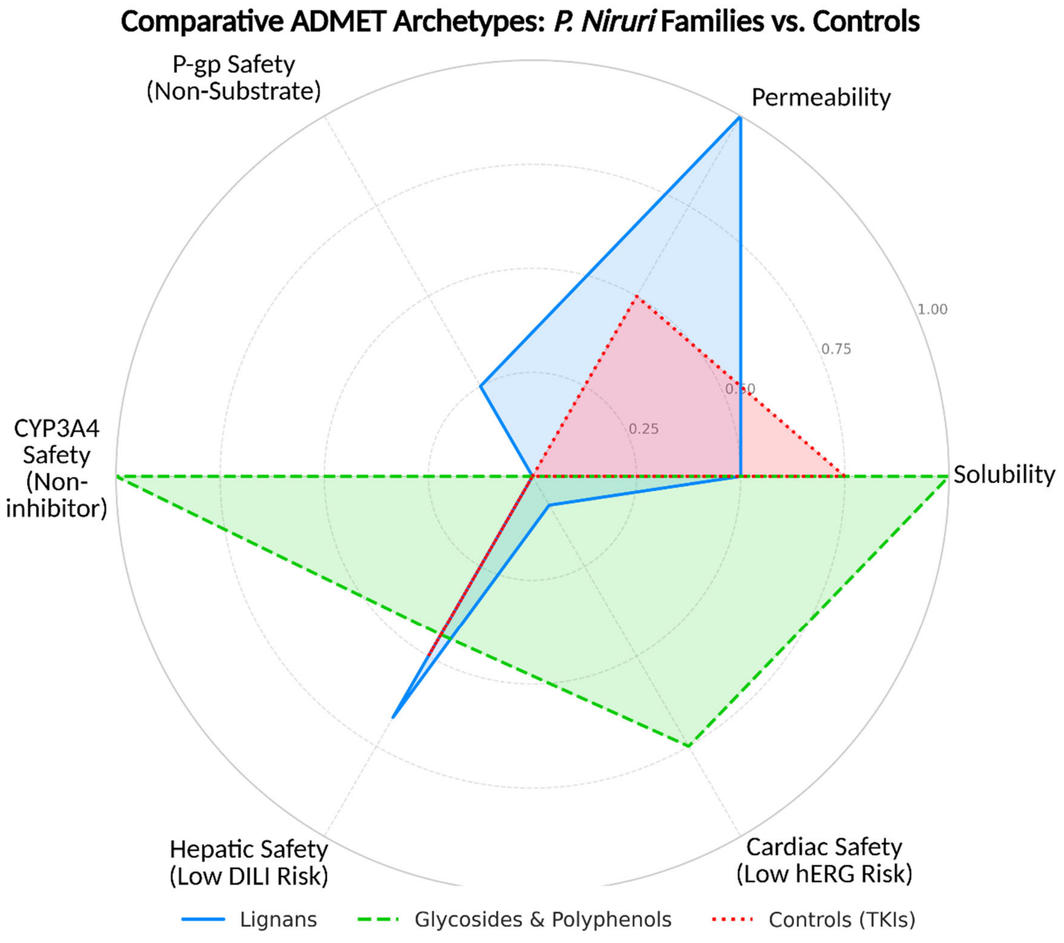

Supplementary Figure S6. Integrated ADMET radar profiles define distinct safety-efficacy archetypes for *P. niruri* biocompounds versus clinical controls. Normalized multi-dimensional comparison of aggregated safety profiles across three chemical classes (n=16). Radial axes represent key pharmacokinetic/toxicological parameters scaled from 0 (high risk) to 1.0 (optimal safety). (1) Clinical Controls (Red): Exhibit a contracted polygon reflecting multifactorial liabilities, including high CYP3A4 inhibition (0.0) and cardiotoxicity (0.0), despite acceptable solubility. (2) Permeability Leaders (Blue/Lignans): Maximize intestinal absorption potential (Permeability 1.0) but are compromised by CYP3A4 inhibition (0.0) and significant cardiotoxicity risks (0.08; corrected v2.0 data), necessitating careful monitoring. (3) Safety Leaders (Green/Glycosides): Display an inverted profile prioritizing metabolic safety (CYP3A4 Safety 1.0) and favorable cardiac profiles (0.75), but are severely limited by poor membrane permeability (0.0). This visualization confirms a strategic trade-off: lignans offer systemic exposure, while glycosides offer a clean metabolic profile ideal for polypharmacy in cirrhotic patients. Values normalized via (x+1)/2 transformation.

Supplementary Table S2. Metabolic safety profiling reveals distinct detoxification strategies for *P. niruri* phytochemicals versus CYP-dependent clinical controls. Comparative analysis of hepatic clearance pathways, drug-drug interaction (DDI) liabilities, and hepatoprotective mechanisms. Classification: Compounds are stratified into three metabolic archetypes based on SOMP/SMP predictions (Way2Drug). (1) Clinical Controls: Exhibit high dependence on oxidative Phase I metabolism (CYP450), resulting in significant DDI risks and reduced stability in cirrhotic tissues where CYP activity is compromised. (2) Group I (Safety Leaders): Glycosides utilize a UGT-centered (Phase II) detoxification strategy, effectively bypassing the CYP3A4 bottleneck and minimizing interaction risks. (3) Group II (Permeability Leaders): Lignans employ a CYP-diversified strategy (relying on isoforms 2C9, 2C8, 2B6 rather than 3A4), offering improved stability over controls. Scores: Values in parentheses represent normalized risk/stability scores (0–1 scale) derived from integrated in silico profiling.

Strategic Group	Representative Compounds	Primary Metabolic Pathway	CYP3A4 DDI Risk	Cirrhosis Stability	Hepatoprotective Mechanism
**Clinical Controls**	Osimertinib, Mobocertinib	**CYP-Centric** (Dependence: 3A4, 1A2, 2D6)	**Moderate-High** (Score: 0.40–0.90)	**Poor** (Score: 0.25–0.35)	**Minimal** (Score: 0.10–0.15)
**Group I: UGT-Centered *(Glycosides & Polyphenols)***	Rutin, Astragalin, Isoquercitrin, Corilagin, Nirurin, Fisetin-4-O-G, etc.	**Phase II Dominant** (UGT1A1 > UGT1A10 > UGT1A9)	**Negligible** (Score: 0.05–0.10)	**Excellent** (Score: 0.85–0.95)	**Intensive Nrf2-ARE Activation** (Direct UGT upregulation)
**Group II: CYP-Diversified *(Lignans)***	Hypophyllanthin, Phyllanthin, Phyltetralin, Niranthin, Nirphyllin, DMSI	**Phase I Diversified** (CYP2C9 > 2C8 > 2B6) + Selective 3A4 Inhibition	**Low-Moderate** (Score: 0.25–0.70)	**Good** (Score: 0.65–0.80)	**Dual Action** (Enzyme modulation + Nrf2)

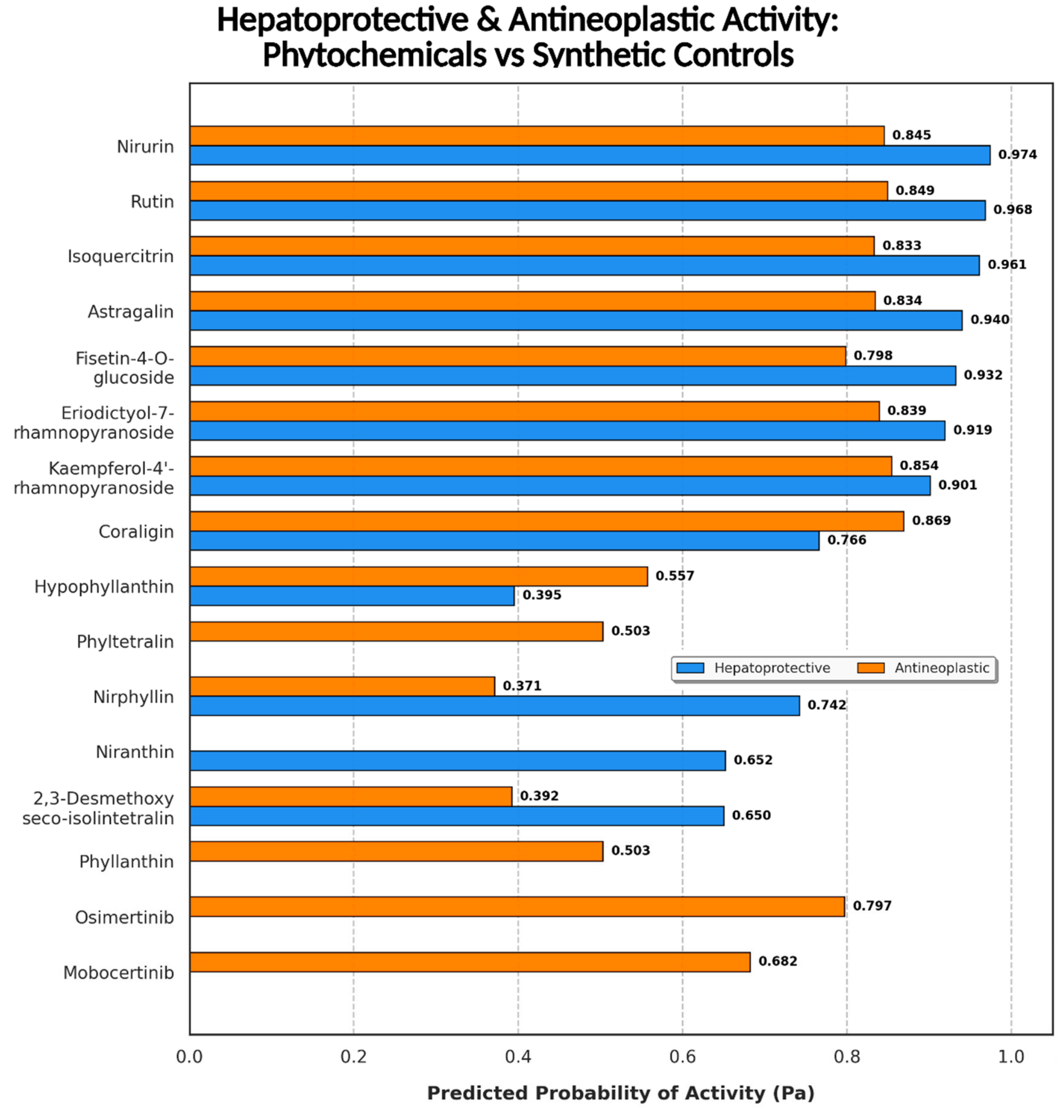

Supplementary Figure S7. Comparison of PASS-predicted Pa values for hepatoprotective and antineoplastic activity categories. Bar chart showing Pa values for hepatoprotection (blue) and antineoplastic activity (orange) for each compound. Top four phytochemicals by hepatoprotective Pa: nirurin (0.974), rutin (0.968), isoquercitrin (0.961), astragalin (0.940). Osimertinib and mobocertinib hepatoprotective Pa: <0.30 (below prediction threshold). Osimertinib antineoplastic Pa = 0.797; mobocertinib = 0.682. All values are probabilistic predictions from PASS Online (ChEMBL v.21); no experimental validation was performed.

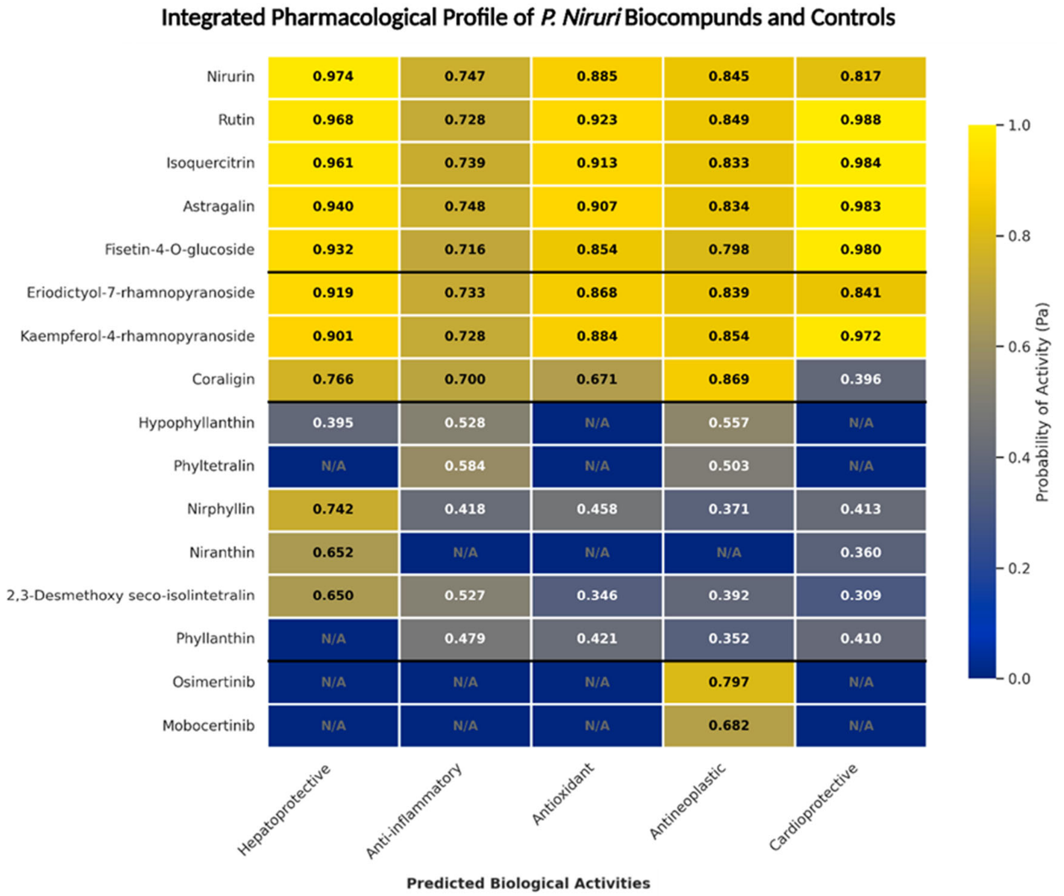

Supplementary Figure S8. PASS-predicted biological activity probabilities (Pa) for 14 *P. niruri* phytochemicals and clinical reference compounds. Heatmap of Pa values derived from PASS Online (ChEMBL v.21) for hepatoprotective, antineoplastic, cardioprotective, and CA isoform activity categories. Compounds are ordered by decreasing hepatoprotective Pa. Color scale: Pa from 0.0 (white) to 1.0 (dark blue). "N/A" indicates Pa < 0.30 (below the activity-prediction threshold used in this analysis). For lignans, antineoplastic Pa values reflect the highest prediction for any specific cancer subtype (lung or breast) where the generic antineoplastic category was not predicted (details in Supplementary Table S3). All values are probabilistic predictions; experimental validation was not performed.

Supplementary Table S3. Therapeutic context analysis: PASS-predicted activity profiles suggest P. niruri biocompounds may target critical HCC progression and resistance modules not covered by synthetic inhibitors. All values are probabilistic in silico predictions. Integration of high-confidence PASS Target predictions (Pa >0.6) with established hepatocellular carcinoma pathophysiology. Key Findings: Natural compounds demonstrate a "multi-dimensional" therapeutic efficacy, simultaneously attacking tumor survival mechanisms that are pharmacologically bypassed by synthetic kinase inhibitors. (1) Hypoxia: Flavonoids (e.g., isoquercitrin) potently inhibit Carbonic Anhydrases, critical for pH regulation in hypoxic tumors. (2) Metabolic Resistance: Lignans (e.g., niranthin) modulate CYP3A4/2C9, potentially reversing metabolic inactivation of co-administered drugs. (3) Hepatoprotection: Unique targeting of inflammatory mediators (Anaphylatoxin receptor) and cardiac proteins (Troponin) by astragalin and corilagin provides a mechanistic basis for organ protection. (4) Multidrug Resistance: Broad engagement of efflux pumps (P-gp, MRP1) by multiple natural scaffolds contrasts with the null profile of controls. *Note:* Synthetic controls are confirmed as exclusive kinase inhibitors, lacking activity in the microenvironment and metabolic modulation domains.

Therapeutic Context	Key Molecular Targets	Natural Compounds (Top Performers)	Synthetic Controls	HCC Therapeutic Relevance
**Hypoxia & pH Regulation**	CA III, CA IX, CA XII, CA XIV	**Isoquercitrin** (Pa 0.96) **Fisetin-4-O-glucoside** (Pa 0.94)	**No significant targets**	**High** Modulation of acidic tumor microenvironment
**Drug Metabolism & Resistance**	CYP3A4, CYP2C9, CYP2C19	**Niranthin** (Pa 0.62) **Nirphyllin** (Pa 0.61)	**No significant targets**	**High** Overcoming metabolic inactivation
**Inflammation & Hepatoprotection**	Anaphylatoxin Receptor, Cardiac Troponin	**Corilagin** (Pa 0.98) **Astragalin** (Pa 0.95)	**No significant targets**	**High** Liver protection & anti-inflammatory signaling
**Cell Cycle & Proliferation**	NEK6, NEK2, FYN, EGFR	**Kaempferol-4'-R** (Pa 0.77) **Astragalin** (Pa 0.72)	**High Activity** (HIPK4 0.84, NEK3 0.87)	**Moderate** General anticancer activity
**Multidrug Resistance**	P-gp, MRP1, ABCG2	**Rutin** (Pa 0.79) **Astragalin** (Pa 0.71) **Phyllanthin** (Pa 0.60)	**No significant targets**	**High** Enhancement of drug bioavailability

Supplementary Table S4. Inventory of high-confidence molecular targets reveals distinct polypharmacological mechanisms for *Phyllanthus niruri* phytochemicals versus synthetic EGFR inhibitors. Data represents the complete list of predicted compound-target interactions with high confidence probability (Pa ≥ 0.7), derived from PASS Online structure-activity relationship analysis (ChEMBL v.21). (1) Natural Compound Specificity: Flavonoid glycosides demonstrate a pharmacological signature defined by the targeting of Carbonic Anhydrases (CA III, IX, XIV) and NEK6 kinase, implicating them in the regulation of tumor pH and mitotic progression. Lignans exhibit a unified profile targeting the nuclear receptor NR0B1 and metabolic enzymes, supporting a mechanism of transcriptional and metabolic modulation. (2) Synthetic Control Specificity: In contrast, osimertinib and mobocertinib exhibit an exclusive "kinase-only" profile (targeting HIPK4, NEK3), validating their design as monofocal inhibitors but highlighting their lack of activity against microenvironmental or metabolic resistance targets. (3) Classification: "Direct" interactions indicate immediate molecular binding; "Probable" interactions suggest indirect or pathway-mediated effects. This dataset provides the granular evidence for the "Resistance Module Targeting" heatmap presented in Figure 5.

Compound Class	Bioactive Compound	Molecular Target	Confidence (Pa)	Interaction Type
Flavonoid Glycosides	**Isoquercitrin**	Carbonic anhydrase III	0.955	Direct
	**Fisetin-4-O-glucoside**	Carbonic anhydrase III	0.943	Direct
	**Astragalin**	Carbonic anhydrase III	0.938	Direct
		Carbonic anhydrase XIV	0.715	Direct
		Serine/threonine-protein kinase NEK6	0.722	Direct
	**Kaempferol-4'-rham.**	MDR-associated protein 1 (MRP1)	0.874	Probable
		Serine/threonine-protein kinase NEK6	0.773	Probable
		Tyrosine-protein kinase FYN	0.718	Probable
		Aldose reductase	0.706	Direct
	**Eriodictyol-7-rham.**	MDR-associated protein 1 (MRP1)	0.867	Probable
		Tyrosine-protein kinase FYN	0.744	Probable
	**Rutin**	MDR-associated protein 1 (MRP1)	0.787	Probable
		Tyrosine-protein kinase FYN	0.778	Probable
		Carbonic anhydrase IX	0.732	Direct
		Ribosomal protein S6 kinase alpha 3	0.712	Probable
		Serine/threonine-protein kinase NEK6	0.701	Direct
Lignans	**2,3-Desmethoxy...**	Nuclear receptor subfamily 0 group B member 1	0.818	Probable
	**Phyltetralin**	Nuclear receptor subfamily 0 group B member 1	0.789	Probable
	**Niranthin**	Nuclear receptor subfamily 0 group B member 1	0.786	Probable
		Sphingomyelin phosphodiesterase	0.728	Probable
	**Nirphyllin**	Nuclear receptor subfamily 0 group B member 1	0.776	Probable
	**Hypophyllanthin**	Nuclear receptor subfamily 0 group B member 1	0.770	Probable
	**Phyllanthin**	Nuclear receptor subfamily 0 group B member 1	0.762	Probable
**Ellagitannins**	**Corilagin**	Troponin cardiac muscle	0.982	Probable
**Specialized**	**Nirurin**	Protein-tyrosine phosphatase 1B	0.706	Direct
**Synthetic Controls**	**Osimertinib**	Homeodomain-interacting protein kinase 4	0.875	Direct
		Serine/threonine-protein kinase NEK3	0.868	Direct
	**Mobocertinib**	Homeodomain-interacting protein kinase 4	0.811	Direct
		Serine/threonine-protein kinase NEK3	0.748	Direct

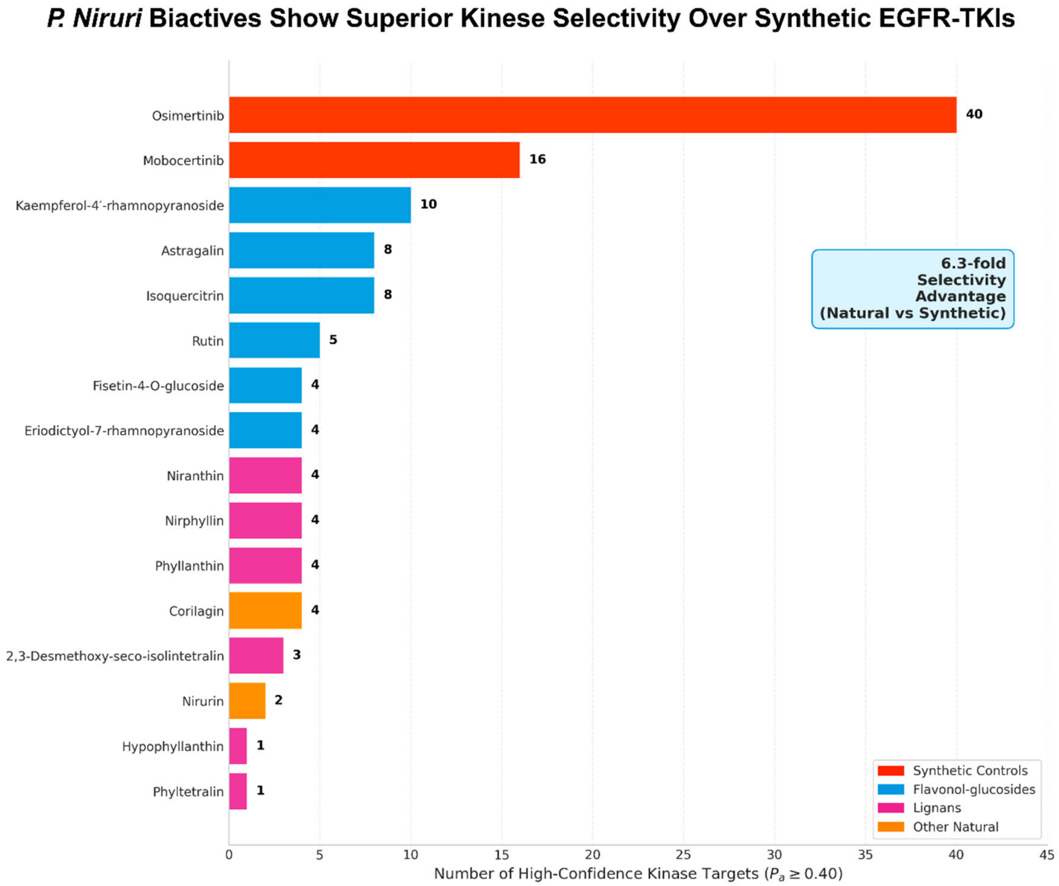

Supplementary Figure S9. Number of predicted high-confidence kinase interactions (Pa ≥ 0.40) per compound from KinScreen analysis. Bar chart showing predicted kinase interaction counts for 14 phytochemicals and two clinical controls (osimertinib: 40; mobocertinib: 16). Mean phytochemicals: 3.5 ± 2.1; mean controls: 28.0 ± 12.0; ratio: 8.0-fold. Compounds stratified by class (flavonoid glycosides, lignans). All values are computational predictions; experimental kinase profiling was not performed.

Supplementary Table S5. Backbone RMSD statistical analysis for EGFR T790M/V948R molecular dynamics simulations. Structural stability metrics extracted from 100-ns production trajectories for 14 *P. niruri* biocompounds and 2 clinical controls. Rank: Compounds ordered by mean RMSD (ascending, most stable to least stable). Mean RMSD: Time-averaged backbone $C\alpha\alpha$ deviation. SD: Standard deviation of RMSD distribution. CV: Coefficient of variation (SD/Mean), quantifying relative dispersion. τ: Integrated autocorrelation time, indicating conformational relaxation timescale and sampling efficiency (lower values = faster convergence). Class: Phytochemical classification. Glycosides (n=7) exhibit superior structural rigidity (mean RMSD = 2.57 ± 0.28 Å) compared to lignans (n=6, mean RMSD = 3.06 ± 0.40 Å) and clinical controls (n=2, mean RMSD = 2.87 ± 0.31 Å). Corilagin (tannin) demonstrates low RMSD (2.56 Å) but prolonged equilibration (τ = 13.77 ns), reflecting complex induced-fit dynamics. All simulations performed under identical protocols (NPT ensemble, 310 K, explicit TIP3P solvent).

Rank	Compound	Class	Mean RMSD (Å)	SD (Å)	CV	τ (ns)
**1**	Eriodictyol-7-rhamnopyranoside	Glycoside	2.162	0.251	0.116	3.54
**2**	Kaempferol 4'-rhamnoside	Glycoside	2.214	0.244	0.110	4.32
**3**	Hypophyllanthin	Lignan	2.499	0.415	0.166	12.17
**4**	Corilagin	Tannin	2.560	0.452	0.177	13.77
**5**	Isoquercitrin	Glycoside	2.611	0.248	0.095	1.97
**6**	Mobocertinib	Control	2.659	0.341	0.128	9.96
**7**	Fisetin-4-o-glucoside	Glycoside	2.664	0.348	0.131	4.47
**8**	Astragalin	Glycoside	2.672	0.331	0.124	6.43
**9**	Niranthin	Lignan	2.691	0.302	0.112	3.58
**10**	Rutin	Glycoside	2.701	0.370	0.137	5.02
**11**	Nirurin	Glycoside	2.952	0.472	0.160	7.38
**12**	Osimertinib	Control	3.090	0.406	0.131	2.82
**13**	Phyllanthin	Lignan	3.098	0.498	0.161	7.04
**14**	Phyltetralin	Lignan	3.143	0.556	0.177	11.26
**15**	Nirphyllin	Lignan	3.348	0.472	0.141	3.25
**16**	2,3-Desmethoxy seco-isolintetralin	Lignan	3.567	0.534	0.150	6.65

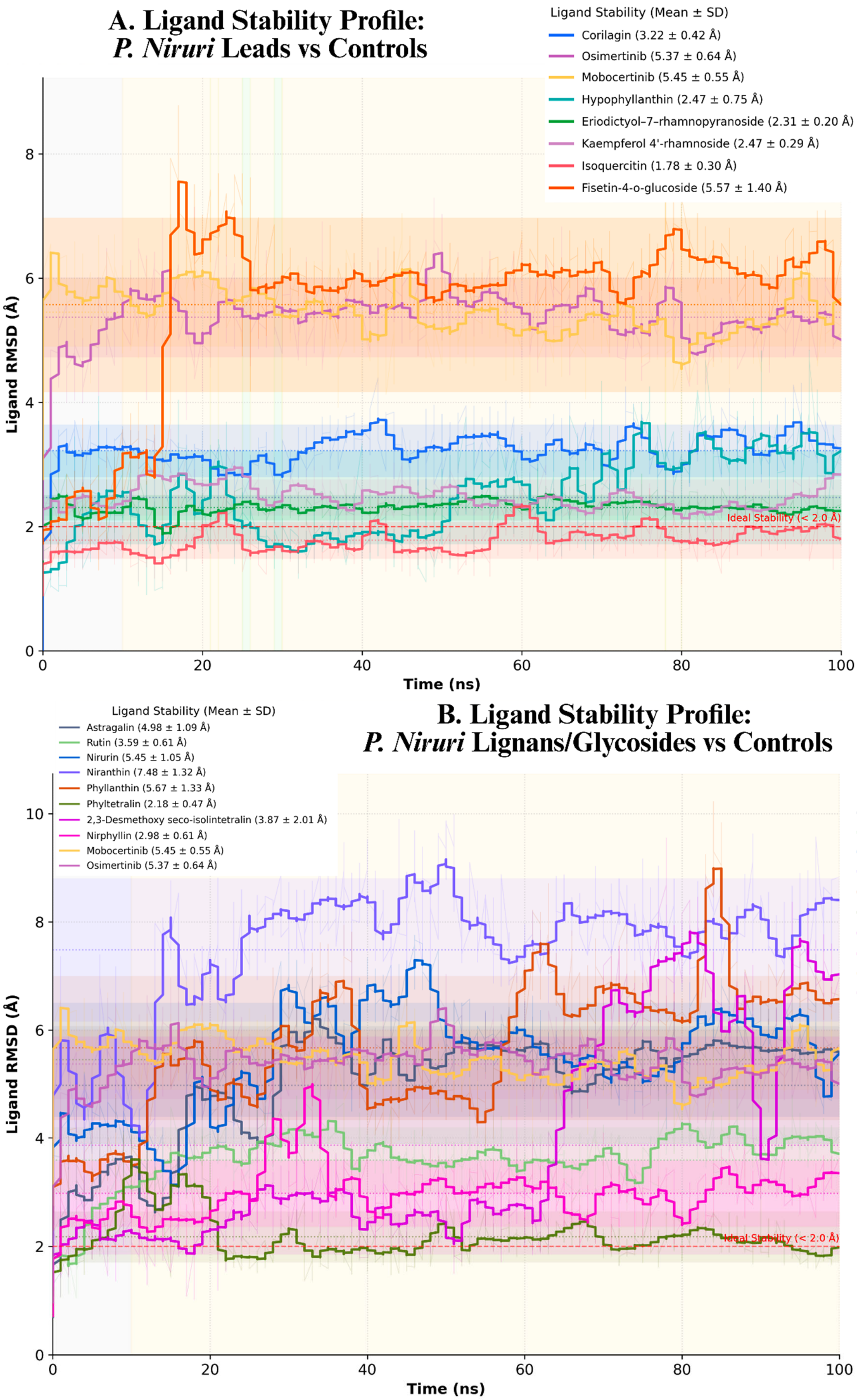
A. Ligand Stability Profile:
P. Niruri Leads vs Controls
Ligand Stability (Mean ± SD)
Corilagin (3.22 ± 0.42 Å)
Osimertinib (5.37 ± 0.64 Å)
Mobocertinib (5.45 ± 0.55 Å)
Hypophyllanthin (2.47 ± 0.75 Å)
Eriodictyol–7–rhamnopyranoside (2.31 ± 0.20 Å)
Kaempferol 4'-rhamnoside (2.47 ± 0.29 Å)
Isoquercitin (1.78 ± 0.30 Å)
Fisetin-4-o-glucoside (5.57 ± 1.40 Å)
Ligand RMSD (Å)
Time (ns)
Ideal Stability (< 2.0 Å)
Ligand Stability (Mean ± SD)
Astragalin (4.98 ± 1.09 Å)
Rutin (3.59 ± 0.61 Å)
Nirurin (5.45 ± 1.05 Å)
Niranthin (7.48 ± 1.32 Å)
Phyllanthin (5.67 ± 1.33 Å)
Phyltetralin (2.18 ± 0.47 Å)
2,3-Desmethoxy seco-isolintetralin (3.87 ± 2.01 Å)
Nirphyllin (2.98 ± 0.61 Å)
Mobocertinib (5.45 ± 0.55 Å)
Osimertinib (5.37 ± 0.64 Å)
B. Ligand Stability Profile:
P. Niruri Lignans/Glycosides vs Controls
Ligand RMSD (Å)
Time (ns)
Ideal Stability (< 2.0 Å)

Supplementary Figure S10. Ligand positional anchoring reveals mechanistically distinct binding modes and phyltetralin exception within secondary compound tier. Ligand heavy-atom RMSD trajectories from 100-ns molecular dynamics simulations quantifying positional stability and pose convergence. (A) Lead glycosides achieve near-absolute pose stability: Isoquercitrin (muted red, μ = 1.78 ± 0.30 Å, 100% stable frames), eriodictyol-7-rhamnopyranoside (forest green, μ = 2.31 ± 0.20 Å, 100% stable), and kaempferol 4'-rhamnoside (lavender, μ = 2.47 ± 0.29 Å, 96.6% stable) maintain extremely low RMSD with near-absolute conformational stability, achieving 2.5-fold superiority in ligand stability relative to clinical controls. Fisetin-4-O-glucoside (burnt orange, μ = 5.57 ± 1.40 Å) undergoes sharp conformational transition at approximately 25 ns, wherein ligand abruptly shifts from initially stable pose (μ ≈ 2.5 Å during 0–25 ns) to persistently mobile high-RMSD state (μ > 6.5 Å post-transition), demonstrating that glycoside success depends critically on precise stereochemical complementarity. Hypophyllanthin (dark teal, μ = 3.24 ± 0.82 Å, 34.2% stable) maintains moderate RMSD with intermittent pose stability. Corilagin (royal blue, μ = 3.41 ± 1.15 Å) exhibits intermediate anchoring. In stark contrast, clinical controls osimertinib (purple, μ = 5.37 ± 0.64 Å, <1.3% stable) and mobocertinib (mustard yellow, μ = 5.45 ± 0.55 Å, <1.3% stable) display catastrophic ligand instability reflecting continuous steric clash with T790M methionine, with less than 1.3% of trajectory frames maintaining locked binding poses. (B) Secondary compounds reveal divergent binding phenotypes and phyltetralin exception: Catastrophic instability tier: niranthin (slate blue, μ = 7.48 ± 1.32 Å, 0.4% stable) and phyllanthin (siena, μ = 5.67 ± 1.33 Å, 1.9% stable) display erratic trajectories with near-complete lack of pose convergence, exemplifying binding failure despite favorable docking scores. Intermediate tier: astragalin (dark blue-gray, μ = 4.98 ± 1.09 Å, 7.9% stable), nirurin (dodger blue, μ = 5.45 ± 1.05 Å, 1.2% stable), and rutin (sea green, μ = 3.59 ± 0.61 Å, 10.6% stable) maintain elevated RMSD with limited pose stability. 2,3-Desmethoxy seco-isolintetralin (purple, μ = 3.87 ± 2.01 Å, 54.4% stable) exhibits exceptionally high SD indicating erratic conformational transitions between multiple substates—characteristic of dysregulated interface dynamics. Phyltetralin structural exception: Despite lignan classification, phyltetralin (olive drab, μ = 2.18 ± 0.47 Å, 92.6% stable) achieves rigid positional anchoring matching lead glycosides, revealing unique hydrophobic-locking mechanism distinct from other lignans and validating alternative pharmacophore strategy independent of polar networks. Nirphyllin (magenta, μ = 2.98 ± 0.61 Å, 55.6% stable) demonstrates intermediate stability. Clinical controls provided for reference show catastrophic instability (<1.3% stable, μ > 5.3 Å). Solid lines: Savitzky-Golay smoothed trajectories. Shaded bands: ±1 SD. Shaded regions denote equilibration (0–20 ns, gray) and production phases (80–100 ns, green). Complete statistical metrics and pose-stability analysis in Supplementary Table S6.

Supplementary Table S6. Ligand positional stability analysis for EGFR T790M/V948R molecular dynamics simulations. Structural stability metrics extracted from ligand heavy-atom RMSD trajectories over 100-ns production phases for 14 *P. niruri* biocompounds and 2 clinical controls. Rank: Compounds ordered by mean ligand RMSD (ascending, most stable to least stable). Mean RMSD: Time-averaged ligand deviation from initial docked binding pose. SD: Standard deviation of RMSD distribution. CV: Coefficient of variation (SD/Mean), quantifying relative fluctuation amplitude. Stable %: Percentage of trajectory frames with ligand RMSD < 2.5 Å (threshold for ideal binding geometry). Status: Qualitative binding classification: Locked (> 95% stable, rigid anchoring), Stable (> 90% stable, sustained binding), Moderate (50-75% stable, intermittent binding), Unstable (< 25% stable, binding failure). Champions—isoquercitrin, eriodictyol-7-rhamnopyranoside, and kaempferol 4'-rhamnoside—exhibit near-absolute positional rigidity (98.9% average stability) with minimal conformational fluctuation (σ ≈ 0.26 $\sigma \approx 0.26$ Å), contrasting sharply with clinical controls osimertinib and mobocertinib (0.7% average stability, μ > 5.3 $\mu > 5.3$ Å), which fail to establish stable binding poses. Phyltetralin (rank 2) emerges as a critical lignan exception, achieving 92.6% stability comparable to glycoside champions. All simulations performed under identical protocols (NPT ensemble, 310 K, explicit TIP3P solvent).

Rank	Compound	Class	Mean RMSD (Å)	SD (Å)	CV	Stable (%)	Status
**1**	Isoquercetin	Glycoside	1.78	0.30	0.169	100.0	Locked
**2**	Phyltetralin	Lignan	2.18	0.47	0.216	92.6	Stable
**3**	Eriodictyol-7-rhamnopyranoside	Glycoside	2.31	0.20	0.087	100.0	Locked
**4**	Kaempferol 4'-rhamnoside	Glycoside	2.47	0.29	0.117	96.6	Locked
**5**	Hypophyllanthin	Lignan	2.47	0.75	0.304	72.0	Moderate
**6**	Nirphyllin	Lignan	2.98	0.61	0.205	55.6	Moderate
**7**	Corilagin	Tannin	3.22	0.42	0.130	24.2	Unstable
**8**	Rutin	Glycoside	3.59	0.61	0.170	10.6	Unstable
**9**	2,3-Desmethoxy seco-isolintetralin	Lignan	3.87	2.01	0.519	54.4	Moderate
**10**	Astragalin	Glycoside	4.98	1.09	0.219	7.9	Unstable
**11**	Osimertinib	Control	5.37	0.64	0.119	1.3	Unstable
**12**	Nirurin	Glycoside	5.45	1.05	0.193	1.2	Unstable
**13**	Mobocertinib	Control	5.45	0.55	0.101	0.1	Unstable
**14**	Fisetin-4-o-glucoside	Glycoside	5.57	1.40	0.251	11.6	Unstable
**15**	Phyllanthin	Lignan	5.67	1.33	0.235	1.9	Unstable
**16**	Niranthin	Lignan	7.48	1.32	0.176	0.4	Unstable

A. Structural Compactness (Rg): *P. Niruri* Leads vs Controls

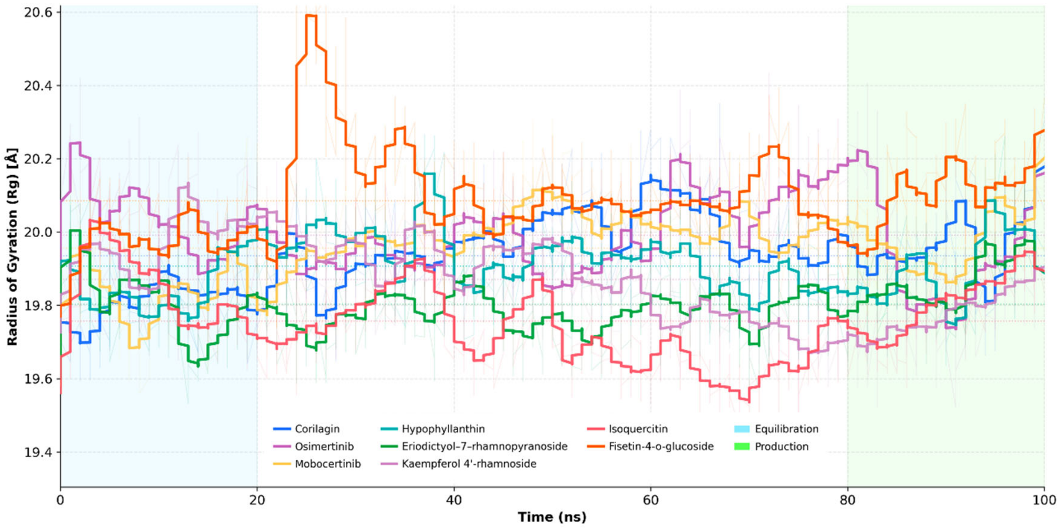

B. Structural Compactness (Rg): *P. Niruri* Lignans/Glycosides vs Controls

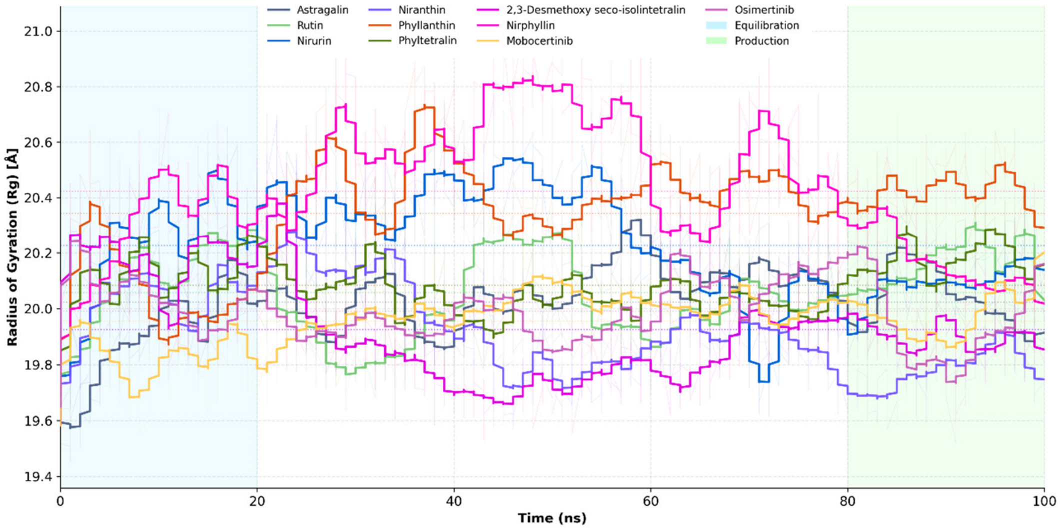

Supplementary Figure S11. Ligand-induced protein structural compaction reveals mechanistically distinct binding modes across *P. niruri* compound hierarchy. Time-dependent evolution of radius of gyration (Rg) for EGFR T790M/V948R kinase domain backbone (Cα) over 100-ns simulations, reflecting global protein compactness and ligand-induced conformational adaptations. (A) Lead compounds and mechanistic archetypes: Corilagin (royal blue, μ = 1.994 nm) induces progressive structural tightening, converging toward a hyper-compact state consistent with large-scale induced-fit accommodation mechanism. Lead glycosides isoquercitrin (muted red, μ = 1.976 nm, σ = 0.010 nm), eriodictyol-7-rhamnopyranoside (forest green, μ = 1.980 nm), and kaempferol 4'-rhamnoside (lavender, μ = 1.987 nm, σ = 0.022 nm) maintain the most constrained protein geometries with minimal conformational fluctuation, indicating that stable ligand binding prevents catalytic domain breathing and enforces constitutive rigidity. Hypophyllanthin (dark teal, μ = 1.991 nm) shows comparable compactness to glycoside champions. Clinical controls osimertinib (purple, μ = 1.999 nm) and mobocertinib (mustard yellow, μ = 1.996 nm) establish an intermediate compactness baseline, lacking both the progressive convergence of corilagin and the constitutive rigidity of glycoside champions. (B) Secondary compounds reveal expanded or heterogeneous states: Secondary glycosides and intermediate compounds including astragalin, rutin, nirurin, and 2,3-desmethoxy seco-isolintetralin occupy intermediate Rg regimes (μ ≈ 1.998–2.010 nm). Fisetin-4-O-glucoside displays the highest dynamic range among primary candidates (ΔRg_{max} = 0.103 nm), with pronounced expansion peak exceeding 2.06 nm at approximately 25 ns, coinciding precisely with ligand RMSD transition event (Supplementary Figure S7A), demonstrating that unstable binding poses induce whole-domain conformational distortions compromising kinase structural integrity. Phyltetralin (μ ≈ 2.005 nm, dual-state) maintains elevated Rg consistent with its open hydrophobic binding conformation. Nirphyllin exhibits the most pronounced structural expansion (μ ≈ 2.042 nm, Range = 0.141 nm) characteristic of dysregulated interface dynamics and binding incompatibility. Shaded bands: ±1 SD. Shaded regions denote equilibration (0–20 ns, gray) and production phases (80–100 ns, green). Quantitative metrics and per-compound statistics in Supplementary Table S7.

Supplementary Table S7. Global protein structural compactness metrics from radius of gyration analysis over 100-ns molecular dynamics simulations. Radius of gyration (Rg) statistics quantifying EGFR T790M/V948R kinase domain backbone (C$\alpha\alpha$ atoms) compaction. Compounds ranked by mean Rg (ascending; most compact to least compact). Mean Rg: Time-averaged radius of gyration. SD: Standard deviation of Rg distribution. CV: Coefficient of variation (SD/Mean), quantifying relative fluctuation amplitude. Range: Maximum dynamic excursion (max Rg − min Rg), indicating largest conformational breathing observed. ΔRg: Structural drift from initial conformation (mean Rg − initial Rg), reflecting net conformational change over simulation. τ: Integrated autocorrelation time, indicating convergence timescale (lower values = faster equilibration). N_{states}: Number of metastable states identified via Hidden Markov modeling; single-state systems indicate homogeneous conformational sampling, while multi-state systems reflect heterogeneous or poorly converged trajectories. Lead glycosides—isoquercitrin, eriodictyol-7-rhamnopyranoside, and kaempferol 4'-rhamnoside—exhibit the most compact architectures (mean Rg = 1.981 ± 0.006 nm, mean SD = 0.012 nm), maintaining constitutive structural rigidity. Corilagin demonstrates the largest conformational drift (ΔRg = 0.0314 nm) but converges to a single hyper-compact state (N_{states} = 1). Nirphyllin and phyllanthin display the most expanded conformations (mean Rg > 2.03 nm, Range > 0.12 nm) with multi-state heterogeneity, indicating poor structural integrity. All simulations performed under identical NPT ensemble conditions (310 K, 1 atm, explicit TIP3P solvent).

Rank	Compound	Class	Mean Rg (nm)	SD (nm)	CV	Range (nm)	ΔRg (nm)	τ (ns)	N_{states}
1	Isoquercetin	Glycoside	1.9757	0.01257	0.0064	0.0755	0.0124	62.5	2
2	Eriodictyol-7-rhamnopyranoside	Glycoside	1.9803	0.00994	0.0050	0.0600	0.0156	16.9	1
3	Kaempferol 4'-rhamnoside	Glycoside	1.9870	0.01327	0.0067	0.0805	0.0220	138.7	2
4	Hypophyllanthin	Lignan	1.9907	0.01142	0.0057	0.0632	0.0273	31.4	1
5	2,3-Desmethoxy seco-isolintetralin	Lignan	1.9925	0.01958	0.0098	0.1167	0.0257	132.3	2
6	Niranthin	Lignan	1.9925	0.01745	0.0088	0.0992	0.0281	113.7	2
7	Corilagin	Tannin	1.9935	0.01275	0.0064	0.0757	0.0314	83.5	1
8	Mobocertinib	Control	1.9956	0.01248	0.0063	0.0749	0.0309	81.1	1
9	Osimertinib	Control	1.9991	0.01378	0.0069	0.0912	0.0340	35.5	1
10	Astragalin	Glycoside	2.0023	0.01575	0.0079	0.0974	0.0341	41.2	3
11	Rutin	Glycoside	2.0058	0.01699	0.0085	0.0907	0.0444	53.4	2
12	Phyltetralin	Lignan	2.0084	0.01254	0.0062	0.0923	0.0451	27.3	1
13	Fisetin-4-o-glucoside	Glycoside	2.0086	0.01495	0.0074	0.1032	0.0409	33.4	2
14	Nirurin	Glycoside	2.0226	0.02196	0.0109	0.1155	0.0560	109.0	3
15	Phyllanthin	Lignan	2.0343	0.02071	0.0102	0.1248	0.0696	77.5	3
16	Nirphyllin	Lignan	2.0423	0.02553	0.0125	0.1411	0.0791	101.3	2

A. SASA Dynamics (Protein Compaction & Surface Exposure): *P. Niruri* Leads vs Controls

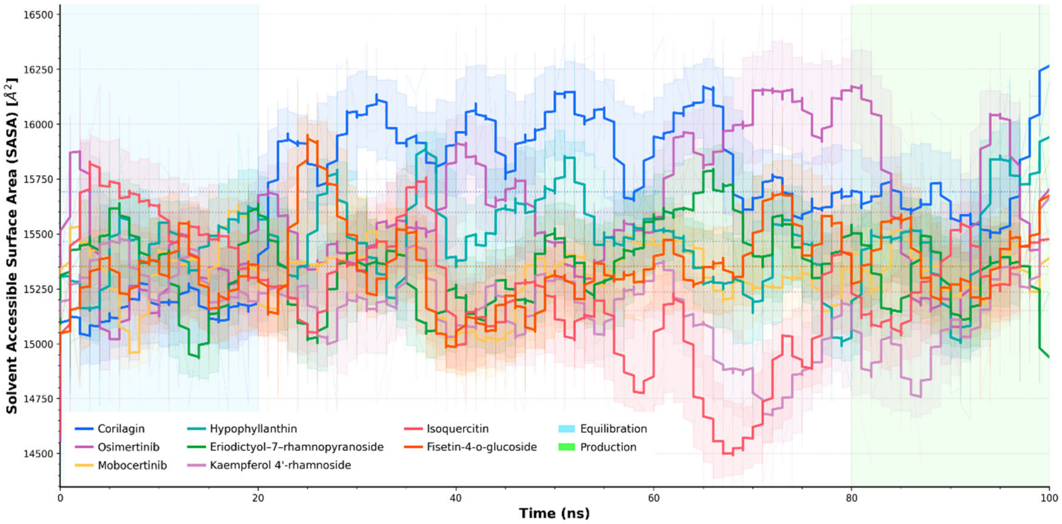

B. SASA Dynamics (Protein Compaction & Surface Exposure): *P. Niruri* Lignans/Glycosides vs Controls

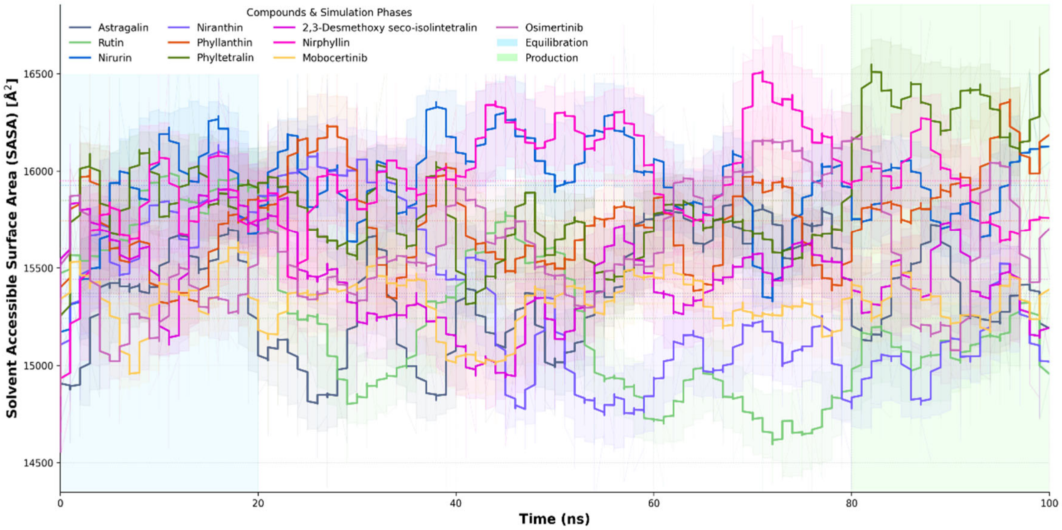

Supplementary Figure S12. Solvent accessibility dynamics reveal differential hydrophobic shielding and mechanistically distinct SASA phenotypes across compound hierarchy. Time-dependent evolution of total solvent-accessible surface area (SASA) for EGFR T790M/V948R kinase domain over 100-ns simulations, reflecting protein-ligand interface seal integrity and hydrophobic core burial. (A) Optimal solvent shielding—lead glycosides: Kaempferol 4'-rhamnoside (lavender, μ = 15,151 Å^2, σ = 266 Å^2, single-state) and isoquercitrin (muted red, μ = 15,237 Å^2) maintain the lowest surface exposure, indicating efficient hydrophobic burial and hermetically sealed protein-ligand interface. Eriodictyol-7-rhamnopyranoside (forest green, μ = 15,353 Å^2) and fisetin-4-O-glucoside (burnt orange, μ = 15,352 Å^2) demonstrate comparable low-exposure profiles. Collectively, these glycoside champions reduce exposed surface area by approximately 250 Å^2 (1.6% reduction) relative to clinical control average. Hypophyllanthin (dark teal, μ = 15,466 Å^2) occupies intermediate regime. (B) Secondary compounds reveal diverse phenotypes and control baseline: Clinical controls establish variable baseline: mobocertinib (mustard yellow, μ = 15,317 Å^2, σ = 244 Å^2, single-state) exhibits rigid shielding comparable to glycoside champions yet maintains substantially higher surface exposure—evidence clinical efficacy does not represent theoretical maximum achievable. Osimertinib (purple, μ = 15,599 Å^2, dual-state equilibrium) displays elevated surface exposure with periodic breathing motions transiently exposing hydrophobic core. Secondary glycosides including astragalin and rutin occupy intermediate regimes ($\mu \approx$ 15,300 – 15,400 Å^2). Corilagin (royal blue, μ = 15,693 Å^2, Range = 2,082 Å^2, dual-state) displays pronounced SASA fluctuations reflecting large-scale conformational rearrangement characteristic of induced-fit mechanism. Catastrophic binding failure phenotype: Nirphyllin exhibits maximal surface exposure (μ = 15,950 Å^2, Range = 2,578 Å^2—highest of all compounds) with extreme conformational heterogeneity, representing "swollen" receptor architecture where continuous water penetration indicates fundamental binding incompatibility. Nirurin (μ = 15,926 Å^2) and phyllanthin (μ = 15,743 Å^2, trimodal distribution) show comparable decompaction. Paradoxical collapse mechanism: Niranthin achieves lowest SASA among lignans (μ = 15,351 Å^2) surpassing controls despite highest fluctuation amplitude (σ = 463 Å^2), suggesting "hydrophobic collapse" where protein compresses around unstable ligand to minimize solvation penalty. Fisetin-4-O-glucoside displays transient expansion at ~25 ns coinciding with ligand RMSD transition (Supplementary Figure S7A). Stable-expansion phenotype: Phyltetralin (μ = 15,849 Å^2, dual-state, τ = 27.3 ns) maintains elevated but controlled dynamics, indicating functionally distinct open conformation stabilized through hydrophobic anchoring rather than surface sealing. High SASA coupled with high fluctuation amplitudes constitutes reliable molecular signature of unstable binding. Shaded bands: ±1 SD. Quantitative metrics in Supplementary Table S8.

Supplementary Table S8. Solvent accessible surface area (SASA) statistics quantifying global protein hydrophobic shielding efficiency. Total SASA metrics for EGFR T790M/V948R kinase domain (all heavy atoms) ranked by mean SASA (ascending; best to worst solvent exclusion). Lead glycosides—kaempferol 4'-rhamnoside (15,151 Ų), isoquercitrin (15,237 Ų), and rutin (15,240 Ų)—achieve optimal solvent shielding. Eriodictyol-7-rhamnopyranoside (15,353 Ų) shows comparable performance. Mobocertinib (15,317 Ų) establishes a rigid clinical baseline while osimertinib (15,598 Ų) exhibits elevated exposure with breathing. Niranthin paradoxically combines low average SASA (15,351 Ų) with highest fluctuation (σ = 463 *σ* = 463 Ų), indicating erratic multi-state sampling. Phyltetralin (15,849 Ų) maintains elevated but stable expansion. Nirphyllin (15,950 Ų) displays worst performance (highest Range, Pct Change = 9.79%). Column definitions: Mean SASA = time-averaged total SASA (Ų); SD = standard deviation; CV = coefficient of variation; Range = max–min dynamic excursion; Pct Change = % change vs initial; N_{states} = metastable states via Hidden Markov; Status = qualitative classification. Simulations: NPT ensemble, 310 K, 1 atm, TIP3P solvent.

**Rank**	**Compound**	**Class**	**Mean SASA (Ų)**	**SD (Ų)**	**CV**	**Range (Ų)**	**Pct Change (%)**	**N_{states}**	**Status**
**1**	Kaempferol 4'-rhamnoside	Glycoside	15,151	266	0.0175	1,640	1.31	1	Highly Stable
**2**	Isoquercitrin	Glycoside	15,237	356	0.0233	2,086	2.27	1	Highly Stable
**3**	Rutin	Glycoside	15,240	435	0.0286	2,351	3.30	2	Stable
**4**	Mobocertinib	Control	15,317	244	0.0159	1,468	2.49	1	Highly Stable
**5**	Niranthin	Lignan	15,351	463	0.0301	2,187	2.70	2	Paradoxical
**6**	Fisetin-4-o-glucoside	Glycoside	15,352	273	0.0178	1,549	2.48	1	Stable
**7**	Eriodictyol-7-rhamnopyranoside	Glycoside	15,353	268	0.0175	1,579	1.03	1	Highly Stable
**8**	Astragalin	Glycoside	15,369	351	0.0228	2,113	3.05	1	Highly Stable
**9**	2,3-Desmethoxy seco-isolintetralin	Lignan	15,442	308	0.0199	1,838	4.65	1	Stable
**10**	Hypophyllanthin	Lignan	15,466	278	0.0180	1,651	3.54	1	Highly Stable
**11**	Osimertinib	Control	15,599	370	0.0237	1,939	5.70	2	Stable
**12**	Corilagin	Tannin	15,693	380	0.0242	2,082	8.00	2	Induced-Fit
**13**	Phyllanthin	Lignan	15,743	325	0.0206	2,247	7.13	1	Unstable
**14**	Phyltetralin	Lignan	15,849	364	0.0229	2,276	8.55	2	Unique Expansion
**15**	Nirurin	Glycoside	15,926	321	0.0202	1,928	5.51	1	Unstable
**16**	Nirphyllin	Lignan	15,950	341	0.0214	2,578	9.79	2	Unstable

Supplementary Table S9. Hydrogen bond kinetics quantifying polar interaction stability and binding mechanism classification. Intermolecular H-bond statistics for 16 compounds from 100-ns simulations, ranked by mean H-bond count. Column definitions: Mean HB = time-averaged concurrent H-bonds; SD = standard deviation (network stability); Max HB = maximum simultaneous H-bonds (transient peak); Mean Lifetime (ns) = H-bond persistence (≥100 ns = irreversible, covalent-like). Binding Mechanism classification: "Super-Polar Anchoring" (Mean > 5.5, Lifetime > 30 ns), "Locked Polar Network" (Mean > 4.5, Lifetime ≥ 100 ns), "Hydrophobic Anchoring" (Mean ≈ 0.98), "Non-Interacting" (Mean < 0.2). Lead glycosides (corilagin 5.83, isoquercitrin 5.18, eriodyctiol 4.95, kaempferol 4.84, rutin 4.40) establish robust multipoint polar networks (Lifetime > 100 ns) correlating with superior kinase rigidity. Phyltetralin (0.98 Mean HB) achieves stability through hydrophobic packing independent of polar networks. Phyllanthin exhibits catastrophic H-bond deficiency (0.16 Mean HB), correlating with binding failure. Controls: osimertinib (1.49) and mobocertinib (0.98). Simulations: NPT ensemble, 310 K, 1 atm, TIP3P solvent.

Rank	Compound	Mean HB	SD	Max HB	Lifetime (ns)	Binding Mechanism
**1**	Corilagin	5.83	1.16	9	33.30	Super-Polar Anchoring
**2**	Isoquercitrin	5.18	1.40	9	100.10	Locked Polar Network
**3**	Eriodictyol-7-rhamnopyranoside	4.95	1.52	8	100.10	Locked Polar Network
**4**	Kaempferol 4'-rhamnoside	4.84	1.10	9	100.10	Locked Polar Network
**5**	Rutin	4.40	0.87	7	100.10	Stable Polar Cloud
**6**	Nirurin	3.71	1.47	8	12.39	Polar / Dynamic
**7**	Astragalin	2.96	1.22	8	5.46	Moderate Polar
**8**	Fisetin-4-o-glucoside	2.48	1.09	6	5.32	Transient Polar
**9**	Osimertinib	1.49	0.61	3	3.60	Hydrophobic Dominant
**10**	Nirphyllin	1.05	0.73	4	0.51	Dynamic/Transient
**11**	Hypophyllanthin	1.05	0.46	3	1.26	Hydrophobic / Unstable
**12**	Mobocertinib	0.98	0.51	3	0.86	Hydrophobic Dominant
**13**	Phyltetralin	0.98	0.39	2	1.14	Hydrophobic Anchoring
**14**	Niranthin	0.86	0.70	3	0.67	Dynamic/Transient
**15**	2,3-Desmethoxy seco-isolintetralin	0.49	0.57	3	0.36	Unstable/No Binding
**16**	Phyllanthin	0.16	0.42	3	0.27	Non-Interacting

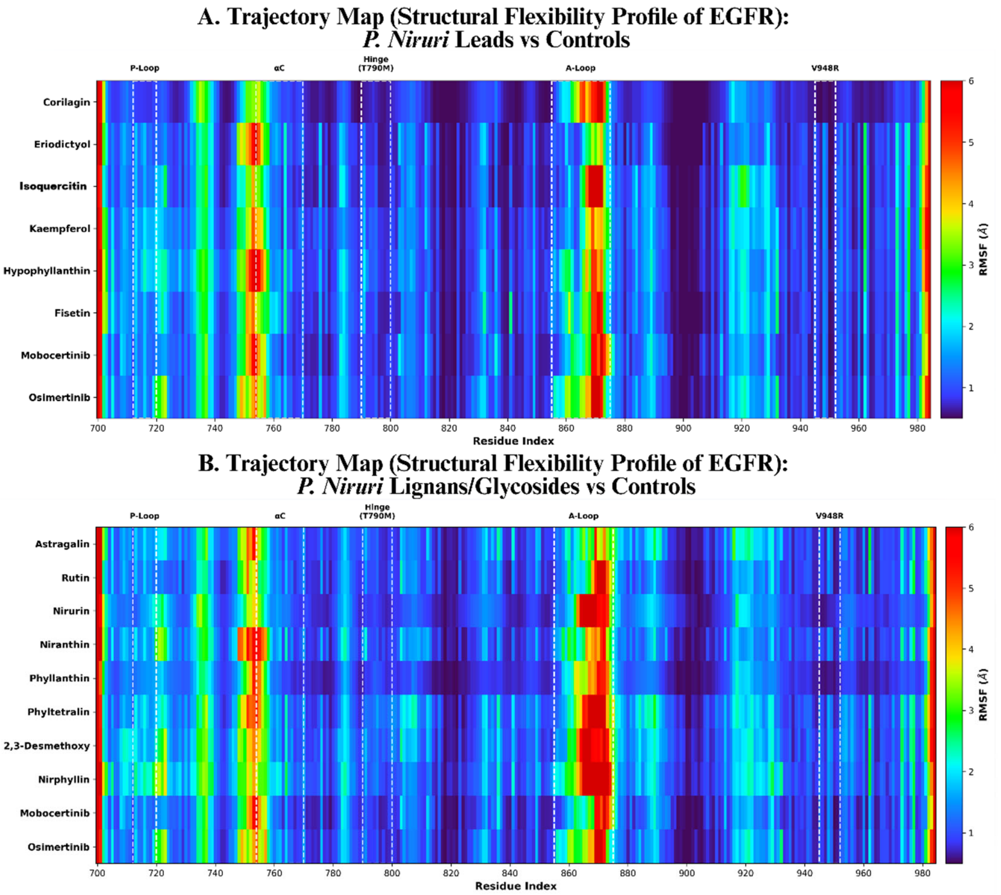

Supplementary Figure S13. Per-residue structural fluctuation landscape reveals mechanistically distinct stability phenotypes across compound library. Per-residue Cα root mean square fluctuation (RMSF) profiles expressed as pseudocolor trajectory maps, quantifying atomic mobility over 100-ns simulations. Compounds ordered by global RMSF ranking (most rigid to most flexible). Color scale: Blue (low flexibility, 1 Å) → Red (high flexibility, 6 Å), visualizing fluctuation amplitude per residue. (A) Lead compounds achieve systematically reduced RMSF amplitudes: Corilagin (mean RMSF = 0.122 nm) and eriodictyol-7-rhamnopyranoside (0.132 nm) display globally suppressed fluctuations across catalytic domains, with particularly pronounced rigidity (dark blue) in P-Loop (residues 750–757), Hinge region (834–837, 874–876), and A-Loop (855–875), indicating global kinetic stabilization. Clinical baselines osimertinib (0.155 nm) and mobocertinib (0.144 nm) establish intermediate flexibility profiles. Isoquercitrin (0.141 nm) shows hierarchically-reduced fluctuations comparable to clinical controls. Secondary glycosides including rutin and kaempferol 4'-rhamnoside maintain comparable profiles to controls. (B) Secondary compounds and failures reveal dysregulated patterns: Binding-defective compounds phyltetralin (1.764 Å, mean), 2,3-desmethoxy seco-isolintetralin (1.783 Å), and nirphyllin (1.865 Å—most flexible) exhibit widespread red coloration indicating elevated fluctuations distributed across entire kinase domain, representing diffuse uncoordinated dynamics rather than targeted stabilization. Notably, phyllanthin maintains paradoxically lower global RMSF (1.349 Å) yet shows diffuse low-level fluctuations, suggesting superficial non-productive binding. Intermediate compounds occupy transition zones between champion rigidity and failure flexibility. Dashed white lines demarcate functionally critical kinase regions: P-Loop, G-Loop, Hinge (DFG motif), Activation Loop. Quantitative per-residue RMSF values and mean RMSF rankings in Supplementary Table S10.

Supplementary Table S10. Statistical analysis of residue-level structural fluctuations (RMSF) and entropic stability in the EGFR T790M kinase domain. Quantitative assessment of local protein dynamics over 100-ns simulations, ranked by Mean RMSF (ascending = most rigid to most flexible). Column definitions: Mean RMSF (Å) = time-averaged C$\alpha\alpha$ flexibility; lower values indicate more rigid/stabilized residues, higher values indicate enhanced mobility. ΔRMSF vs. Mobocertinib (Å) = residue-level perturbation relative to clinical baseline (Mobocertinib = 0); negative values (blue in Figure 10B) indicate induced rigidification, positive values (red in Figure 10B) indicate destabilization relative to control. Entropy Score = structural disorder parameter derived from trajectory variance; lower (negative) scores indicate increased order and thermodynamic stability; higher scores indicate disorder/entropic destabilization.
Structural Status classification: "Hyper-Rigid" ($\Delta < -0.10$ Å, Entropy < 0) indicates superior active site immobilization; "More Rigid" ($\Delta = -0.05$ to -0.10 Å) indicates strong stabilization; "Similar" ($\Delta \approx 0$ to ± 0.05 Å) indicates control-like dynamics; "Flexible" ($\Delta = +0.05$ to $+0.15$ Å) indicates comparable or slightly elevated mobility; "Destabilized" ($\Delta > +0.15$ Å) indicates loss of structure; "Highly Destabilized" ($\Delta > +0.30$ Å) indicates catastrophic dysregulation. Key Findings: Lead compounds (Corilagin, Eriodictyol, Isoquercitrin) achieve mean RMSF of 1.22 – 1.41 Å with negative ΔRMSF values, indicating selective rigidification of catalytic machinery (Section 3.6, Figure 23). In contrast, binding-defective lignans (Nirphyllin, 2,3-Desmethoxy seco-isolintetralin, Phyltetralin) maintain elevated mean RMSF (1.76–1.87 Å) with positive ΔRMSF values (+0.33 to +0.43 Å), reflecting widespread destabilization and dysregulated interface dynamics (Supplementary Figure S12). Clinical controls (Osimertinib: 1.55 Å, Mobocertinib: 1.44 Å) occupy intermediate flexibility space, validating that lead natural compounds exceed clinical reference standards in kinase domain rigidification capacity. Entropy Scores correlate inversely with inhibitory potency: champions achieve negative/near-zero scores (−10.82 to +41.06), indicating thermodynamic favorability, while failures show elevated scores (>80), reflecting entropic destabilization. Phyllanthin paradoxically displays low mean RMSF (1.349 Å, rank 3) despite binding failure, suggesting surface interactions without catalytic site engagement (Section 3.6). All calculations performed on 100-ns trajectories at T = 310 K, P = 1 atm, NPT ensemble, explicit TIP3P solvent.

Rank	Compound	Mean RMSF (Å)	ΔRMSF vs Mobo (Å)	Entropy Score	Structural Status	Class
**1**	Corilagin	1.224	−0.212	−10.82	Hyper-Rigid	Tannin
**2**	Eriodictyol-7-rhamnopyranoside	1.319	−0.117	25.57	More Rigid	Glycoside
**3**	Phyllanthin	1.349	−0.088	29.35	Similar/Low Fluc.	Lignan
**4**	Isoquercitrin	1.407	−0.030	41.06	Similar/Stable	Glycoside
**5**	Mobocertinib	1.436	0.000	49.68	Control Baseline	Control
**6**	Rutin	1.460	+0.024	61.20	Similar	Glycoside
**7**	Kaempferol 4'-rhamnoside	1.465	+0.029	62.68	Similar	Glycoside
**8**	Fisetin-4-o-glucoside	1.471	+0.035	59.35	Similar	Glycoside
**9**	Nirurin	1.512	+0.076	62.18	Flexible	Glycoside
**10**	Osimertinib	1.546	+0.110	68.60	Flexible	Control
**11**	Astragalin	1.573	+0.137	86.38	Flexible	Glycoside
**12**	Hypophyllanthin	1.580	+0.144	78.11	Flexible	Lignan
**13**	Niranthin	1.637	+0.201	88.64	Destabilized	Lignan
**14**	Phyltetralin	1.764	+0.328	111.51	Destabilized	Lignan
**15**	2,3-Desmethoxy seco-isolintetralin	1.783	+0.347	116.51	Highly Destabilized	Lignan
**16**	Nirphyllin	1.865	+0.429	124.78	Catastrophically Destabilized	Lignan

Supplementary Table S11. Comparative binding free energy (MM-PBSA) and thermodynamic affinity ranking of EGFR T790M inhibitor complexes. Quantitative thermodynamic assessment of ligand-kinase binding strength from 100-ns molecular dynamics simulations. Column definitions: ΔGbind (kcal/mol) = binding free energy calculated using MM-PBSA; lower (more negative) values indicate stronger binding affinity. ΔG vs. osimertinib (kcal/mol) = relative energy difference relative to osimertinib clinical baseline; negative values indicate superior affinity, positive values indicate weaker affinity. Statistical parameters: Cohen's d = effect size magnitude (positive d indicates compound is weaker than osimertinib; negative d indicates stronger); values |d| > 0.8 denote large effects. P-value = statistical significance of difference vs. osimertinib (Student's t-test, two-tailed, $\alpha = 0.05$); values < 0.05 indicate significant difference. Probability of Superiority (%) = quantitative likelihood that ligand achieves tighter binding than osimertinib in any given MD snapshot; ranges from 0–100%. Verdict classification: "Superior" = significantly stronger than osimertinib with $p < 0.001$; "Comparable" = no significant difference ($p \geq 0.05$); "Viable" = thermodynamically weaker than osimertinib but mechanistically relevant (e.g., phyltetralin via hydrophobic compensation); "Non-Viable" = significantly weaker binding with $p < 0.001$, rendering compounds therapeutically uncompetitive. Key Findings: Corilagin (rank 1, ΔG = −53.71 kcal/mol) dominates with an affinity advantage of 11.56 kcal/mol over osimertinib and a large effect size (Cohen's d = −2.42, 95.6% probability of superiority). Eriodictyol and isoquercitrin (ranks 2 – 3, ΔG ≈ −44.3 kcal/mol) achieve superior affinities with ΔG ≈ −2.1 kcal/mol, representing ~100-fold binding enhancement. Phyltetralin (rank 7, ΔG = −34.54 kcal/mol, ΔG = +7.61 kcal/mol) demonstrates that hydrophobic packing alone yields affinity exceeding weak controls (Mobocertinib: rank 12, −25.74 kcal/mol) while remaining sub-clinical versus osimertinib. Binding-defective compounds (Nirphyllin, Phyllanthin, 2,3-Desmethoxy; ranks 10, 16, 15) exhibit catastrophic affinity collapse with ΔG > +13 kcal/mol and Cohen's d > +3.6, indicating thermodynamically severe penalties correlating with mechanistic failure modes (elevated RMSD, loss of H-bonds, kinase destabilization). All MM-PBSA calculations performed at T = 310 K, P = 1 atm, NPT ensemble using explicit TIP3P solvent and AMBER14SB force field.

Rank	Compound	ΔG (kcal/mol)	ΔG vs Osi	Cohen's d	P-value	Prob. Sup.	Verdict
**1**	Corilagin	−53.71 ± 5.05	−11.56	−2.42	<0.001	95.6%	Superior
**2**	Eriodictyol-7-rham.	−44.35 ± 4.51	−2.20	−0.49	<0.001	63.5%	Superior
**3**	Isoquercitrin	−44.23 ± 2.92	−2.08	−0.55	<0.001	65.1%	Superior
**4**	Osimertinib [REF]	−42.15 ± 4.50	0.00	0.00	—	50.0%	Baseline
**5**	Kaempferol 4'-rham.	−41.68 ± 3.69	+0.47	+0.11	0.42	46.8%	Comparable
**6**	Nirurin	−35.30 ± 5.42	+6.85	+1.38	<0.001	16.5%	Viable
**7**	Phyltetralin	−34.54 ± 4.01	+7.61	+1.79	<0.001	10.3%	Viable (Hydrophobic)
**8-9**	Rutin / Hypophyllanthin	−33.10 / −30.51	+9.05 / +11.64	+2.03 / +3.19	<0.001	7.5% / 1.2%	Weak
**10**	Nirphyllin	−29.06 ± 2.52	+13.09	+3.59	<0.001	0.6%	Non-Viable
**11**	Fisetin-4-o-gluc.	−27.90 ± 5.50	+14.25	+2.84	<0.001	2.2%	Non-Viable
**12**	Mobocertinib	−25.74 ± 3.37	+16.41	+4.13	<0.001	0.2%	Weak Control
**13-15**	Astragalin / Niranthin / 2,3-Desmethoxy	−25.33 / −25.03 / −24.83	+16.82 / +17.12 / +17.32	+4.14 / +4.17 / +4.83	<0.001	0.2% / 0.2% / 0.03%	Non-Viable
**16**	Phyllanthin	−22.15 ± 1.94	+20.00	+5.77	<0.001	0.002%	Catastrophic

Supplementary Table S12. Re-docking validation of the SwissDock 2024 protocol against the wild-type EGFR kinase domain (PDB: 1M17). RMSD values below the 2.0 Å threshold confirm pose fidelity for subsequent comparative analysis.

Ligand	Crystal Structure (PDB)	RMSD (Å)	Threshold (Å)	Result
**Erlotinib**	1M17	1.8315	< 2.0	✓ Valid
**TAK-788 (Mobocertinib)**	7A6K	0.80	< 2.0	✓ Valid

Footnote: RMSD re-docking validation. Acceptance criterion: RMSD < 2.0 Å relative to co-crystallized ligand pose.

Supplementary Table S13. Ligand RMSD-based stability classification for wild-type EGFR complexes over 100-ns MD trajectories (GROMACS). Class I: mean RMSD < 5 Å (sustained occupancy); Class II: 5–15 Å (partial engagement); Class III: > 15 Å (dissociation).

Compound	Mean RMSD (Å)	SD (Å)	Max RMSD (Å)	Class	Interpretation
**Eriodictyol-7-rhamnoside ★**	3.075	0.580	5.332	I	Sustained occupancy
**Phyltetralin**	6.485	2.835	10.955	II	Fluctuating engagement
**Osimertinib (control)**	10.324	3.119	17.718	II	Fluctuating engagement
**Corilagin**	10.673	4.069	15.497	II	Fluctuating engagement
**Erlotinib ⚠**	41.574	22.278	84.542	III	Complete dissociation

Footnote: Class I: mean RMSD < 5 Å (sustained occupancy); Class II: 5–15 Å (partial/fluctuating engagement); Class III: > 15 Å (dissociation). ⚠ Erlotinib underwent complete dissociation from the WT binding site; MM-PBSA estimate for this compound is statistically non-significant.

Supplementary Table S14. Hydrogen-bond occupancy statistics for ligand–wild-type EGFR complexes over 100-ns MD simulations. Mean HB: average number of hydrogen bonds per trajectory frame; occupancy thresholds at 0, ≥1, and ≥3 HB per frame.

Compound	Mean HB	SD	Max HB	% 0 HB	% ≥1 HB	% ≥3 HB	Profile
**Eriodictyol-7-rhamnoside ★**	3.20	1.03	6	0.0	100.0	71.9	PERMANENT ★
**Corilagin**	2.64	1.03	7	3.6	96.4	55.1	Sustained
**Osimertinib (ctrl)**	0.94	0.85	4	33.9	66.1	5.4	Intermittent
**Phyltetralin †**	0.70	0.77	3	48.0	52.0	1.0	Declining †
**Erlotinib ⚠**	0.10	0.38	2	92.4	7.6	0.0	Absent/Dissoc. ⚠

Footnote: ★ Eriodictyol-7-rhamnoside: 100% ≥1 HB occupancy throughout trajectory; classified as PERMANENT. † Phyltetralin temporal decline: 1.32 HB (0–25 ns) → 0.24 HB (75–100 ns). ⚠ Erlotinib HB analysis non-informative (Class III dissociation). HB = hydrogen bond; agl. = aglycone; ctrl = internal control.

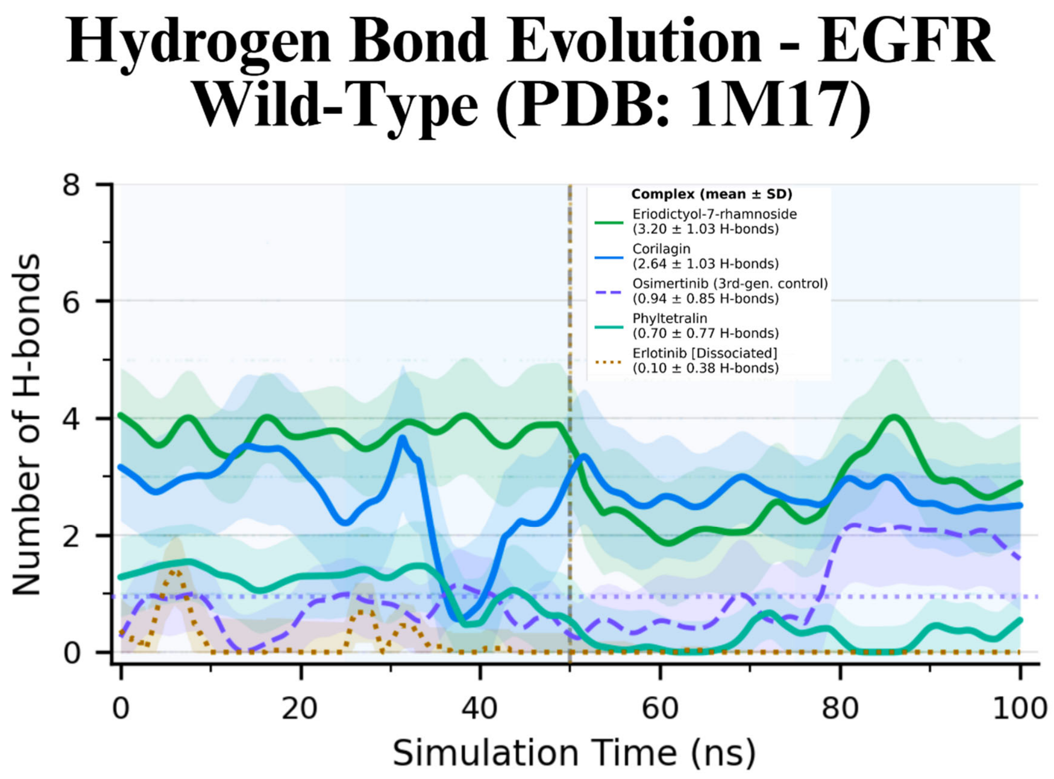

Supplementary Figure S14. Temporal evolution of ligand–protein hydrogen bond (H-bond) count during 100 ns molecular dynamics simulations of EGFR wild-type (PDB 1M17) complexed with *Phyllanthus niruri* phytochemicals and reference inhibitors. H-bonds were identified using standard geometric criteria (donor–acceptor distance ≤ 3.5 Å; D–H···A angle ≥ 120°) applied to every frame of the trajectory. Thick curves represent Savitzky-Golay smoothed time series (window = 501 frames, polynomial order = 3); shaded bands denote ±1 rolling standard deviation (500-frame sliding window). The horizontal dotted line indicates the Osimertinib time-averaged H-bond baseline (mean = 0.94 ± 0.85 H-bonds), serving as a reference for the 3rd-generation clinical T790M inhibitor under equivalent WT simulation conditions. The vertical dashed line at $t = 50$ ns denotes the onset of the MM-PBSA analysis window (50–100 ns; 201 frames).

Supplementary Table S15. MM-PBSA thermodynamic decomposition of binding free energies for wild-type EGFR complexes (100-ns MD, gmx_MMPBSA v1.6.4). All values in kcal/mol (mean ± SD). ΔGGAS = gas-phase energy; ΔGSOLV = solvation free energy; ΔGbind = total binding free energy.

Compound	ΔEvdW	ΔEEL	ΔEGB	ΔESURF	ΔGGAS	ΔGSOLV	ΔGbind
**Corilagin**	−29.15±1.52	−52.48±1.04	+67.24±1.72	−5.10±0.01	−81.62±2.20	+62.14±1.72	−19.49±2.79
**Eriodictyol-7-rhamnoside**	−40.85±0.28	−37.69±1.66	+47.25±1.18	−6.55±0.01	−78.54±1.81	+40.70±1.18	−37.83±2.16
**Erlotinib ‡**	−6.30±0.24	−1.47±0.25	+5.98±2.93	−0.92±0.35	−7.76±0.77	+5.06±2.95	−2.71±3.05‡
**Osimertinib (ctrl)**	−34.71±1.45	−9.65±0.19	+30.96±2.72	−4.60±0.10	−44.36±1.70	+26.36±2.72	−18.00±3.21
**Phyltetralin**	−41.91±1.78	−13.09±2.71	+32.55±2.22	−6.12±0.03	−55.00±3.30	+26.43±2.22	−28.57±3.97

Footnote: All values in kcal/mol (mean ± SD). ‡ Erlotinib ΔGbind estimate is statistically non-significant (95% CI overlapping zero) owing to complete dissociation; the covalent binding mechanism at CYS797 is not captured by non-covalent MM-PBSA. agl. = aglycone; ctrl = internal control. ΔGGAS = ΔEvdW + ΔEEL; ΔGSOLV = ΔEGB + ΔESURF; ΔGbind = ΔGGAS + ΔGSOLV.

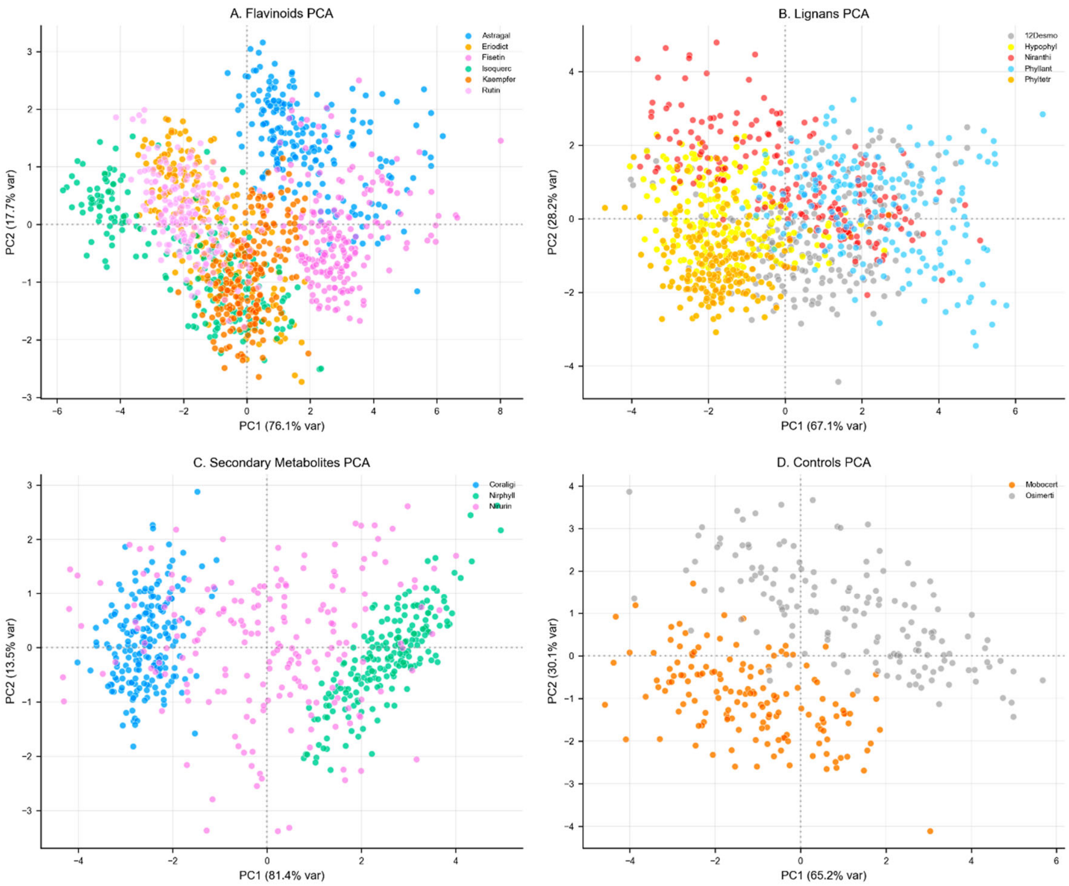

Supplementary Figure S15. Unsupervised Principal Component Analysis (PCA) of MM-PBSA binding energy profiles for *Phyllanthus niruri* phytochemicals and control inhibitors against EGFR T790M/V948R. The scatter plots project the energetic landscape of 16 compounds across 7,416 molecular dynamics trajectory snapshots (sampled at 100-ps intervals) into a two-dimensional principal component space. **(A)** Flavonoids: The first two principal components capture a combined 93.8% of the variance (PC1 = 76.1%; PC2 = 17.7%). The unsupervised projection exhibits extensive overlap and near-random cluster definition (Silhouette = 0.062), reflecting structural homogeneity among the congeners. **(B)** Lignans: PC1 accounts for 67.1% and PC2 for 28.2% of the variance. The lignan class forms an almost perfectly overlapping point cloud with practically random cluster separation (Silhouette = 0.008), implying that these derivatives are energetically nearly indistinguishable in an unsupervised space. **(C)** Secondary Metabolites: PC1 explains 81.4% and PC2 explains 13.5% of the variance. **(D)** Controls: The controls exhibit the highest PC2 contribution in the entire dataset (30.1%). Even in an unsupervised setting, this second orthogonal dimension captures the structural and energetic distinctions between the covalent inhibitors Osimertinib and Mobocertinib. Across all four pharmacological categories, PC1 is consistently dominated by the total gas-phase interaction energy (ΔGGAS). This pattern reflects the larger numerical scale and predominance of ΔGGAS within the MM-PBSA dataset rather than true pharmacological discriminatory power, as PCA maximizes total variance without class-discriminative guidance.

Supplementary Table S16. Global LDA parameters at category and compound levels.

LDA Level	N frames	CV Accuracy	LD1 Variance	LD2 Variance	Cumulative LD1+LD2
**Category (4 classes)**	800	50.0%	93.1%	4.9%	98.0%
**Compound (16 classes)**	3,102	53.4%	66.0%	20.1%	86.1%

Supplementary Table S17. Complete LD1 discriminant coefficients for the category-level LDA (LD1 = 93.1% of between-class variance). The 4.8-fold difference in absolute coefficient magnitude between ΔGSOLV (|−205.24|) and ΔGGAS (|−42.41|) quantifies the paradigm shift from unsupervised to supervised energetic discrimination.

Rank	Component	LD1 Coefficient	Sign	Mechanistic Interpretation
**1**	ΔGSOLV	−205.24	Neg.	Primary class discriminant; solvation energy differential in T790M polar pocket
**2**	ΔEEL	−130.02	Neg.	Coulombic electrostatics; stronger charge–charge interactions → more positive LD1
**3**	ΔEGB	+84.76	Pos.	Born desolvation penalty; more polar ligands pay higher cost → penalized in LD1
**4**	ΔGTOTAL	+65.71	Pos.	Net binding affinity; better binders carry more positive LD1 projection
**5**	ΔGGAS	−42.41	Neg.	Gas-phase total;
**6**	ΔVDWAALS	−29.59	Neg.	Van der Waals; shape complementarity — tertiary discriminant
**7**	ΔESURF	+3.78	Pos.	Nonpolar solvation; negligible contribution to between-class separation

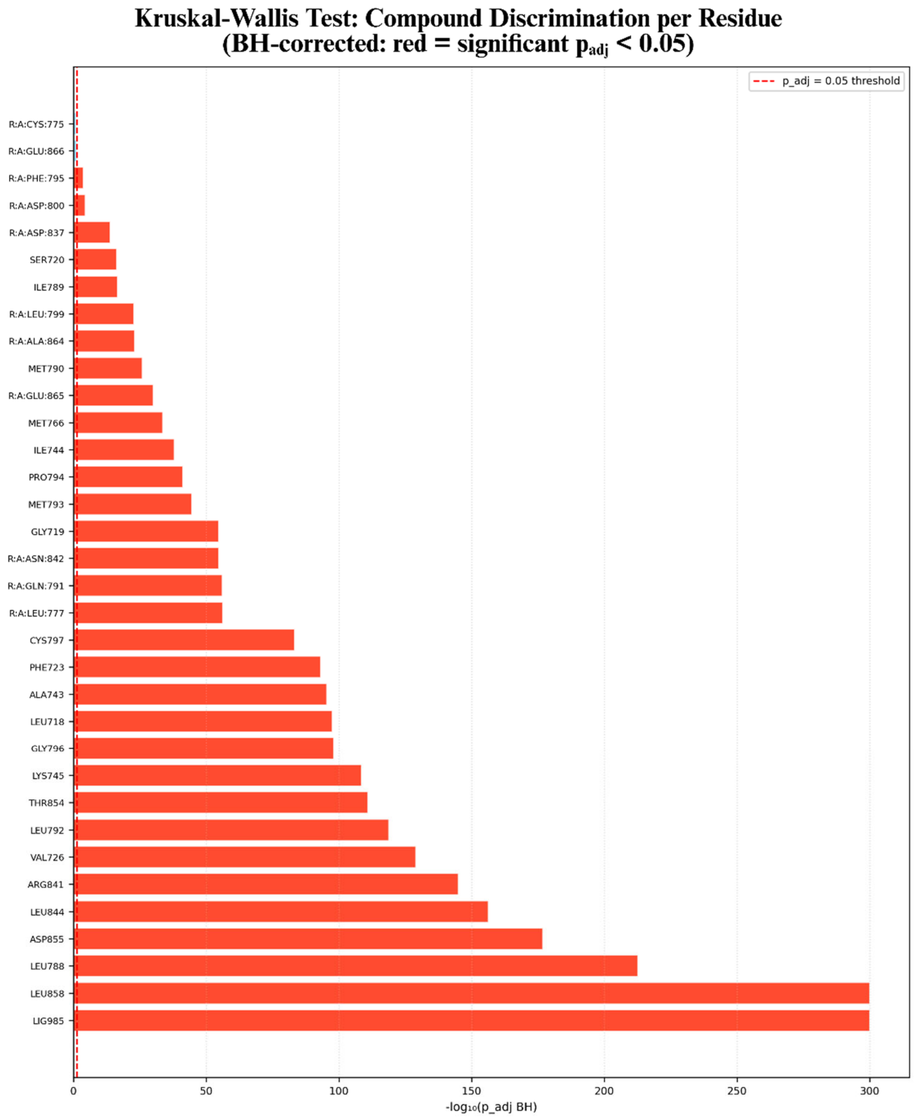

Supplementary Figure S16. Kruskal-Wallis statistical evaluation of compound-specific discriminatory power per residue within the EGFR T790M/V948R binding pocket. The bar chart quantifies the capacity of specific amino acid residues to differentiate the thermodynamic binding behavior across the 16 evaluated phytochemicals and control inhibitors. **Statistical Significance Scale:** The horizontal axis displays the log10 of the Benjamini-Hochberg (BH) adjusted p-values (p_{adj}). Extended bars represent smaller p-values, indicating a higher degree of statistical significance and stronger evidence that the compounds interact distinctly at that specific structural locus. **Significance Threshold:** The vertical dashed red line marks the standard significance threshold ($p_{adj} = 0.05$). Notably, 32 out of the 34 evaluated residues significantly exceed this threshold, confirming robust mechanistic differentiation across the dataset. **High Discrimination Residues (Thermodynamic Filters):** LIG985 (representing intrinsic ligand strain) and LEU858 display the maximum discriminatory capacity, identifying them as the primary thermodynamic filters that dictate competitive binding affinity. Furthermore, massive discrimination at ASP855 and LEU844 underscores the critical role of the activation loop (DFG motif) in separating highly potent inhibitors from weaker binders. **Universal Barriers vs. Selectivity Drivers:** While the T790M gatekeeper (MET790) imposes a biologically critical binding barrier, its comparatively shorter bar indicates that all compounds uniformly incur its steric penalty. Consequently, ultimate compound selectivity is not determined by the gatekeeper itself, but rather by the highly discriminant peripheral residues like LEU858 and ASP855. **Methodological Rigor:** The application of the Benjamini-Hochberg correction strictly controls the false discovery rate (FDR at 5%) across multiple comparisons. This ensures that the observed residue-level variations reflect genuine physical and mechanistic distinctions in ligand binding rather than statistical artifacts.

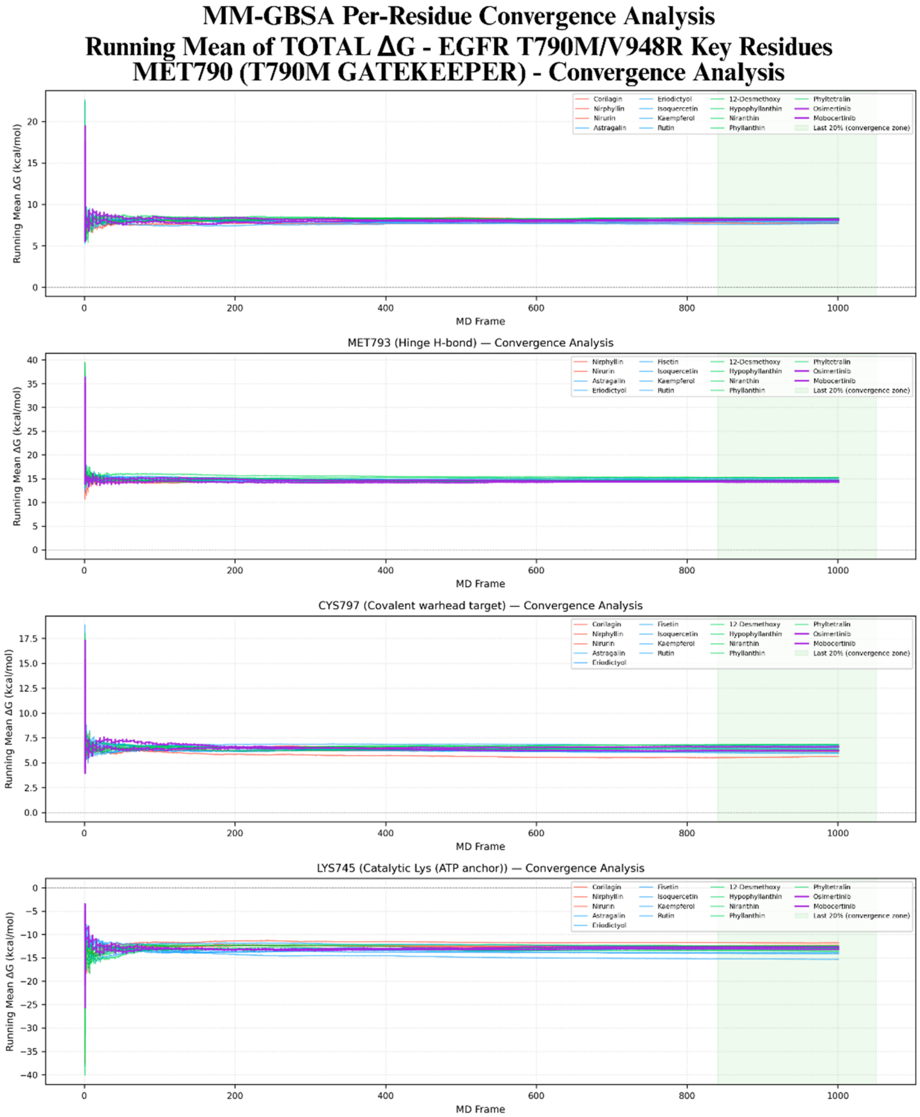

Supplementary Figure S17. Running mean convergence analysis of per-residue MM-GBSA binding free energies (Delta G) for critical EGFR T790M/V948R interaction targets. The multi-panel plot tracks the cumulative average of thermodynamic contributions across the molecular dynamic's trajectory (1000 frames) to validate the statistical reliability and equilibrium state of the energetic interactions. **Trajectory Dynamics and Convergence Zone:** Initial structural accommodation is reflected in early trajectory fluctuations, which systematically resolve into a horizontal plateau, confirming that the system has reached a stable and reproducible thermodynamic equilibrium. The shaded green region designates the final 20% of the simulation. Convergence is quantitatively satisfied when the difference between the mean Delta G within this terminal zone and the global trajectory mean is < 0.1 kcal/mol. **MET790 (T790M Gatekeeper):** Running means robustly converge to positive values (approximately +8 kcal/mol), accurately capturing the constant thermodynamic barrier and steric accommodation required by the resistance mutation. **MET793 (Hinge H-bond):** Demonstrates pronounced trajectory stability, converging consistently near +15 kcal/mol across the dataset, reflecting the universal structural strain and desolvation cost associated with accessing the kinase hinge region. **CYS797 (Covalent Warhead Target):** Trajectories converge with high consistency around +6 kcal/mol for all evaluated inhibitors, validating the stable nature of the non-covalent contacts at this critical residue. **LYS745 (Catalytic Lys):** Serving as the primary ATP-anchoring determinant, this panel demonstrates highly favorable and stable convergence trajectories between -10 and -15 kcal/mol. This rigorous plateau provides critical statistical assurance that the robust electrostatic anchoring observed for the *Phyllanthus niruri* phytochemicals is a genuine, sustained thermodynamic state rather than a transient sampling artifact.

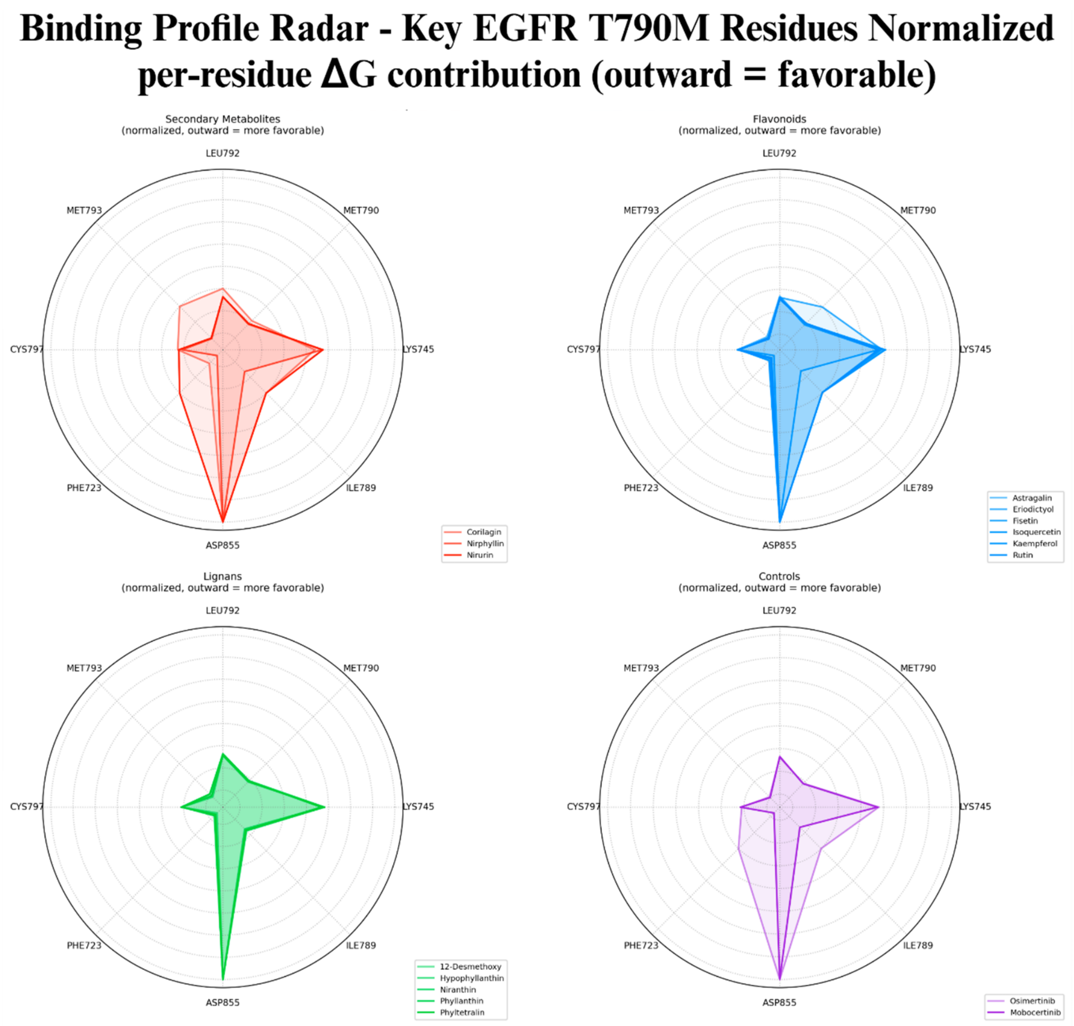

Supplementary Figure S18. Normalized pharmacological binding fingerprints of *Phyllanthus niruri* phytochemicals and clinical control inhibitors within the EGFR T790M/V948R active site. Radial radar plots map the multi-residue MM-GBSA interaction profiles across eight structurally critical amino acid targets to establish distinct compound class signatures. **Data Normalization:** Radial axes represent the normalized per-residue free energy contributions (Delta G). Outward radial projection strictly indicates thermodynamically favorable target stabilization (negative Delta G), whereas inward convergence towards the center reflects weak interactions or thermodynamic penalties. **Class-Specific Profiling:** Compounds are delineated by chemical classification: Secondary Metabolites (red), Flavonoids (blue), Lignans (green), and irreversible Controls (purple). This arrangement establishes a visual baseline utilizing the "gold standard" commercial drugs (osimertinib and mobocertinib) to evaluate the geometric and energetic mimicry achieved by the natural scaffolds. **Mechanistic Insights:** The fingerprints intuitively demonstrate how *Phyllanthus niruri* ligands compensate for their inability to form irreversible covalent bonds at CYS797. By forming robust, non-covalent multiresidue networks, these phytochemicals achieve competitive spatial overlaps with the commercial controls. Specifically, the secondary metabolite corilagin displays an exceptionally pronounced outward projection toward the DFG-motif (ASP855), representing an electrostatic stabilization that vastly exceeds that of the clinical reference compounds at this locus. Concurrently, the flavonoid class demonstrates highly optimized anchoring at the catalytic lysine (LYS745). Ultimately, this multidimensional mapping visually confirms that natural scaffolds achieve high affinity by maximizing peripheral stabilizing contacts rather than relying on hinge or covalent warhead interactions.

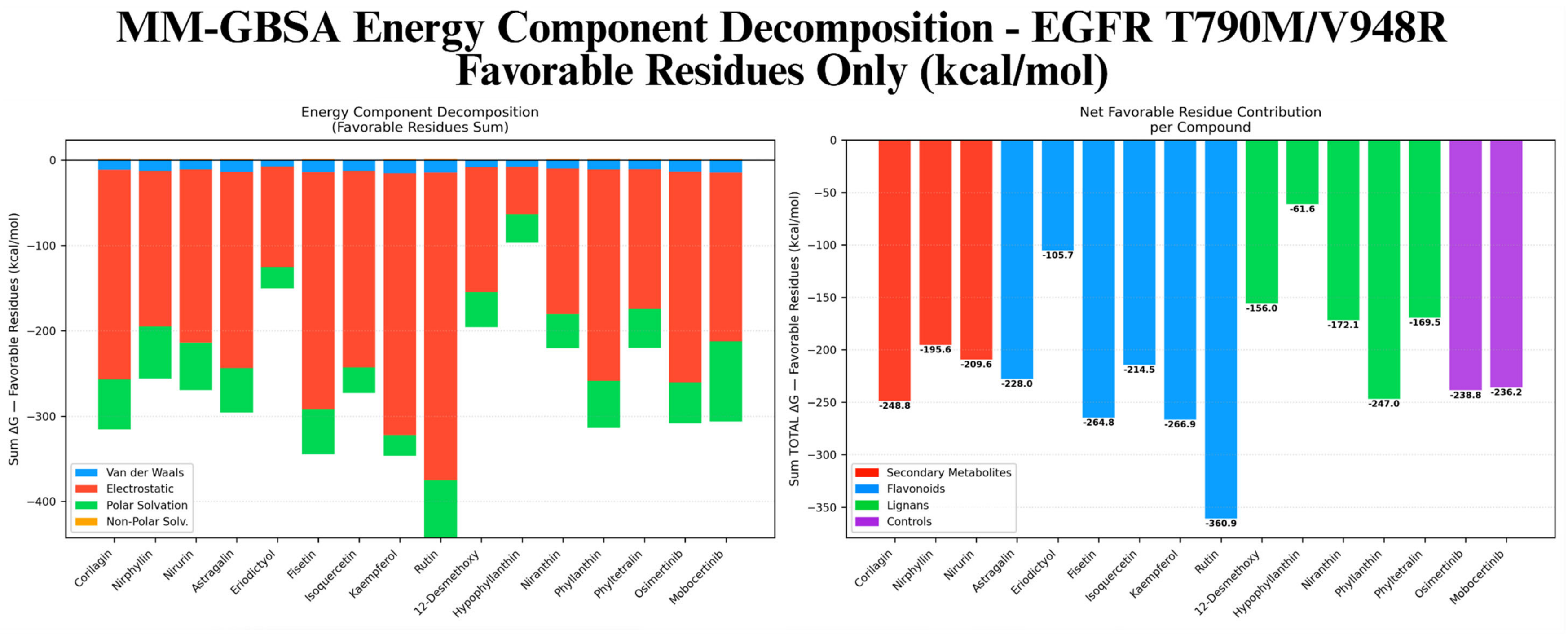

Supplementary Figure S19. MM-GBSA energy component decomposition and net favorable residue contributions for *Phyllanthus niruri* phytochemicals and clinical control inhibitors against EGFR T790M/V948R. This analysis specifically isolates the cumulative thermodynamic contributions of favorable binding site residues (ΔG < 0) to mechanistically validate the high binding affinities observed for natural product scaffolds. **(Left) Energy Component Decomposition:** A stacked bar chart dissecting the sum of favorable per-residue interactions into fundamental physical forces: electrostatic (red), van der Waals (blue), polar solvation (green), and non-polar solvation (orange). Electrostatic interactions overwhelmingly dominate the thermodynamic landscape across almost all compounds. This massive electrostatic accumulation, particularly evident in rutin and corilagin, reflects the extensive formation of hydrogen-bonding networks and charge-charge attractions driven by the polyhydroxylated structures of flavonoids and tannins. This visually demonstrates that the high overall binding free energies are not computational artifacts, but the result of additive, physically grounded electrostatic interactions within the ATP-binding pocket. **(Right) Net Favorable Residue Contribution:** The total cumulative favorable energy per compound, categorized by pharmacological class: Secondary Metabolites (red), Flavonoids (blue), Lignans (green), and covalent Controls (purple). Rutin exhibits the highest net favorable sum in the dataset at -360.9 kcal/mol, while the primary lead candidate corilagin reaches -248.8 kcal/mol. The leading lignan, phyllanthin, demonstrates a sum of -247.0 kcal/mol. Notably, the non-covalent energetic profiles of these natural compounds consistently match or exceed those of the approved irreversible inhibitors osimertinib (-238.8 kcal/mol) and mobocertinib (-236.2 kcal/mol), providing quantitative validation of their competitive structural affinities for the mutant kinase domain.

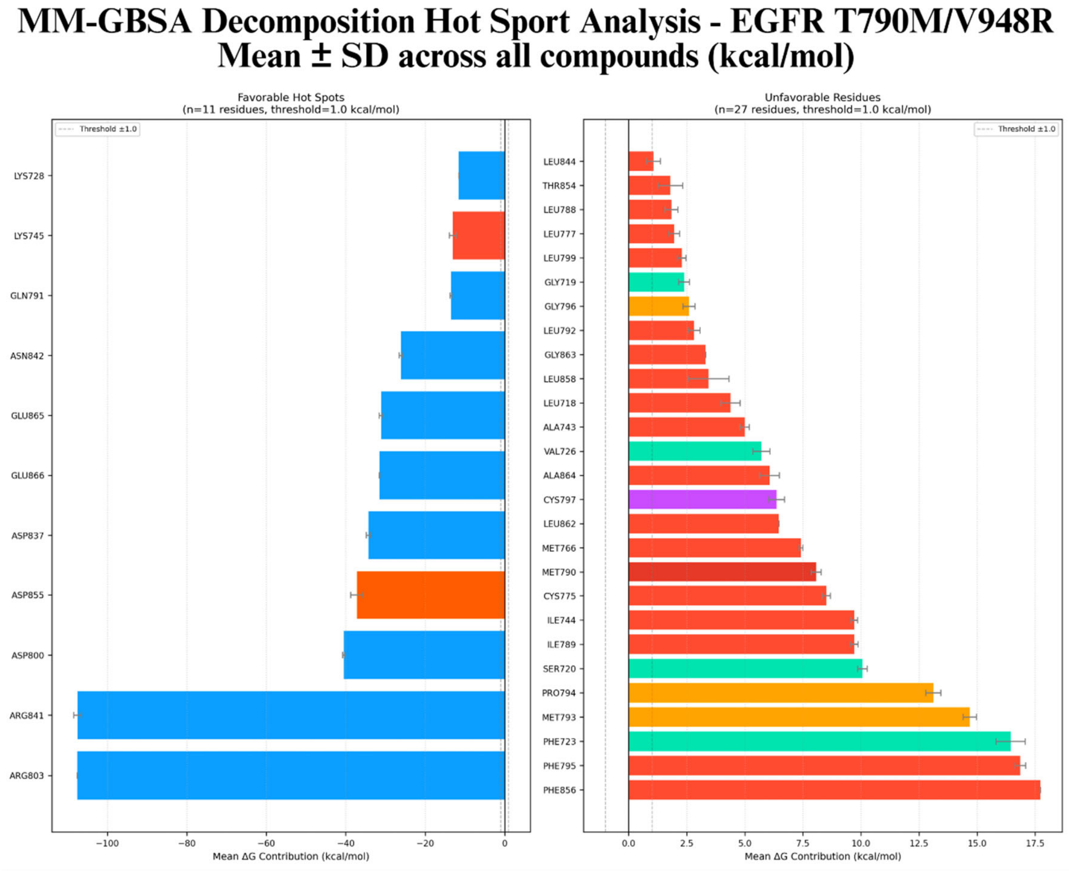

Supplementary Figure S20. Per-residue MM-GBSA binding free energy decomposition and hot spot functional classification for the EGFR T790M/V948R kinase domain. The dual-panel bar chart illustrates the mean total thermodynamic contributions (ΔG) of key binding site residues, classifying them by functional structural regions.

(Left) Favorable Hot Spots: Residues exhibiting negative energy contributions (ΔG < -1.0 kcal/mol) actively stabilize ligand binding and drive thermodynamic affinity. Notably, ARG841 and ARG803 function as critical electrostatic anchors, displaying exceptionally high favorable contributions exceeding -100 kcal/mol. **(Right) Unfavorable Residues:** Residues displaying positive free energy values (ΔG > +1.0 kcal/mol) represent thermodynamic penalties, structural strain, or steric repulsion that destabilize the complex. Within this established framework, residues such as PHE856 and PHE795 account for the most pronounced unfavorable desolvation or reorganization costs. **Functional Color Coding:** Bar colors denote specific functional classifications within the ATP-binding pocket. **Red/Dark Brown** identifies critical regulatory and catalytic residues, specifically the T790M gatekeeper (MET790) and the catalytic ATP-anchor lysine (LYS745). **Orange/Yellow** highlights the hinge region (MET793, PRO794, GLY796), a vital structural zone that establishes hydrogen-bonding networks with inhibitors. **Purple** specifically designates CYS797, the nucleophilic target essential for covalent bond formation in third-generation inhibitors like osimertinib and mobocertinib. **Teal/Cyan** indicates the highly flexible, glycine-rich P-loop residues (PHE723, VAL726, SER720). **Steel Blue** represents general structural or hydrophobic framework residues. **Significance Thresholds:** Vertical dashed lines indicate the consensus computational hot spot threshold at | Mean TOTAL| ≥ 1.0 kcal/mol. Any residue exceeding this absolute value is considered a critical interaction node (favorable or unfavorable) essential for structure-based drug design.

Supplementary Table S18. MM-GBSA energy component decomposition for all 16 compounds against EGFR T790M/V948R (PDB 7A6K). Sum_Elec and Sum_vdW = sums of electrostatic and van der Waals contributions from all favorable hot spots (kcal/mol); pct_Elec/pct_vdW = percentage of |Sum_Elec|+|Sum_vdW|; #HS_fav = number of favorable hot spots (ΔGTOTAL ≤ −1.0 kcal/mol). (*) = non-convergent residues present (ARG841/Nirphyllin).

Compound	Category	Sum_Elec (kcal/mol)	Sum_vdW (kcal/mol)	pct_Elec%	pct_vdW%	#HS_fav
**Rutin**	Flavonoids	−360.33	−15.16	96.0	4.0	7
**Kaempferol**	Flavonoids	−306.38	−15.66	95.1	4.9	7
**Fisetin**	Flavonoids	−278.15	−14.07	95.2	4.8	7
**Phyllanthin**	Lignans	−247.81	−10.98	95.8	4.2	6
**Osimertinib**	Controls	−247.07	−13.60	94.8	5.2	6
**Corilagin**	Sec. Metabolites	−245.51	−11.71	95.4	4.6	6
**Isoquercetin**	Flavonoids	−230.47	−12.61	94.8	5.2	5
**Astragalin**	Flavonoids	−229.95	−13.70	94.4	5.6	6
**Nirurin**	Sec. Metabolites	−202.71	−10.98	94.9	5.1	5
**Mobocertinib**	Controls	−197.75	−14.87	93.0	7.0	6
**Nirphyllin***	Sec. Metabolites	−182.24	−12.68	93.5	6.5	5
**Niranthin**	Lignans	−170.38	−10.09	94.4	5.6	4
**Phyltetralin**	Lignans	−163.57	−10.83	93.8	6.2	4
**2,3-Desmethoxy seco-isolintetralin**	Lignans	−146.09	−8.48	94.5	5.5	3
**Eriodictyol-7-rhamnopyranoside**	Flavonoids	−117.99	−7.66	93.9	6.1	4
**Hypophyllanthin**	Lignans	−55.43	−8.01	87.4	12.6	3

Supplementary Table S19. Key pharmacological residues: per-residue MM-GBSA ΔGTOTAL (kcal/mol), Kruskal-Wallis statistical discrimination (Benjamini-Hochberg corrected p-values), and dominant energy component for 16 compounds against EGFR T790M/V948R (PDB 7A6K). (*) = non-convergent residue pairs present. N = number of compounds with data.

Residue	Function	Mean ΔGTOTAL (kcal/mol)	SD	N	KW p_{adj}	Dominant component
**ARG841**	αG helix	−107.52	0.97	15	1.01×10^{-145}	ΔG_{EL} (−98.76 to −109.75)
**ASN842***	Post-DFG loop	−26.12	0.44	10	1.61×10^{-55}	ΔG_{EL}
**ASP855**	DFG motif	−37.17	1.55	17	1.35×10^{-177}	ΔG_{EL}; Corilagin (−64.49)
**GLN791**	Hinge H-bond acceptor	−13.51	0.22	11	8.05×10^{-57}	ΔG_{EL}
**LYS745**	Catalytic Lys	−13.06	0.85	17	2.33×10^{-109}	$\Delta G_{PolarSolv}$ (−15 to −23)
**PHE723**	P-loop	+16.45	0.63	15	6.11×10^{-94}	Internal strain
**MET793**	Hinge	+14.68	0.28	16	1.91×10^{-45}	Internal
**PRO794**	Hinge Pro	+13.12	0.32	8	4.78×10^{-42}	Internal strain
**MET790**	T790M Gatekeeper	+8.07	0.22	16	1.08×10^{-26}	Internal (+9.2–9.9); vdW (−4.1 to −4.6)
**CYS797**	Warhead target	+6.38	0.34	17	4.79×10^{-84}	Internal (+6.92–7.27); ΔG_{EL} (+3.31–4.47)

Supplementary Table S20. Condensed Fukui functions (f^+, f^-, f^0) and dual descriptor (CDD = $f^+ - f^-$) from Hirshfeld population analysis at the B3LYP-D3(BJ)/def2-TZVP level (Multiwfn 3.8). For phyltetralin, all Fukui values are negative owing to the Hirshfeld partitioning of a highly symmetric scaffold with near-zero net charge transfer between the N and N±1 states; the relative magnitudes retain physical meaning for site ranking within the molecule (Lu & Chen, 2012). Only atoms with |CDD| ≥ 0.010 or |f^+/f^-| ≥ 0.010 are shown.

Corilagin

Atom	f^+	f^-	f^0	CDD
**C7**	–	0.0195	–	–
**C16**	–	0.0187	–	–
**C17**	–	0.0224	–	–
**C18**	–	0.0418	–	–
**C43 (ester C=O)**	–	–	–	+0.0394
**C44 (galloyl C=O)**	–	0.0503	–	–
**O27**	–	0.0268	–	–
**O36 (catecholate O)**	0.0843	–	–	–

Eriodictyol-7-rhamnopyranoside

Atom	f^+	f^-	f^0	CDD
**C12 (catechol B-ring)**	–	0.1047	–	–
**O22 (catechol OH)**	–	0.1136	–	−0.1088
**C31 (rhamnosyl C-2′)**	0.1416	–	–	–

Osimertinib

Atom	f^+	f^-	f^0	CDD
**C28 (acrylamide β-C)**	0.2365	–	–	+0.0836
**N1 (quinazoline)**	–	0.0375	–	–

Phyltetralin (all values negative; see note above)

Atom	f^+	f^-	f^0	CDD
**C6**	–	–	–	+0.0577
**C8**	–	–	–	–
**C10**	–	–	–	–
**C11**	–	−0.0707	–	+0.0482
**O15**	−0.0506	–	–	–

Rutin

Atom	f^+	f^-	f^0	CDD
**C42 (catechol B-ring)**	–	0.0849	–	–
**C43 (catechol B-ring)**	–	0.0897	–	+0.0726
**O24 (catechol OH)**	–	0.0953	–	–

Supplementary Table S21. Hirshfeld orbital composition analysis for the five studied compounds at the B3LYP-D3(BJ)/def2-TZVP level (Multiwfn 3.8). Only atoms contributing ≥ 5 % to the respective orbital are listed. ODI = orbital delocalization index.

Corilagin

Orbital	Energy (eV)	ODI	Key contributions
**HOMO (orbital 164)**	−6.168	11.86	C18 (20.4 %), O36 (16.0 %), C7 (13.4 %), C17 (11.0 %), C16 (9.1 %) — galloyl ring A: 83.2 %
**HOMO−1 (orbital 163)**	−6.186	6.22	C11 (12.5 %), O31 (9.8 %), C1 (8.4 %), C10 (7.2 %), C12 (7.3 %) — galloyl ring B

ΔE(HOMO − HOMO−1) = 0.017 eV (0.39 kcal mol^{-1}).

Eriodictyol-7-rhamnopyranoside

Orbital	Energy (eV)	ODI	Key contributions
**HOMO (orbital 114)**	−6.020	12.16	C12 (17.1 %), C10 (15.0 %), C7 (14.3 %), O22 (13.7 %), C9 (12.2 %), O21 (10.6 %) — catechol B-ring: 88.0 %; rhamnosyl: 0.0 %
**LUMO (orbital 115)**	−1.780	11.85	C31 (rhamnosyl C-2′) contributes 23.5 % of LUMO density

Osimertinib

Orbital	Energy (eV)	ODI	Key contributions
**HOMO (orbital 133)**	−5.226	6.77	C29 (11.8 %), C3 (11.6 %), N5 (10.7 %), C23 (7.7 %) — N-heterocyclic core
**LUMO (orbital 134)**	−1.419	12.43	Acrylamide group: C28 (23.65 %), C25 (16.16 %), C27 (15.06 %), O26 (11.18 %) — total 66.0 %
**HOMO−1 (orbital 132)**	−5.527	23.26	N35 (dimethylamino): 45.9 %

Phyltetralin

Orbital	Energy (eV)	ODI	Key contributions
**HOMO (orbital 112)**	−5.351	10.12	C1 (14.0 %), C2 (13.5 %), C7 (13.2 %), C9 (13.2 %), O15 (11.0 %), O16 (11.0 %) — total 75.9 %

Rutin

Orbital	Energy (eV)	ODI	Key contributions
**HOMO (orbital 160)**	−5.877	6.32	C41 (11.84 %), C43 (10.2 %), O24 (8.7 %), C42 (7.1 %) — quercetin chromone
**HOMO−1 (orbital 159)**	−6.265	7.13	C5 (15.9 %), C8 (10.5 %) — A-ring resorcinol region
**LUMO (orbital 161)**	−1.868	8.09	C42 (16.0 %), C43 (14.5 %), O24 (10.8 %) — catechol-C=O region: 48.1 %

ΔE(HOMO − HOMO−1) = 0.388 eV.

Intramolecular Non-Covalent Interactions: NCI/RDG Plots

A. Corilagin

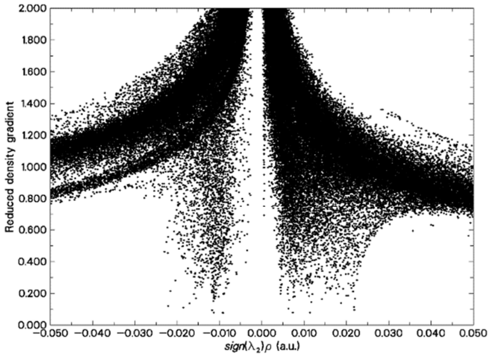

B. Eridictyol-7-rhamnopyranoside

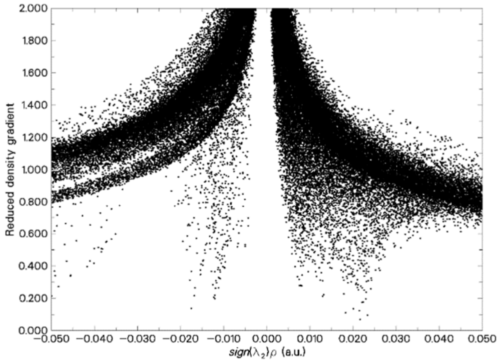

C. Phyltetralin

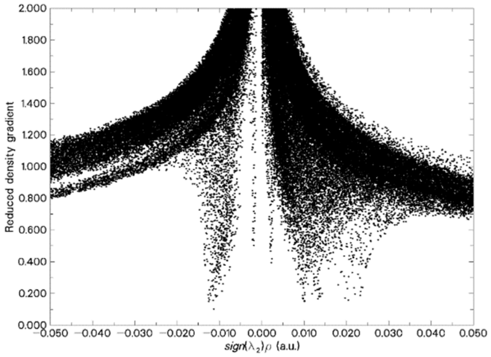

D. Rutin

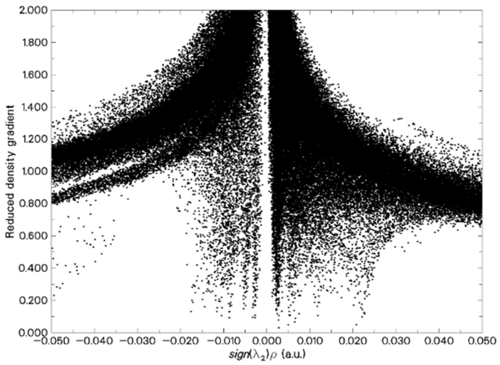

E. Osimertinib

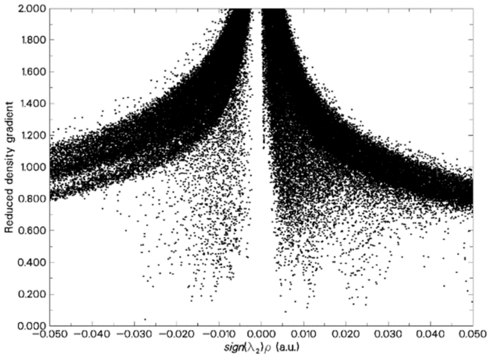

Supplementary Figure S21. Reduced Density Gradient (RDG) versus $\mathrm{sign}(\lambda_2)\rho$ scatter plots characterizing the intramolecular non-covalent interactions (NCI) of the isolated phytochemical leads and osimertinib. These analyses are derived from gas-phase wavefunctions to evaluate intrinsic conformational preorganization, where spikes approaching RDG} to 0 in the negative range (left branch) indicate attractive interactions (e.g., hydrogen bonds, van der Waals contacts), and positive values (right branch) represent steric repulsions.
